# What are the consequences of combining nuclear and mitochondrial data for phylogenetic analysis? Lessons from *Plethodon *salamanders and 13 other vertebrate clades

**DOI:** 10.1186/1471-2148-11-300

**Published:** 2011-10-13

**Authors:** M Caitlin Fisher-Reid, John J Wiens

**Affiliations:** 1 Department of Ecology and Evolution, Stony Brook University, Stony Brook NY, 11794-5245, USA

## Abstract

**Background:**

The use of mitochondrial DNA data in phylogenetics is controversial, yet studies that combine mitochondrial and nuclear DNA data (mtDNA and nucDNA) to estimate phylogeny are common, especially in vertebrates. Surprisingly, the consequences of combining these data types are largely unexplored, and many fundamental questions remain unaddressed in the literature. For example, how much do trees from mtDNA and nucDNA differ? How are topological conflicts between these data types typically resolved in the combined-data tree? What determines whether a node will be resolved in favor of mtDNA or nucDNA, and are there any generalities that can be made regarding resolution of mtDNA-nucDNA conflicts in combined-data trees? Here, we address these and related questions using new and published nucDNA and mtDNA data for *Plethodon *salamanders and published data from 13 other vertebrate clades (including fish, frogs, lizards, birds, turtles, and mammals).

**Results:**

We find widespread discordance between trees from mtDNA and nucDNA (30-70% of nodes disagree per clade), but this discordance is typically not strongly supported. Despite often having larger numbers of variable characters, mtDNA data do not typically dominate combined-data analyses, and combined-data trees often share more nodes with trees from nucDNA alone. There is no relationship between the proportion of nodes shared between combined-data and mtDNA trees and relative numbers of variable characters or levels of homoplasy in the mtDNA and nucDNA data sets. Congruence between trees from mtDNA and nucDNA is higher on branches that are longer and deeper in the combined-data tree, but whether a conflicting node will be resolved in favor mtDNA or nucDNA is unrelated to branch length. Conflicts that are resolved in favor of nucDNA tend to occur at deeper nodes in the combined-data tree. In contrast to these overall trends, we find that *Plethodon *have an unusually large number of strongly supported conflicts between data types, which are generally resolved in favor of mtDNA in the combined-data tree (despite the large number of nuclear loci sampled).

**Conclusions:**

Overall, our results from 14 vertebrate clades show that combined-data analyses are not necessarily dominated by the more variable mtDNA data sets. However, given cases like *Plethodon*, there is also the need for routine checking of incongruence between mtDNA and nucDNA data and its impacts on combined-data analyses.

## Background

The field of molecular phylogenetics is heading towards an exciting future. In this future, genomics will allow for the use of dozens of unlinked nuclear loci to estimate phylogenies [e.g. [1-5]]. These data may then be analyzed using species-tree methods that use principles of population genetics to resolve incongruence among loci (e.g., BEST [6]; STEM [7]; *BEAST [8]).

But even as the field of phylogenetics seems to be moving towards such a future, it is clearly not there yet. For example, in animals, many phylogenies continue to be estimated based on mitochondrial (mtDNA) data alone [e.g. [9-12]], or a combined (concatenated) analysis of nuclear (nucDNA) and mtDNA data [e.g. [13-18]]. In many cases, these analyses of mtDNA or concatenated data may be necessary because sampling many species makes it impractical to include many nuclear loci (and due to fiscal constraints), and sampling many species and/or few loci makes it impractical to utilize explicit species-tree methods (despite strong theoretical justification for their use; e.g., [6,8,19]). Many review papers have addressed the pros and cons of mtDNA data [e.g. [20-24]], and many empirical studies have suggested the need for caution in their use [e.g. [25-27]]. However, most reviews have focused on the use of mtDNA in phylogeographic studies [e.g. [23,24,28]] and on the question of whether mtDNA should be used in phylogenetics at all [e.g. [22]].

Here, we address a somewhat different question. Given that many systematists routinely estimate phylogenies using combined mtDNA and nucDNA, we ask: what are the consequences of the common practice of combining these two types of data? For example, will the combined-data tree tend to resemble the mtDNA tree due to larger numbers of variable mtDNA characters? Or will the combined-data tree contain a mixture of clades favored by the separate data sets? Are there any generalities that can be made about when mtDNA or nucDNA data will be favored in particular clades or data sets? These questions are particularly important because many published studies simply present trees from combined analyses of mtDNA and nucDNA, without any examination of whether the mtDNA and nucDNA trees are congruent, or to what extent the combined-data tree reflects the contributions of each data set [e.g. [14-18], but see for example [29]]. In fact, if combined-data trees are often discordant with trees from nucDNA and largely reflect the mtDNA data instead, there may be little to be gained by collecting and adding nucDNA data in the first place (i.e., if trees are estimated from the combined-data and nucDNA have negligible impact on the combined-data analysis). To our knowledge, these important questions have never been the subject of a focused study.

In this paper, we address these and related questions, by evaluating combined-data analyses that utilize both mtDNA and nucDNA data. We approach these questions using new data and analyses for *Plethodon *salamanders, along with new analyses of existing data sets from 13 other vertebrate groups. Below, we describe the four main questions (and five associated predictions) that we address. For each of the four main questions, we are attempting to discern if there are generalities that can be made regarding the interaction of mtDNA and nucDNA data sets in a combined-data analysis.

First, are there frequent conflicts between separate mtDNA and nucDNA trees, and are the conflicting clades strongly supported by each data set? Weakly supported conflicts may be spurious and thus not problematic, whereas strongly supported conflicts may reflect more serious issues (such as long-branch attraction or discordance between gene and species trees) that may confound combined analyses [e.g. [6,30-34]]. As a working hypothesis, we predict that (i) discordance between mtDNA and nucDNA will generally be uncommon, and if found, will often be weakly supported by one or both data sets. This prediction is based on the simple expectation that both mitochondrial and nuclear genes will frequently share the same underlying phylogenetic history (especially given that smaller effective population sizes of mitochondrial genes may reduce discordance due to incomplete lineage sorting [21]), and that incongruence may often be due to estimated phylogenies that do not fully match the underlying gene trees [30-32].

Second, are conflicts between the separate mtDNA and nucDNA trees generally resolved in favor of mtDNA or nucDNA in the combined-data tree? Mitochondrial genes are generally thought to evolve more rapidly than nuclear genes, and so should have more variable characters but should also have more homoplasy [e.g. [21,22]]. In general, we expect conflicts between data sets to be resolved in favor of the data set with more variable characters, but also with less homoplasy. A data set with extensive conflict among characters (i.e., high homoplasy due to random noise from high overall rates of character change) may be less likely to overturn relationships inferred from a data set with less internal conflict among characters. Thus, the resolution of conflicts between mtDNA and nucDNA data sets in the combined-data tree may vary from analysis to analysis, depending on the number of characters sampled in each data set and their levels of variability and homoplasy. We predict that (ii) when mtDNA dominates a combined-data tree, it will be due to larger numbers of variable characters compared to nucDNA, and (iii) when nucDNA dominates a combined-data tree, it will be due to lower levels of homoplasy compared to mtDNA.

We address these predictions by first comparing the number of nodes shared between trees from mtDNA, nucDNA, and the combined-data, across 14 vertebrate clades. Next, we test if the proportion of nodes shared between the combined-data and mtDNA trees is correlated with the overall proportion of the variable sites in the combined data that are from mtDNA (given the prediction that the data set with more variable characters will have a stronger influence on the combined-data tree). We also test if the resolution of conflicts in the combined-data tree is related to the level of homoplasy in the mtDNA versus nucDNA data sets, given the prediction that the combined-data tree will be resolved in favor of the data set with less homoplasy (i.e., nucDNA) regardless of the relative numbers of variable sites.

Third, what generalities, if any, can we make about which nodes of the combined-data tree are resolved in favor of mtDNA vs. nucDNA? We expect that the resolution of nodes in the combined-data tree may depend on the underlying branch lengths and the depth of those branches in the tree. We predict (iv) mtDNA and nucDNA will be more congruent on longer branches, because allele histories should coalesce on longer branches, reducing discordance among genes due to incomplete lineage sorting [35]. Furthermore, introgression is less likely among more distantly related species (i.e., separated by longer branches), due to the accumulation of reproductive isolating mechanisms over time [36], which should also contribute to greater congruence between mtDNA and nucDNA on longer branches (especially if mitochondrial introgression is an important source of discordance between mtDNA and nucDNA trees; e.g., [25]). Longer branches may also be more congruent if they tend to be more strongly supported by each gene [4], reducing spurious conflicts between mtDNA and nucDNA due to weak support. We expect shorter branches to be resolved in favor of mtDNA, given that there may be too little time for mutations to accumulate on the shortest branches for slower-evolving nuclear genes. In addition, there may be extensive incongruence among nuclear genes on short branches due to incomplete lineage sorting, also leading to weaker branch support [e.g. [4]]. In contrast, the mitochondrial genome is a single locus (such that there should be no incongruence among histories of mitochondrial genes), and incomplete lineage sorting may be less problematic at the between-species level due to the generally smaller effective population size of the mitochondrial genome [e.g. [20,22,37]].

Finally, when mtDNA and nucDNA trees conflict, we predict (v) that nucDNA may be more likely to win conflicts deeper in the combined-data tree, while mtDNA may win resolutions that are shallower [e.g. [38,39]]. Clades deep in the tree may be harder to resolve due to long-branch attraction [40], and faster evolving genes (like mtDNA) will likely exacerbate problems of long-branch attraction (i.e., branch lengths may generally tend to be longer). The importance of tree depth may depend not only on the relative placement of branches in the tree, but also on overall branch lengths (with mtDNA being more problematic when branches are generally longer). The potential for nucDNA data to better resolve deep branches may be an important justification for including these data in the first place, along with the desire to sample unlinked loci.

In summary, a consideration of general principles suggests conflicts between mtDNA and nucDNA may be uncommon and weakly supported, and that the resolution of conflicting nodes in the combined analysis (i.e., favoring mtDNA vs. nucDNA) may vary based on the number of variable characters and level of homoplasy in each mtDNA and nucDNA data set, the lengths of branches, and the depths of branches in the tree. We test these predictions empirically here, using new data from *Plethodon *salamanders and published data from 13 other vertebrate clades.

*Plethodon *is the most species-rich genus of North American salamanders [41]. They are terrestrial, direct-developing salamanders that are generally common and diverse in North American forests [42]. *Plethodon *have long interested evolutionary biologists and ecologists, and hundreds of papers have been published on *Plethodon *in diverse areas, including studies of behavior, [e.g. [43-46]], community ecology [e.g. [47-49]], patterns of trait evolution [e.g. [13,50]], speciation and hybridization [e.g. [51-58]], and response to environmental change [e.g. [59-61]]. Many of these studies have used a phylogenetic approach, making a reliable phylogeny for *Plethodon *particularly important.

Earlier studies addressed *Plethodon *phylogeny using data from allozymes [e.g. [52,53]] and mtDNA [e.g. [56]], whereas more recent studies have combined mtDNA and nucDNA data [e.g., [13,57]]. In general, these studies have yielded similar estimates of higher-level *Plethodon *phylogeny (e.g., most agree on a split between eastern and western species, and on the species groups in eastern North America). However, there have been substantive disagreements between studies regarding some species-level relationships (e.g., within the *cinereus *group; [13]). Furthermore, all previous studies used relatively few nuclear loci (two or three; [13,57,61]). Here we obtain new data from five nuclear loci and combine these with existing data from four nuclear genes and three mitochondrial genes, and use these data to address *Plethodon *phylogeny and general questions about combining mtDNA and nucDNA in phylogenetic studies.

## Results

### *Plethodon *phylogeny

**Figure 1:**
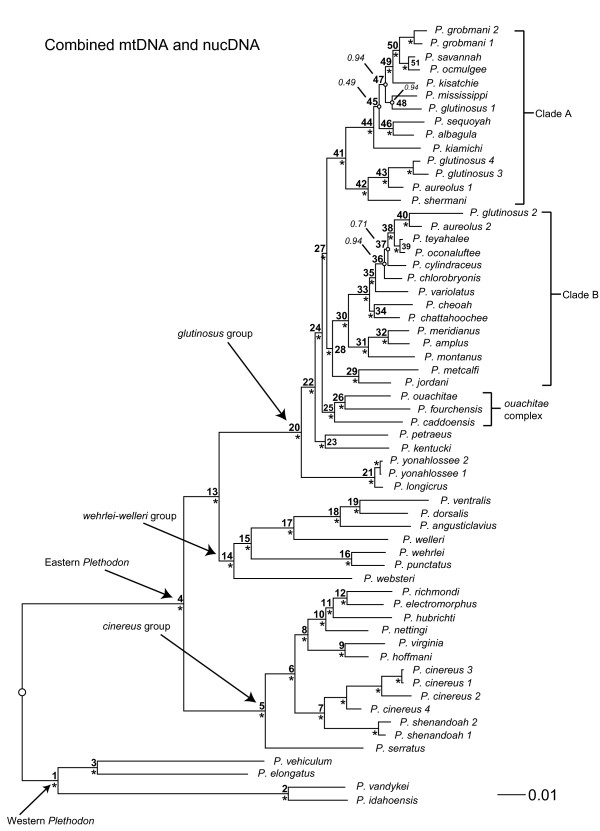
**Combined-data *Plethodon *phylogeny**. Phylogeny of the salamander genus *Plethodon *based on a combined, partitioned Bayesian analysis of mitochondrial DNA (mtDNA) and nuclear DNA (nucDNA). An asterisk next to a node indicates strong support (Pp ≥ 0.95). Small open circles on a node indicate Pp < 0.95, and these values are listed. Integers next to each node correspond to clade numbers used in analyses of congruence and discordance. A clade was not numbered if all terminal taxa belong to the same species. The outgroup taxa are excluded (but only from the figure) to facilitate presentation of branch lengths, and the root is indicated with a large open circle.

**Figure 2:**
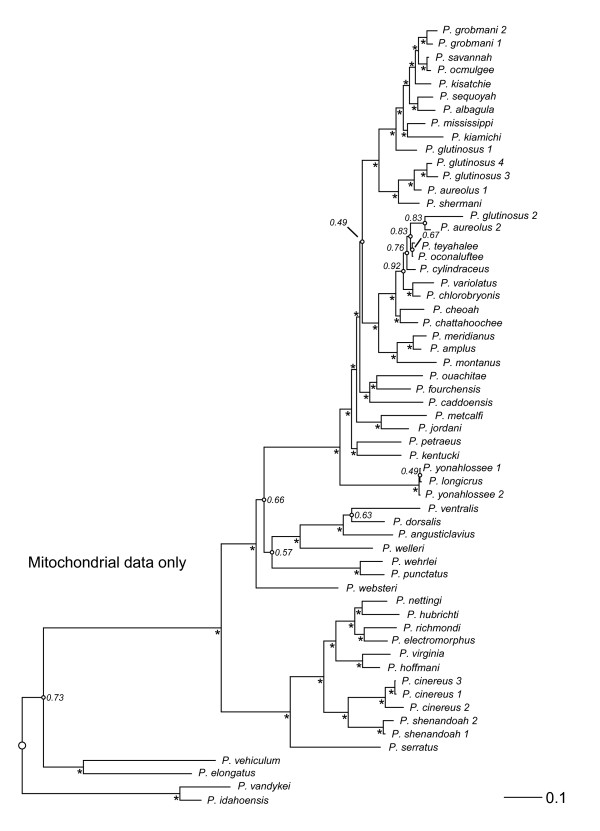
**Mitochondrial *Plethodon *phylogeny**. Phylogeny of the salamander genus *Plethodon *based on a combined, partitioned Bayesian analysis of mitochondrial DNA (mtDNA) only. An asterisk next to a node indicates strong support (Pp ≥ 0.95). Small open circles on a node indicate Pp < 0.95, and these values are listed. The outgroup taxa are excluded (but only from the figure) to facilitate presentation of branch lengths, and the root is indicated with a large open circle.

**Figure 3:**
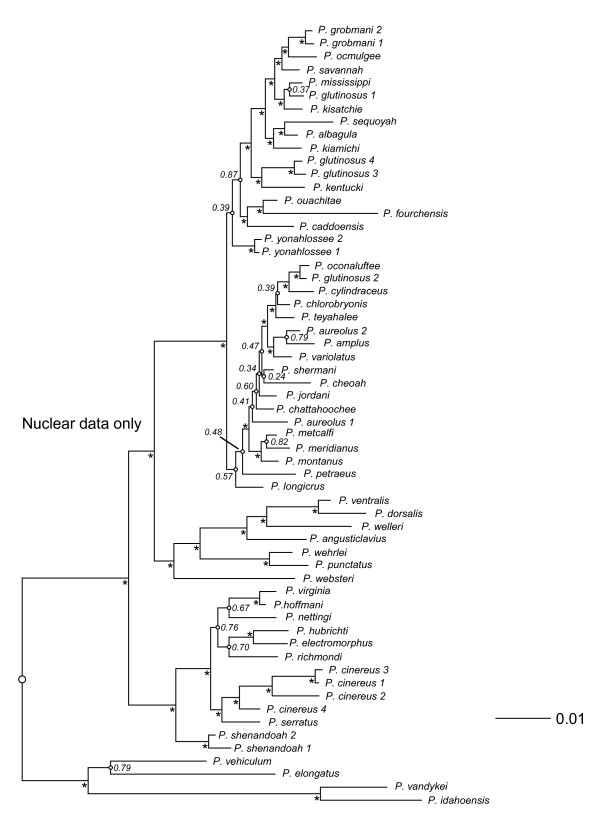
**Nuclear *Plethodon *phylogeny**. Phylogeny of the salamander genus *Plethodon *based on a combined, partitioned Bayesian analysis of nuclear DNA (nucDNA) only. An asterisk next to a node indicates strong support (Pp ≥ 0.95). Small open circles on a node indicate Pp < 0.95, and these values are listed. The outgroup taxa are excluded (but only from the figure) to facilitate presentation of branch lengths, and the root is indicated with a large open circle.

Trees from Bayesian analyses of the combined-data, mtDNA, and nucDNA for *Plethodon *are summarized in Figures 1, 2, and 3. The separate data sets generally agree on the major clades (eastern, western) and species groups (*cinereus, wehrlei-welleri, glutinosus*) recognized in previous studies [e.g., [13,52,56,57]]. Nevertheless, the mtDNA and nucDNA conflict with each other at 34 of 51 nodes, and conflicts at 19 of the 34 discordant nodes are strongly supported by both data types (Table 1). In 15 of these 19 cases, these strongly supported conflicts are resolved in favor of the mtDNA in the combined-data tree. Of the remaining four strongly supported conflicts, three (nodes 28, 36, and 45) have topologies unique to the combined-data tree, and one (node 47) is resolved in favor of the nucDNA. The topology of the combined-data tree shares 73% of its nodes with the mitochondrial tree, and 27% with the nuclear tree (Table 2). The mtDNA data set has a greater number of variable characters and a higher level of homoplasy when compared to the nucDNA (Table 3).

**Table 1:** Congruence between mtDNA, nucDNA, and combined-data trees

Clade	Total nodes	Concordant nodes	Discordant nodes	Strongly supported discordance	Weak mtDNA, strong nucDNA	Strong mtDNA, weak nucDNA	Weakly supported discordance	Strong discordance resolved favoring mtDNA
Balistid fish	23	39%	61%	14%	43%	21%	21%	50% mtDNA, *P *= 0.5000
Scarine fish	40	55%	45%	44%	22%	17%	17%	75% mtDNA, *P *= 0.1094
Hemiphractid frogs	40	45%	55%	36%	18%	23%	23%	75% mtDNA, *P = *0.1094
Hylid frogs	76	54%	46%	17%	29%	9%	46%	67% mtDNA, *P = *0.2344
*Plethodon *salamanders	51	33%	67%	56%	21%	24%	0%	79% mtDNA, ***P = *0.0074**
Phrynosomatid lizards	35	49%	51%	28%	44%	17%	11%	0% mtDNA, ***P = *0.0313**
Alcid birds	21	67%	33%	29%	14%	43%	14%	100% mtDNA, *P = *0.2500
Caprimulgid birds	55	56%	44%	17%	4%	33%	46%	50% mtDNA, *P *= 0.3750
Cotingid birds	35	63%	37%	23%	31%	0%	46%	67% mtDNA, *P = *0.3750
Dicaeid birds	28	36%	64%	17%	11%	39%	33%	33% mtDNA, *P *= 0.3750
Emydid turtles	36	33%	67%	29%	33%	17%	21%	38% mtDNA, *P = *0.2734
Cervid mammals	23	30%	70%	19%	0%	56%	25%	67% mtDNA, *P = *0.3750
Murid rodents (Philippines)	55	58%	42%	9%	43%	13%	35%	50% mtDNA, *P *= 0.5000
Murid rodents (Sahul)	60	70%	30%	39%	22%	17%	22%	0% mtDNA, ***P *= 0.0078**

Each column reports the percentage of total nodes (second column) that fall into the following categories: (a) concordant nodes are those present in the combined-data tree that are also present in both mtDNA and nucDNA trees; (b) discordant nodes are absent in one or both of the trees from the separate data sets; (c) strongly supported discordance indicates branches for which conflicting resolutions in mtDNA and nucDNA are strongly supported (Pp ≥ 0.95) by each one; (d) weak mtDNA (or nucDNA), strong nucDNA (or mtDNA) indicates branches for which conflicting resolutions were weakly supported by one (mtDNA or nucDNA) and strongly supported by the other; (e) weakly supported discordance indicates branches for which conflicting resolutions in mtDNA and nucDNA are weakly supported by both; (f) the far-right column gives the proportion of nodes with strongly supported discordance that are resolved in favor of the mtDNA in the combined-data tree. *P-*values indicate whether the number of these resolutions favoring mtDNA data are significantly different from random (exact binomial, *p *= 0.50). Significant *P*-values are bold faced.

**Table 2:** Similarity between trees from different data sets

Clade	Combined-data and mtDNA	Combined-data and nucDNA	mtDNA and nucDNA
Balistid fish	16%	**24%**	11%
Scarine fish	**83%**	63%	50%
Hemiphractid frogs	**64%**	52%	30%
Hylid frogs	27%	**44%**	13%
*Plethodon *salamanders	**73%**	27%	23%
Phrynosomatid lizards	37%	**71%**	26%
Alcid birds	**91%**	33%	23%
Caprimulgid birds	53%	**82%**	38%
Cotingid birds	53%	**71%**	35%
Dicaeid birds	**80%**	34%	24%
Emydid turtles	54%	**60%**	37%
Cervid mammals	**99%**	37%	27%
Murid rodents (Philippines)	30%	**63%**	23%
Murid rodents (Sahul)	55%	**96%**	53%

The proportion of nodes shared between each pair of trees (mtDNA, nucDNA and combined-data) for each clade. Boldfaced proportion indicates which of the two data sets (mtDNA, nucDNA) the combined-data tree is more similar to.

**Table 3:** Variability and homoplasy in each type of data

Clade	nucDNA variable characters	mtDNA variable characters	Ratio of variable characters nucDNA: mtDNA	nucDNA consistency index/retention index	mtDNA consistency index/retention index
Balistid fish	341	337	1.01	0.5851/0.7298	0.4175/0.5380
Scarine fish	612	743	0.82	0.5579/0.7874	0.3805/0.6012
Hemiphractid frogs	441	1344	0.33	0.6427/0.8552	0.3065/0.4589
Hylid frogs	715	1442	0.50	0.2844/0.5646	0.1486/0.3135
*Plethodon *salamanders	1204	1400	0.86	0.6042/0.8132	0.3069/0.6329
Phrynosomatid lizards	1155	2258	0.51	0.5498/0.7490	0.3327/0.3284
Alcid birds	255	1559	0.16	0.6471/0.7918	0.4129/0.5823
Caprimulgid birds	790	522	1.51	0.4708/0.7560	0.2216/0.5078
Cotingid birds	440	493	0.89	0.5858/0.7162	0.2300/0.3154
Dicaeid birds	86	660	0.13	0.8043/0.9455	0.7067/0.3793
Emydid turtles	477	460	1.04	0.6581/0.8662	0.4591/0.7820
Cervid mammals	127	624	0.20	0.7414/0.9085	0.3097/0.4704
Murid rodents (Philippines)	640	628	1.02	0.4179/0.6474	0.1649/0.3342
Murid rodents (Sahul)	4226	1175	3.60	0.5094/0.7129	0.1666/0.2974

A summary of the number of variable characters that each data type (mtDNA, nucDNA) contributes to each combined analysis and the amount of homoplasy in each data set (lower values indicate more homoplasy; see Additional File 8 for additional details on each data set). The consistency index excludes uninformative characters. Outgroups were not included.

The mean branch lengths and node depths grouped by clade-resolution category are summarized in Table 4, and significance tests are summarized in Additional File 1. Concordance between the nuclear and mitochondrial trees occurs on significantly longer branches in the combined-data tree (*W *= 131.5; *P = *0.0055). Discordance occurs at intermediate branch lengths, and the branches resolved favoring mtDNA are not significantly different in length from those favoring nucDNA clades (*W *= 75; *P = *0.50). Clades found only in the combined-data tree are significantly shorter than clades that are concordant between mtDNA and nucDNA (*W *= 67; *P = *0.0007) and those that are discordant (*W *= 104; *P = *0.015). Nodes of the combined-data tree favoring the mtDNA occur at shallower depths in the combined-data tree than those favoring the nucDNA, but this trend was not significant (*W *= 80; *P = *0.3454).

**Table 4:** Mean branch lengths and node depths across clade resolution categories

Clade	Type of node	Number of nodes	Mean branch length	Standard error branch lengths	Mean node depth	Standard error node depth
Balistid fish	*Concordant*	9	0.0113	0.0024	6.44	0.53
	*mtDNA wins*	3	0.0045	0.0021	6.33	0.84
	*nucDNA wins*	5	0.0042	0.0004	6.00	0.88
	*Unique*	6	0.0051	0.0005	3.00	0.73
Scarine fish	*Concordant*	22	0.0110	0.0028	5.68	0.53
	*mtDNA wins*	10	0.0042	0.0040	6.90	0.95
	*nucDNA wins*	4	0.0058	0.0008	5.75	1.00
	*Unique*	4	0.0023	0.0007	6.75	1.97
Hemiphractid frogs	*Concordant*	18	0.0528	0.0129	5.72	0.67
	*mtDNA wins*	13	0.0118	0.0019	8.85	1.36
	*nucDNA wins*	6	0.0142	0.0022	4.67	0.49
	*Unique*	3	0.0068	0.0007	8.00	1.00
Hylid frogs	*Concordant*	41	0.0828	0.0081	10.12	0.63
	*mtDNA wins*	11	0.0686	0.0049	7.45	0.86
	*nucDNA wins*	13	0.0299	0.0150	9.46	1.11
	*Unique*	11	0.0196	0.0024	9.27	1.02
*Plethodon *salamanders	*Concordant*	17	0.0199	0.0054	5.00	0.74
	*mtDNA wins*	25	0.0067	0.0020	8.32	1.93
	*nucDNA wins*	5	0.0052	0.0011	6.20	0.67
	*Unique*	4	0.0023	0.0003	8.25	1.11
Phrynosomatid lizards	*Concordant*	17	0.1046	0.0169	5.29	0.73
	*mtDNA wins*	2	0.0221	0.0126	8.00	1.31
	*nucDNA wins*	12	0.0395	0.0091	7.92	3.00
	*Unique*	4	0.0152	0.0032	9.50	2.63
Alcid birds	*Concordant*	14	0.0478	0.0093	3.71	0.40
	*mtDNA wins*	6	0.0212	0.0073	3.17	0.65
	*nucDNA wins*	1	0.0057	-	2.00	-
	*Unique*	0	-	-	-	-
Caprimulgid birds	*Concordant*	31	0.0562	0.0085	7.87	0.54
	*mtDNA wins*	10	0.0309	0.0042	8.40	0.99
	*nucDNA wins*	10	0.0149	0.0045	7.00	1.06
	*Unique*	4	0.0090	0.0023	8.00	0.82
Cotingid birds	*Concordant*	22	0.0550	0.0081	5.27	0.52
	*mtDNA wins*	5	0.0132	0.0017	5.60	1.03
	*nucDNA wins*	7	0.0140	0.0029	5.29	1.15
	*Unique*	1	0.0091	-	1.00	-
Dicaeid birds	*Concordant*	10	0.0919	0.0104	3.70	0.37
	*mtDNA wins*	13	0.0604	0.0042	5.54	1.49
	*nucDNA wins*	4	0.0312	0.0162	5.25	0.79
	*Unique*	1	0.0214	NA	5.00	NA
Emydid turtles	*Concordant*	12	0.0078	0.0013	3.50	0.47
	*mtDNA wins*	7	0.0024	0.0004	6.29	0.88
	*nucDNA wins*	12	0.0032	0.0006	6.00	0.87
	*Unique*	5	0.0007	0.0002	7.80	1.39
Cervid mammals	*Concordant*	7	0.0288	0.0030	2.57	0.43
	*mtDNA wins*	14	0.0160	0.0022	4.36	0.34
	*nucDNA wins*	2	0.0066	0.0014	4.00	3.00
	*Unique*	0	-	-	-	-
Murid rodents (Philippines)	*Concordant*	32	0.1341	0.0146	7.06	0.50
	*mtDNA wins*	7	0.1011	0.0083	6.71	0.89
	*nucDNA wins*	13	0.0494	0.0644	6.46	1.15
	*Unique*	3	0.0305	0.0065	3.67	1.20
Murid rodents (Sahul)	*Concordant*	42	0.0097	0.0013	6.64	1.03
	*mtDNA wins*	4	0.0018	0.0003	9.50	4.75
	*nucDNA wins*	13	0.0038	0.0008	6.77	1.88
	*Unique*	1	0.0015	-	7.00	-
Pooled across clades	*Concordant*	294	0.0556	0.0035	0.51	0.01
	*mtDNA wins *	130	0.0265	0.0045	0.60	0.02
	*nucDNA wins*	107	0.0198	0.0024	0.52	0.02
	*Unique*	47	0.0108	0.0015	0.53	0.04

A summary of the mean branch lengths and mean node depths of branches in the combined-data trees for each clade, grouped by how they are resolved. For the node depths, larger numbers indicate shallower nodes (i.e. those closer to the tips and farther from the root). The last row of pooled data reports mean relative branch lengths and mean relative node depths. Significance testing is summarized in the Results and more extensively in Additional File 1.

### Comparisons across clades

Trees from Bayesian analyses of the combined-data, mtDNA, and nucDNA for the other 13 vertebrate clades are summarized in Additional File 2. Combining our results from *Plethodon *with those from these 13 other clades, we find that discordance between trees from mtDNA and nucDNA is very common, with only 30-70% (mean = 49%) of nodes concordant in each study. Seven of the 14 data sets show extensive incongruence between mtDNA and nucDNA, with only a minority of nodes (range among seven data sets = 30-49%; mean = 38%; Table 1) in common between them in each data set. In addition, four of the remaining seven data sets show only a slight majority of congruent nodes between mtDNA and nucDNA (range among four data sets = 54-58%; mean = 56%; Table 1). The final three data sets show more extensive congruence (range among three data sets = 63-70%; mean = 67%; Table 1).

Nevertheless, despite this widespread incongruence, in all clades except *Plethodon*, only a minority of the conflicts between mtDNA and nucDNA are strongly supported (range among 13 clades = 9-44%; mean = 25%; *Plethodon *= 56%; Table 1). These strongly supported conflicts are often resolved in favor of mtDNA (mean = 56% across the 14 data sets; 79% in *Plethodon*), but the trend is not significant for most data sets, and in four out of 14 data sets, these strong conflicts are more often resolved in favor of nucDNA (Table 1). Of the remaining conflicts, 0-46% (mean = 26%) were weakly supported by both data sets, 0-56% (mean = 23%) were strongly supported by nucDNA, but weakly supported by mtDNA, and 0-44% (mean = 24%) were weakly supported by nucDNA, but strongly supported by mtDNA (Table 1).

Surprisingly, we find that the combined-data trees are more similar to the nucDNA trees for eight of 14 data sets (Table 2). Four of those eight data sets have nearly equal numbers of variable characters between the mtDNA and nucDNA data sets (balistid fish, cotingid birds, emydid turtles, murid rodents (Philippines); Table 3), but two actually have many more variable mtDNA characters than nucDNA characters (hylid frogs, phrynosomatid lizards; Table 3). The remaining two data sets (caprimulgid birds, murid rodents (Sahul = Australia and New Guinea); Table 3), had substantially more variable nucDNA characters than mtDNA characters.

The ability of nucDNA data to sometimes dominate more nodes of the combined-data tree with only a minority of variable characters is surprising. One obvious explanation for this pattern is that the mtDNA characters have consistently higher levels of homoplasy than nucDNA characters (Table 3). However, the proportion of shared nodes between the combined-data tree and the mtDNA tree (first column, Table 2) was not correlated with either of our indices of relative mtDNA homoplasy (consistency index: *r *= 0.33; *P *= 0.26; retention index: *r *= 0.19; *P *= 0.51). The proportion of shared nodes between the combined-data tree and the mtDNA tree was not significantly correlated with the proportion of mtDNA variable sites (*r *= 0.49; *P *= 0.08), although there is a trend in this direction. Multiple regression of the proportion of nodes shared between the mtDNA and combined-data trees on homoplasy and variability was not significant for either homoplasy index (all values of *P *≥ 0.807).

**Figure 4:**
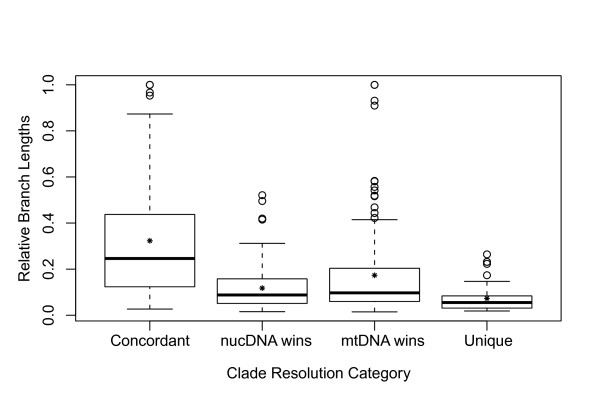
**Branch lengths by clade resolution category**. Box plots of the relative branch lengths for each clade resolution category for the 14 data sets. For each category, the median is indicated by the black bar, and the mean is indicated by the asterisk.

Comparisons across all 14 data sets confirm our prediction that branches in the combined-data tree that are concordant between mtDNA and nucDNA are longer on average than other branches (Table 4; Figure 4; *concordant *vs. *discordant *in Additional File 1). However, contrary to our expectations, there is no support for the hypothesis that shorter branches tend to be resolved in favor of mtDNA and longer branches in favor of nucDNA (see Additional File 1). The only significant pattern is found in hylid frogs (*W *= 37; *P *= 0.0475) and caprimulgid birds (*W *= 91; *P *= 0.0011), in which clades resolved in favor of mtDNA are significantly longer than those resolved in favor of nucDNA (the opposite of our expectations).

**Figure 5:**
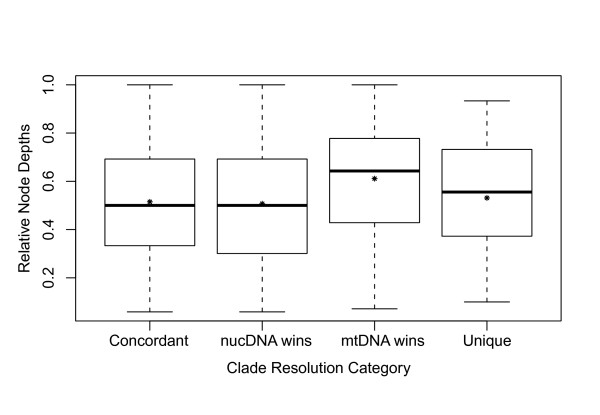
**Node depths by clade resolution category**. Box plots of the relative node depths for each clade resolution category for the 14 data sets. Larger depth indices correspond to shallower nodes. For each category, the median is indicated by the black bar, and the mean is indicated by the asterisk.

Thirteen out of 14 clades (all except hylids) show the predicted pattern in which deeper branches of the combined-data tree are resolved in favor of nucDNA and shallower branches are resolved in favor of mtDNA (Table 4; Additional File 1). Although this pattern is only significant within hemiphractids (*W *= 69; *P = *0.0055), finding the same pattern in 13 of 14 clades is statistically significant (*P *< < 0.0001; exact binomial test). The lack of significant patterns within each clade may reflect limited sample size for significance testing (e.g., phrynosomatids have only two clades resolved in favor of mtDNA). Pooling relative node depths across clades shows that branches on which mtDNA is favored are significantly shallower than branches on which nucDNA is favored (*W *= 5655.5; *P *= 0.0133; Figure 4), and nodes that are concordant between mtDNA and nucDNA are significantly deeper than discordant clades (*W *= 37282.5; *P *= 0.0261; Figure 5). Across all clades, relative node depth is negatively correlated with relative branch length (*r_s _*= -0.31; *P *< < 0.00001), such that longer branches tend to be found deeper in the tree. The longer branches deeper in the tree may explain the greater concordance between mtDNA and nucDNA on deep branches.

## Discussion

### Consequences of combining mitochondrial and nuclear data for phylogenetic analysis

Combining data from nucDNA and mtDNA is a common practice in phylogenetic studies, but one whose consequences have gone largely unstudied (or at least under-reported). This is surprising given the extensive debate about pros and cons of mtDNA data for phylogenetic analysis [e.g. [20-24,28,37]], and about combining data in general [e.g. [6,19,31-33,62]]. In this study, we test several key predictions about how mtDNA and nucDNA interact in combined-data analyses, using new data from *Plethodon *salamanders and published data from 13 other vertebrate clades.

Our results suggest that even though conflicts between mtDNA and nucDNA are widespread in these 14 groups, the general dominance of mtDNA in combined-data trees is not supported, even in two clades in which the number of variable mtDNA characters greatly outnumbers those from the nucDNA (see below). We find that discordance between mtDNA and nucDNA trees is common: across the 14 data sets, 30-70% (mean = 49%) of nodes are concordant. This suggests that the issue of how these conflicts are resolved in the combined-data analysis is of critical importance. But we also find that many of these conflicts are only weakly supported by one or both data sets. Strongly supported conflicts (for which conflicting clades are strongly supported by each type of data) tend to be uncommon (mean = 27% of discordant nodes, range 9-56%), and may be resolved in favor of either mtDNA or nucDNA with almost equal frequency (mean = 54% in favor of mtDNA, range = 0-100%).

Surprisingly, we find that in the majority of the 14 data sets, the combined-data tree is more similar to the nucDNA tree than the mtDNA tree (i.e., shares more nodes). In fact, nucDNA can dominate the combined-data tree even when the number of variable mtDNA characters is 2-3 times that of the variable nucDNA characters (i.e., in hylid frogs and phrynosomatid lizards). The most obvious explanation for this pattern is that the lower homoplasy of nucDNA characters may outweigh the influence of the larger numbers of variable mtDNA characters. However, our analyses of the relationship between homoplasy levels and the dominance of the combined-data tree by mtDNA do not support the idea that more homoplasy in mtDNA necessarily leads to combined-data trees that more closely resemble the nucDNA trees. There are several possible explanations for this unexpected combination of results. One is that the differences in homoplasy between mtDNA and nucDNA are primarily what matter, and that variation in levels of homoplasy among mtDNA data sets (which is what our indices mostly reflect, see Methods) is relatively unimportant. Another (non-exclusive) possibility is that the conflicts between mtDNA and nucDNA occur because of processes that are not reflected by levels of homoplasy in the mtDNA data (e.g., introgression, incomplete lineage sorting).

Contrary to our expectations, we find no evidence that shorter branches are generally resolved in favor of mtDNA. In fact, among the 14 data sets, the only significant trend is for longer branches to be resolved in favor of mtDNA, which occurs in hylid frogs and caprimulgid birds. We do find that within a given combined-data tree, there is a tendency for longer branches to be agreed upon by mtDNA and nucDNA. This result parallels the pattern seen among nuclear genes in some studies, where congruence between genes increases on longer branches, possibly due to fewer conflicts between gene and species trees associated with incomplete lineage sorting [e.g. [4,27]]. The causes of discordance between mtDNA and nucDNA on shorter branches are not entirely clear. Most of the conflicts (73%) we uncovered between mtDNA and nucDNA are not strongly supported by one or both data sets. Therefore, spurious resolution of weakly supported clades may be a major cause of disagreement. We also find that clades that are absent in both the separate mtDNA and nucDNA trees (*unique*) tend to be the shortest branches in the combined-data tree, suggesting that they have few supporting characters from either data set.

Finally, our prediction that deeper nodes tend to be resolved in favor of nucDNA was supported in 13 out of 14 data sets, and when data were pooled across clades. Interestingly, one clade (hylid frogs) showed the opposite pattern, with deeper nodes typically resolved in favor of mtDNA. In fact, the idea that mtDNA and nucDNA will resolve different portions of the phylogeny (shallow vs. deep; e.g., [38,39]) may be one of the major motivations for obtaining and combining these data types in the first place. Our prediction was based on the idea that long-branch attraction might be more common among deeper nodes, and that slow-evolving nucDNA might help resolve such problems. This prediction is further supported by a significant negative correlation between branch length and node depth, suggesting that longer branches are indeed found deeper in the tree (note that without considerable rate heterogeneity it would be difficult for a long branch to be shallowly placed). Our results here suggest that nucDNA does indeed help to resolve deeper branches in the phylogeny (see also [38,39]), and for this reason, nucDNA data are worth pursuing in clades for which phylogeny was previously estimated by mtDNA only.

In summary, our results suggest that combined analyses of mtDNA and nucDNA are not necessarily dominated by mtDNA, even though conflicts between mtDNA and nucDNA are indeed common. Thus, both data sets typically contribute to resolution of combined-data trees, and the addition of nucDNA data can be worthwhile. However, we do find considerable variation in these patterns among clades, which suggests the need for routine checking of incongruence between mtDNA and nucDNA and its impacts on combined analyses. For example, our results for *Plethodon *show widespread, strongly-supported incongruence between mtDNA and nucDNA that is generally resolved in favor of mtDNA (despite inclusion of nine nuclear genes). It should also be noted that we only considered data sets in which the overall taxon sampling of mtDNA and nucDNA was basically identical. Cases in which one data set is more broadly sampled might certainly alter these dynamics (e.g. nucDNA for 80 species and mtDNA for ~200 species; [63]). Furthermore, dramatic differences in sampling of genes between these genomes could obviously influence the results (e.g., whole mitochondrial genomes vs. a single nuclear gene; [29]). Nevertheless, our results provide an initial baseline for understanding how mtDNA and nucDNA may typically interact to determine the results of combined analyses.

### *Plethodon *phylogeny

Our survey of vertebrate clades shows that the results for *Plethodon *are quite unusual, in both the preponderance of widespread, strongly supported incongruence between mtDNA and nucDNA, and the consistency with which the incongruence is resolved in favor of the mtDNA. We speculate that mitochondrial introgression between young but distantly related species may be a major factor driving this pattern. For example, *P. shermani *has been previously classified as a member of the *jordani *species complex [e.g., [64]]. All members of the *jordani *complex, except *P. shermani*, are placed in clade B in the combined-data tree (Figure 1). We find *P. shermani *in clade A in the mtDNA (Figure 2) and combined-data (Figure 1) trees, where it is placed in a clade with *P. aureolus*, with which it is known to hybridize [52,54,57]. In contrast, in the nucDNA tree (Figure 3), *P. shermani *is placed in clade B with strong support. This pattern suggests the possibility that *P. shermani *belongs to clade B, but mitochondrial introgression with *P. aureolus *leads to its placement in clade A in the mtDNA and combined-data trees. Placement of this species into these two different major clades by mtDNA and nucDNA contributes to the broad-scale incongruence between these data sets.

Despite the widespread incongruence between mtDNA and nucDNA, we find some cases where the new nucDNA data do appear to improve the combined-data results. For example, in the mtDNA tree (Figure 2), *P. jordani *and *P. metcalfi *(of the *jordani *complex) are at the base of the *glutinosus *group, while the rest of the *jordani *complex (*P. amplus, P. cheoah, P. meridianus, P. montanus*) is within clade B (except for *P. shermani*, see above). In the nucDNA (Figure 3) and combined-data (Figure 1) analyses in the present study, *P. jordani *and *P. metcalfi *are placed in clade B with strong support.

Despite these potential improvements, there are still many issues to be resolved with future work on *Plethodon *systematics. Many clades in the nucDNA tree (Figure 3) are still weakly supported (despite use of nine nuclear genes), especially in the rapid, recent radiation of the *glutinosus *complex. Sequencing yet more nuclear loci may be helpful here. There also appear to be important taxonomic issues to resolve in the *glutinosus *complex, which will require sampling many populations as well as many loci. For example, individuals of *P. aureolus *and *P. glutinosus *are found in separate clades in both mtDNA and nucDNA, suggesting the presence of multiple species. Sampling the same nuclear genes used here in individuals from many localities within the range of each species may be a useful next step for better resolving both species limits and the phylogeny.

## Conclusions

Combined analyses of mtDNA and nucDNA are common, but the consequences of combining these data are largely unexplored. This trend is somewhat unsettling given that use of mtDNA is somewhat controversial, and given the possibility that mtDNA might dominate combined analyses due to larger numbers of variable characters. Our results here for 14 vertebrate clades show that even though conflicts between mtDNA and nucDNA are indeed widespread, they are typically weakly supported, and mtDNA does not dominate combined-data trees in the majority of clades. Instead, both data types often contribute to resolving the combined-data tree, with nucDNA being particularly useful for deep branches. Thus, even though nucDNA data is traditionally more difficult to obtain in animals than mtDNA (hence the large number of studies still using mtDNA alone), and typically yields fewer variable characters per base pair (Table 3), our results suggest that the added cost and effort needed to obtain and add nucDNA is not necessarily wasted in a combined analysis. However, our new results for *Plethodon *show that, even with large numbers of nuclear loci, mtDNA may still dominate a combined-data tree. Therefore, testing for the congruence of mtDNA and nucDNA and the impact of each data set on combined analyses is an essential precaution.

## Methods

### Sampling of taxa and genes

We obtained DNA from 50 of the 55 currently recognized species of *Plethodon *[41], representing all major clades and species groups previously recognized [e.g., [13,52,56,57]]. Most species were represented by a single individual, but some geographically widespread species were represented by up to four individuals. We also included seven outgroup species, representing three other plethodontine genera (*Aneides, Desmognathus*, and *Ensatina*) and one genus of spelerpines (*Eurycea*). Voucher numbers and localities are listed in Additional File 3. GenBank accession numbers are listed in Additional File 4.

We combined mtDNA and nucDNA data from previous studies of *Plethodon *phylogeny [56,57,61] with 1884 aligned base pairs (bp) of new data from five nuclear loci (572 variable characters; Table 5). First, we used the third intron of Rhodopsin (Rho), with primers developed specifically for use in *Plethodon *by K.H. Kozak (pers. comm.). We also tested many other nuclear introns from published lists for vertebrates [65-67], but found only one intron (GAPD; glyceralderhyde-3-phosphate dehydrogenase) that amplified well and was variable among *Plethodon *species. Finally, we also tested many loci (~22) from an *Ensatina *cDNA library provided by T. Devitt (pers. comm.). From this testing, we found three more introns that could be amplified in many *Plethodon *species and that were relatively variable among species. Based on BLAST searches of the sequences, these introns are associated with the nuclear genes RPL12 (60s ribosomal protein L12), ILF3 (interleukin enhancer binding factor 3) and Mlc2a (myosin light chain 2 mRNA). Primer sequences are provided in Additional File 5. The length and variability of each gene are described in Table 5.

**Table 5:** Genes used in the phylogenetic analysis of *Plethodon*.

Type of locus	Locus	Length	Variable characters	Parsimony-informative characters	Best-fitting model	Partitions	Number of taxa sampled	Data source
Nuclear introns	GAPD	659	221	92	GTR + Γ	none	48	this study
	ILF3	281	56	34	HKY	none	40	this study
	Mlc2a	257	79	39	GTR + Γ	none	55	this study
	RPL12	463	159	90	HKY + Γ	none	48	this study
	RHO	224	57	43	HKY + I	none	62	this study
	TPI	1938	569	211	GTR + Γ	intron/exon	29	[56]

Nuclear exons	RAG-1	1467	358	248	GTR + I + Γ	codon	60	[56]
	BDNF	707	67	29	GTR + Γ	codon	14	[60]
	POMC	481	98	41	GTR + Γ	codon	15	[60]

Mitochondrial genes	Cyt-*b*	649	369	313	GTR + I + Γ	codon	66	[56]
	ND4	686	409	364	GTR + I + Γ	codon	65	[56]
	ND2	1107	741	635	GTR + I + Γ	codon, tRNA-TRP	52	[55]

DNA was extracted from ethanol-preserved tissues using the Qiagen DNeasy tissue kit. Gene fragments were amplified using standard polymerase chain reaction (PCR) methods. PCR products were purified and sequenced using an ABI 3100 automated sequencer. Sequences were edited using Sequence Navigator (ver. 1.0.1, Applied Biosystems) or ContigExpress (Vector NTI build 175, Invitrogen). All sequences were initially aligned using MUSCLE [68], and manually refined using Se-Al v2.0a11 Carbon.

Prior to any combination of data from different genes, we used parsimony (implemented in PAUP*; [69]) to analyze each gene separately to identify any potential contaminant sequences. Contamination was hypothesized when two species had identical sequences for a given gene, and potential contaminants were re-sequenced. However, sequences were not excluded based on incongruence with previous taxonomy or with other genes, to avoid biasing the results. Only high quality sequences (i.e., few or no ambiguous bases), without potential contaminants, were used in the final analyses.

To these new data, we added 7035 bp of previously published sequence data from three sources (Table 5): (i) one nuclear protein-coding gene (recombination-activating gene 1; RAG-1), one nuclear intron (triose phosphate isomerase; TPI), and two protein-coding mitochondrial genes (cytochrome *b*; cyt-*b *and NADH dehydrogenase subunit 4; ND4) from Wiens et al. [57]; (ii) one mitochondrial protein-coding gene (NADH dehydrogenase subunit 2; ND2) from Kozak et al. [56]; and (iii) two nuclear protein-coding genes (proopiomelanocortin; POMC and brain-derived neurotrophic factor; BDNF) from Vieites et al. [61]. GenBank accession numbers for all previously published sequence data are provided in Additional File 6.

For all newly collected data, we used the same samples from Wiens et al. [57] and thus were able to use the same individuals to represent each species across most of the sampled mitochondrial and nuclear genes. For the other genes, we combined data from different individuals into a single terminal taxon to represent a given species. Combination of published data from different individuals generally followed Kozak et al. [13], who carefully combined data from Kozak et al. [56], Wiens et al. [57], and Vieites et al. [61].

### Phylogenetic methods

Phylogenetic analyses were conducted primarily using Bayesian methods, but major results were confirmed using maximum likelihood (see below). We performed three analyses: all mitochondrial genes together, all nuclear genes together, and a combined-data analysis of all molecular data. The best-fitting model for each of the five "new" genes was identified using comparisons of the Akaike Information Criterion in MrModelTest ver. 2.0 [70]. Given that these five genes are introns (i.e., no codons), we did not recognize partitions within these sequences. For the other genes, previous studies [e.g. [56,57,61]] identified best-fitting models and used comparisons of Bayes factors [71,72] to show that partitions based on codon positions are supported for all protein-coding loci. Models and partitions used are summarized in Table 5. Model parameters were unlinked between data sets. We did not assess different substitution models for different partitions within genes given that simulations show that overly simple models may be inappropriately selected when a small sample of characters is tested [73].

We conducted Bayesian analyses using MrBayes ver. 3.1.2 [74]. For each data set, we conducted two replicate searches, each using four chains and default priors. Analyses for each data set used 6.0 × 10^6 ^generations, sampling every 1000 generations. For each analysis, we assessed when stationarity was achieved based on plots of log-likelihoods over time and on the standard deviation of split frequencies between parallel searches. In all analyses, stationarity was achieved within the first 10% of generations, and this value was used as the cut-off for burn-in (trees from the first 10% were deleted). For each analysis, the phylogeny and branch lengths were estimated from the majority-rule consensus of the pooled post burn-in trees from the two replicate searches. Clades with posterior probabilities (Pp) ≥ 0.95 were considered strongly supported [e.g. [75-78]].

Some taxa proved difficult to amplify for a given gene despite repeated attempts and development of new primers. These taxa were coded as having missing data ("?") in combined analyses. Simulations [e.g. [79-81]] and empirical analyses [e.g. [63,80,82,83]] suggest that taxa with missing data can be accurately placed in phylogenies regardless of their number of missing data cells, especially when the total number of characters in the analysis is relatively high (and the incomplete taxa contain sufficient non-missing data). For the combined mtDNA and nucDNA sequence data (8919 characters total), each species had an average of 34.75% missing data cells, with a range among species of 0.16-72.71%. As one example, the individual with the most missing data, *P. shenandoah-2*, was placed with the other individual of *P. shenandoah *within the *cinereus *group in the combined-data analyses with strong support (Figure 1), suggesting that the most incomplete taxa were also accurately placed in our study. For the sake of completeness, we included data from some nuclear genes that were only sparsely sampled in previous studies (BDNF, POMC, TPI), and we did not pursue additional sequencing of these genes ourselves (given that these genes appeared to be relatively slow evolving). Simulations suggest that adding genes with extensive missing data should generally either increase accuracy in Bayesian analyses, or else have no effect [83]. However, we acknowledge that these sparsely sampled genes may have less ability to help resolve conflicts between mtDNA and nucDNA.

Another concern may be that missing data impact estimates of branch lengths [but see [83]]. We tested for a relationship between the % missing data in each species and their associated, terminal branch lengths in the combined-data tree using Spearman's rank correlation in R (i.e., if missing data consistently bias branch lengths in some way, these terminal branches should be significantly longer or shorter in species with more missing data). We found no significant relationship (*r_s _*= -0.15; *P *= 0.2288), suggesting that the amount of missing data had no consistent impact on estimated branch lengths.

We also ran each analysis in RAxML ver. 7.0.3 [84,85], conducting 100 heuristic maximum-likelihood searches combined with 500 "fastbootstrap" replicates. We used the same partitions as in the Bayesian analysis, but with the GTRGAMMA model for all partitions. This decision was made following the recommendation of Stamatakis [85]. Regardless of the initially specified model, the "fastbootstrap" setting in RAxML uses 25 rate categories (i.e. the GTRCAT model) to account for rate heterogeneity, instead of the usual four used to compute the final, optimal likelihood. Thus, a separate parameter for invariant sites should be unnecessary. The combined-data and mtDNA likelihood and Bayesian trees were nearly identical to each other (98% and 92% shared nodes, respectively). The nucDNA likelihood and Bayesian trees were less similar, but still generally concordant (78% shared nodes) and discordance was restricted to nodes with weak support (e.g., bootstrap values < 70%; [40]). Given the general similarity between Bayesian and likelihood results, we emphasize only the Bayesian results for simplicity.

### Analyses of support and congruence among *Plethodon *data sets

We used these data to test the predictions that: (i) discordance between mtDNA and nucDNA will be uncommon and weakly supported by one or both data sets, (ii) mtDNA will dominate combined-data trees given larger numbers of variable characters, (iii) nucDNA will dominate combined-data trees due to lower homoplasy, (iv) mtDNA and nucDNA will be more concordant on longer branches, and (v) nucDNA will dominate resolution of the combined-data tree on deeper and longer branches. Prior to conducting these analyses, outgroup taxa were pruned from all trees, as was *P. cinereus-4*, which lacked mtDNA data (otherwise, all taxa were represented in both mtDNA and nucDNA trees). All statistical analyses were conducted in R (ver. 2.11.1). Given that for all comparisons either one or both variables were not normally distributed (based on a Shapiro-Wilk test), all tests used were non-parametric unless otherwise noted.

We used the proportion of nodes shared between each pair of trees (mtDNA + nucDNA, combined-data + mtDNA, and combined-data + nucDNA) as our index of similarity between trees, based on Rohlf's [86] consensus index (implemented in PAUP*). We also tallied the Bayesian support (posterior probability; Pp) for each concordant or discordant clade (see below).

We determined if a given clade in the combined-data tree was concordant or discordant with trees from separate analyses of mtDNA and nucDNA data. We also calculated the support value (Pp) for the concordant or discordant clades. Each clade in the combined-data tree was assigned a number (Figure 1) and its Bayesian support (Pp) was recorded. If the same clade appeared in the separate mtDNA or nucDNA trees, it was listed as supported by that data set with a given Pp. If a clade in the combined-data tree was not present in either the mtDNA or nucDNA trees, it was considered discordant with that data set. The support value for these discordant clades was the highest Pp for any clade inconsistent with the monophyly of that combined-data clade. We then tallied the total number of shared nodes, total number of conflicting nodes, and, among those nodes in conflict, which were strongly supported (Pp ≥ 0.95). We also recorded which data set (mtDNA or nucDNA) the strongly supported conflicts were resolved in favor of in the combined-data tree. The number of variable characters in each data set was estimated with PAUP*. The degree of homoplasy in each data set (mtDNA, nucDNA) was calculated using the consistency index (excluding uninformative characters) and the retention index (both implemented in PAUP*), with lower values for these indices indicating higher homoplasy. These values were calculated on the combined-data Bayesian tree. We recognize that these are parsimony-based estimates of homoplasy, but they nevertheless should capture variation in homoplasy relevant to all methods. While we acknowledge that model-based measures of homoplasy are potentially available, we are not aware of such a method that would allow us to readily estimate homoplasy for entire data sets of hundreds of characters.

Next, we assessed how concordance between mtDNA and nucDNA in the combined-data analysis is related to branch lengths. We assigned each branch in the combined-data tree to one of four categories: *concordant*, *mtDNA wins*, *nucDNA wins*, and *unique*. Clades in the combined-data tree congruent with separate analyses of both mtDNA and nucDNA were categorized as *concordant*. Clades in the combined-data tree congruent with the mtDNA tree but not the nucDNA tree were categorized as *mtDNA wins*. Clades in the combined-data tree congruent with the nucDNA tree but not the mtDNA tree were categorized as *nucDNA wins*. Finally, clades in the combined-data tree not congruent with either the mtDNA or nucDNA trees were categorized as *unique*. Branch lengths from the combined-data tree were used to determine the mean branch length for each category, and the difference between the means of each of the different categories was tested for significance using an exact Wilcoxon rank-sum two-sample test (equivalent to a Mann-Whitney U test). We chose to use "wilcox.exact" (package: exactRankTests) over "wilcox.test" because many of our comparisons contained ties, and the exact test calculates an exact *P*-value in the presence of ties.

We assumed that the branch lengths from the individual data sets and the combined-data tree generally reflect the true underlying branch lengths of the species tree. We confirmed that there is a significant correlation between the lengths of branches for clades shared by the mtDNA and nucDNA trees using Spearman's rank correlation (*r_s _*= 0.53; *P = *0.03), and between the lengths of the shared branches in the mtDNA and combined trees (*r*_s _= 0.96; *P *< 0.00001) and the nucDNA and combined trees (*r*_s _= 0.73; *P *< 0.0001). We found similar results across the other 13 clades (see below) and present these results in Additional File 7.

Finally, we assessed if the combined-data tree tended to be resolved in favor of mtDNA or nucDNA at particular depths. We compared mean depth of clades between the two clade categories, *mtDNA wins *and *nucDNA wins*. We predicted that conflicts deeper in the combined-data tree would be resolved in favor of nucDNA, whereas conflicts at shallow depths would be resolved in favor of mtDNA. Clade depth was initially estimated in two ways. First, we assessed the number of nodes separating each clade from the root of the trees (e.g., clade 6 in Figure 1 is three nodes away from the root). Second, we summed the branch lengths (from the combined-data tree) along the shortest path from the root to the ancestor of the clade to estimate the path length. For both methods, smaller numbers are closer to the root and thus deeper, whereas larger numbers are closer to the tips, and thus more shallow. These two methods produced strongly correlated estimates of node depth (*r*_s _= 0.74; P < 0.000001), and in all subsequent analyses on additional data sets (see below) the first method was used to compare mean depths across categories, and is referred to as the node depth index. The difference between the means of each of the different categories was tested for significance using an exact Wilcoxon rank-sum two-sample test as described above.

### Other vertebrate clades

We tested the generality of the results from *Plethodon *by conducting identical analyses on 13 other vertebrate clades: balistid fish [87], scarine fish [88], hemiphractid frogs [89], hylid frogs [63], phrynosomatid lizards [90], alcid birds [91], caprimulgid birds [92], cotingid birds [93], dicaeid birds [94], emydid turtles [27], cervid mammals [95], and murid rodents from both the Philippines [96] and Sahul (Australia and New Guinea) [97]. These clades were selected in order to represent the major groups of vertebrates and because they have relatively large, matched mtDNA and nucDNA data sets (see Additional File 8 for data on sampling of genes and taxa, and original papers for other details). We acknowledge that these 14 clades are not a comprehensive sample of all vertebrates with published mtDNA and nucDNA data. However, each clade required extensive analyses and re-analyses (see below), and 14 clades should be adequate to detect strong general trends, if they exist (such as dominance of combined-data trees by mtDNA).

For most clades, we ran (or re-ran) Bayesian analyses to produce comparable combined-data, mtDNA, and nucDNA trees, using the same methods described for *Plethodon*. However, for emydids and phrynosomatids we used the original Bayesian results. For phrynosomatids we used results from the reduced set of 37 taxa (including *Urosaurus bicarinatus*), which have comparable data for most genes [90]. For hylids, we used the smaller set of ~80 relatively complete taxa [63]. New analyses were run for 3 to 20 million generations, depending on the number of taxa in the data set. Any taxa in these additional data sets that were missing all of one type of data (e.g., missing all mtDNA) were removed prior to the analyses. These and other minor changes from the original methods are noted in Additional Files 8 and 9. In theory, we could have done these analyses using maximum likelihood also (or instead), but many of these data sets were initially analyzed using Bayesian methods, and previous analyses of these clades and our own experience strongly suggested that likelihood analyses would yield very similar results.

The resulting trees were subjected to the same analyses described above for *Plethodon*. In addition, we explicitly tested if mtDNA dominates combined-data trees due to a larger proportion of variable characters (prediction ii above), and if nucDNA dominates combined-data trees due to lower homoplasy (prediction iii above). For (ii), we used the proportion of the total variable sites that are derived from mtDNA data, and for (iii), we used an index of relative homoplasy (nucDNA homoplasy - mtDNA homoplasy; using both the consistency and retention indices). We correlated indices of these values with the proportion of nodes shared between the combined-data and mtDNA trees (Rohlf's consensus index values) using Pearson's product-moment correlation (note that for this analysis, all variables were normally distributed). We also used multiple regression (R package: stats; function: "lm") to test for an interaction between homoplasy and variability of data sets that may predict the proportion of nodes being shared between combined-data and mtDNA trees, once with the consistency index as our measure of homoplasy, and once with the retention index as our measure of homoplasy.

Three additional analyses of the influence of node depth were also conducted across clades. First, we tested if the overall number of sampled study clades that followed the predicted pattern (nucDNA resolves deeper nodes, mtDNA resolves shallower nodes) was significantly different from random using an exact binomial test (recommended for *n *≤ 25; [98]). In our case, the three potential outcomes were assigned equal probability and then lumped into two categories. The first category is those outcomes agreeing with our hypothesis: (a) nucDNA is favored deeper in the combined-data tree (smaller depth index) than mtDNA (shallower: larger depth index). The second category is those outcomes not agreeing with our hypothesis: (b) nucDNA and mtDNA are equally favored at a given depth (equal depth index) in the combined-data tree; or (c) mtDNA is favored deeper in the combined-data tree than nucDNA.

Second, because sample sizes within each of the ten clades were sometimes small (e.g., due to a limited number of cases in which nucDNA "wins"), we pooled data across all clades. First, all node depths were standardized by dividing them by the shallowest node (largest number) in their tree to get relative node depths for each data set. For example, in the *Plethodon *combined-data tree (Figure 1), node 5 is two nodes away from the root, while the shallowest node, 39, is 14 nodes away from the root, and so relative depth for node 5, is 2/14 = 0.1429. These relative node depths for each category (*mtDNA wins*, *nucDNA wins*) were pooled across clades, and the difference between the means of the two categories was tested for significance using an exact Wilcoxon rank-sum two-sample test as described above.

Finally, we tested for a relationship between node depth and branch length (given the possibility that greater congruence on deeper branches might be explained by deeper branches being longer). We tested for association between the standardized relative node depths for all nodes across all 14 clades and the corresponding standardized relative branch lengths using Spearman's rank correlation. Relative branch lengths were calculated similarly to relative node depths as described above. A clade's branch length was divided by the longest branch in the combined-data tree. For example, in *Plethodon*, the longest branch in the combined-data tree (Figure 1) is for node 2 at 0.0836. For Node 5, the absolute branch length is 0.0296, and its relative branch length is therefore 0.0296/0.0836, or 0.3541.

## List of abbreviations

mtDNA: mitochondrial DNA; nucDNA: nuclear DNA.

## Authors' contributions

MCFR carried out all data collection, all analyses, and drafted the manuscript. JJW conceived of the study, provided materials, and drafted the manuscript. Both authors read and approved of the final manuscript.

## Supplementary Material

Additional file 1 **Statistical analyses of congruence**. Results of statistical analyses comparing how congruence between mtDNA and nucDNA (and the resolution of discordance between them in the combined analyses) is related to the length and depth of branches in the combined-data tree. Significant *P*-values are boldfaced, indicating that the mean branch lengths being compared are significantly different from each other. PDF file. Click here for file

Additional file 2 **Phylogenies for each vertebrate clade**. Supplemental figures S1 through S39. Phylogenies for each sampled vertebrate clade based on a partitioned Bayesian analysis of combined data (first tree), mitochondrial DNA (second tree), and nuclear DNA (third tree). An asterisk next to a node indicates strong support, (Pp) ≥ 0.95. Small white circles on a node indicate (Pp) < 0.95 and these values are listed. Integers next to each node in the combined tree correspond to clade numbers used in analyses. The outgroup taxa are excluded for all groups to facilitate presentation of branch lengths, and the root is indicated with an open circle. Figures S1, S2, S3: balistid fish; Figures S4, S5, S6: scarine fish; Figures S7, S8, S9: hemiphractid frogs; Figures S10, S11, S12: hylid frogs; Figures S13, S14, S15: phrynosomatid lizards; Figures S16, S17, S18: alcid birds; Figures S19, S20, S21: caprimulgid birds; Figures S22, S23, S24: cotingid birds; Figures S25, S26, S27: dicaeid birds; Figures S28, S29, S30: emydid turtles; Figures S31, S32, S33: cervid mammals; Figures S34, S35, S36: murid rodents (Philippines); Figures S37, S38, S39: murid rodents (Sahul = Australia-New Guinea). PDF file. Click here for file

Additional file 3 ***Plethodon *specimens used in this study**. New data for this study were collected from the following specimens of *Plethodon *and outgroups from the listed localities. Whenever possible, existing data were matched by individual to the new data. Numbers following species names correspond to specimen numbers used in the figures. Acronyms for voucher specimens are as follows: AC = Andy Coleman field series; APPSU = Appalachian State University collection; DBS = Don B. Shepard field series; DWW = David W. Weisrock field series; JB = Joseph Bernardo field series, JJW = John J. Wiens field series; RH = Richard Highton field series; RMB = Ronald M. Bonett specimen number; RWV = R. Wayne VanDevender field series; SDF = San Diego Natural History Museum field series; UTA A = University of Texas at Arlington amphibian collection; UABC = Universidad Autonoma de Baja California. PDF file. Click here for file

Additional file 4 **GenBank accession numbers for new data collected for this study**. Sequences that are less than 200 bp (denoted by *****) are not accepted by GenBank and are available from M.C. Fisher-Reid upon request. Dashes (-) indicate that the sequence was not collected for that individual at that locus. Click here for file

Additional file 5 **Primer sequences for new nuclear genes**. Primers for five nuclear genes (RHO, RPL12, Mlc2a, ILF3, GAPD) from which new sequence data for *Plethodon *were collected for this study. Forward primers are indicated by "F" in the primer name, and reverse primers are indicated by "R" in the primer name. PDF file. Click here for file

Additional file 6 **GenBank accession numbers for previously published data used in this study**. Sources include: RAG-1, TPI, ND4 and Cyt-*b *data from Wiens et al. 2006. POMC and BDNF data from Vieites et al. 2007; Bonnet et al. 2009. ND2 data from Kozak et al. 2005; Weisrock et al. 2005; Kozak et al. 2006a; Kozak et al. 2006b. PDF file. Click here for file

Additional File 7 **Branch length correlations among data types for each vertebrate clade**. For each clade, all branches shared between a pair of trees (combined + mtDNA, combined + nucDNA, mtDNA + nucDNA) were tested for correlation. Nearly all comparisons show significant positive correlations in all possible combinations between the lengths of shared branches among trees. The two clades that do not show significant correlations in all combinations (dicaeid birds and cervid mammals) have very small sample sizes of shared branches, making detection of significant patterns difficult. Click here for file

Additional file 8 **Summary of data for 13 vertebrate clades**. Supplementary Tables S1-S13. Summary of data for 13 vertebrate clades, including taxon sampling, length of gene, number of variable characters, number of parsimony informative characters, the best-fitting model of evolution, and the best-fitting partitions for each gene region. PDF file. Click here for file

Additional file 9 **MrBayes settings for additional data sets**. All data followed the phylogenetic methods used for *Plethodon *except for total number of generations. The generations used for each data set that was reanalyzed for this study are listed below. Emydid turtles and phrynosomatid lizards were not reanalyzed because we had access to the MrBayes output files from the original studies. PDF file. Click here for file
