# Supplementary material for: What are the consequences of combining nuclear and mitochondrial data for phylogenetic analysis? Lessons from Plethodon salamanders and 13 other vertebrate clades
Source: BMC Evol Biol. 2011 Oct 13;11:300. doi: 10.1186/1471-2148-11-300 (PMC3203092; doi:10.1186/1471-2148-11-300)
Supplement: Additional file 1 — Statistical analyses of congruence. Results of statistical analyses comparing how congruence between mtDNA and nucDNA (and the resolution of discordance between them in the combined analyses) is related to the length and depth of branches in the combined-data tree. Significant P-values are boldfaced, indicating that the mean branch lengths being compared are significantly different from each other. PDF file. [file 1471-2148-11-300-S1.PDF]

Additional file 1 – Statistical analyses of congruence

Results of statistical analyses comparing how congruence between mtDNA and nucDNA (and the resolution of discordance between them in the combined analyses) is related to the length and depth of branches in the combined-data tree. Significant *P*-values are boldfaced, indicating that the mean branch lengths being compared are significantly different from each other.

	Clade Comparison	Balistid fish		Scarine fish		Hemiphractid frogs		Hylid frogs		*Plethodon* salamanders	
		W	*P*	*W*	*P*	*W*	*P*	*W*	*P*	*W*	*P*
Branch Lengths	*Concordant* vs. *Discordant*	58	**0.03595**	231	**0.01134**	35.5	**8.80E-06**	743	**0.0005**	131.5	**0.0055**
	mtDNA wins vs. *nucDNA wins*	5	0.5714	29	0.2218	46	0.5789	**37**	**0.0475**	75	0.5
	Unique vs. *Concordant*	43	0.06194	78.5	**0.0099**	54	**0.0015**	435	2.94E-08	67	**0.0007**
	Unique vs. *Discordant*	13	0.1702	41.5	0.166	43	0.1909	215	0.002392	104	**0.015**
Node Depth	*mtDNA wins* vs. *nucDNA wins*	6.5	0.875	27.5	0.3097	69	**0.0055**	91.5	0.2554	80	0.3454
		Phrynosomatid lizards		Alcid birds		Caprimulgid birds		Cotingid birds		Dicaeid birds	

		W	*P*	*W*	*P*	*W*	*P*	*W*	*P*	*W*	*P*
Branch Lengths	*Concordant* vs. *Discordant*	212	**7.78E-05**	76	**0.0461**	507	**7.24E-05**	244	**9.29E-06**	34	**0.00934**
	mtDNA wins vs. *nucDNA wins*	13	0.923	5	0.5714	91	**0.0011**	13	0.5025	26	1
	Unique vs. *Concordant*	68	**0.0003**	-	-	124	**3.82E-05**	21	0.1739	10	0.1818
	Unique vs. *Discordant*	42.5	0.1314	-	-	68	**0.02917**	11	0.3077	14	0.4444
Node Depth	*mtDNA wins* vs. *nucDNA wins*	12	1	4.5	0.7143	65	0.2664	17	0.9697	29.5	0.6765

		Emydid turtles		Cervid Mammals		Murid Rodents (Philippines)		Murid Rodents (Sahul)	
		W	*P*	*W*	*P*	*W*	*P*	*W*	*P*
Branch Lengths	*Concordant* vs. *Discordant*	32	**0.0005**	12	**0.002**	515	**0.0001415**	570	**0.0002**
	mtDNA wins vs. *nucDNA wins*	47.5	0.6652	25	0.1	49	0.8168	36.5	0.2496

	Unique vs. *Concordant*	60	**0.0003**	-	-	87	**0.0162**	39.5	0.1628
	Unique vs. *Discordant*	87	**0.002541**	-	**-**	41	0.3546	12	0.6667
Node Depth	*mtDNA wins* vs. *nucDNA wins*	39	0.815	14	1	41.5	0.7693	9	0.0584
