# Supplementary material for: What are the consequences of combining nuclear and mitochondrial data for phylogenetic analysis? Lessons from Plethodon salamanders and 13 other vertebrate clades
Source: BMC Evol Biol. 2011 Oct 13;11:300. doi: 10.1186/1471-2148-11-300 (PMC3203092; doi:10.1186/1471-2148-11-300)

Additional file 2 – Phylogenies for each vertebrate clade.

Supplemental figures S1 through S39. Phylogenies for each sampled vertebrate clade based on a partitioned Bayesian analysis of combined data (first tree), mitochondrial DNA (second tree), and nuclear DNA (third tree). An asterisk next to a node indicates strong support, (Pp) ≥ 0.95. Small white circles on a node indicate (Pp) < 0.95 and these values are listed. Integers next to each node in the combined tree correspond to clade numbers used in analyses. The outgroup taxa are excluded for all groups to facilitate presentation of branch lengths, and the root is indicated with an open circle. Figures S1, S2, S3: balistid fish; Figures S4, S5, S6: scarine fish; Figures S7, S8, S9: hemiphractid frogs; Figures S10, S11, S12: hylid frogs; Figures S13, S14, S15: phrynosomatid lizards; Figures S16, S17, S18: alcid birds; Figures S19, S20, S21: caprimulgid birds; Figures S22, S23, S24: cotingid birds; Figures S25, S26, S27: dicaeid birds; Figures S28, S29, S30: emydid turtles; Figures S31, S32, S33: cervid mammals; Figures S34, S35, S36: murid rodents (Philippines); Figures S37, S38, S39: murid rodents (Sahul = Australia-New Guinea). PDF file.

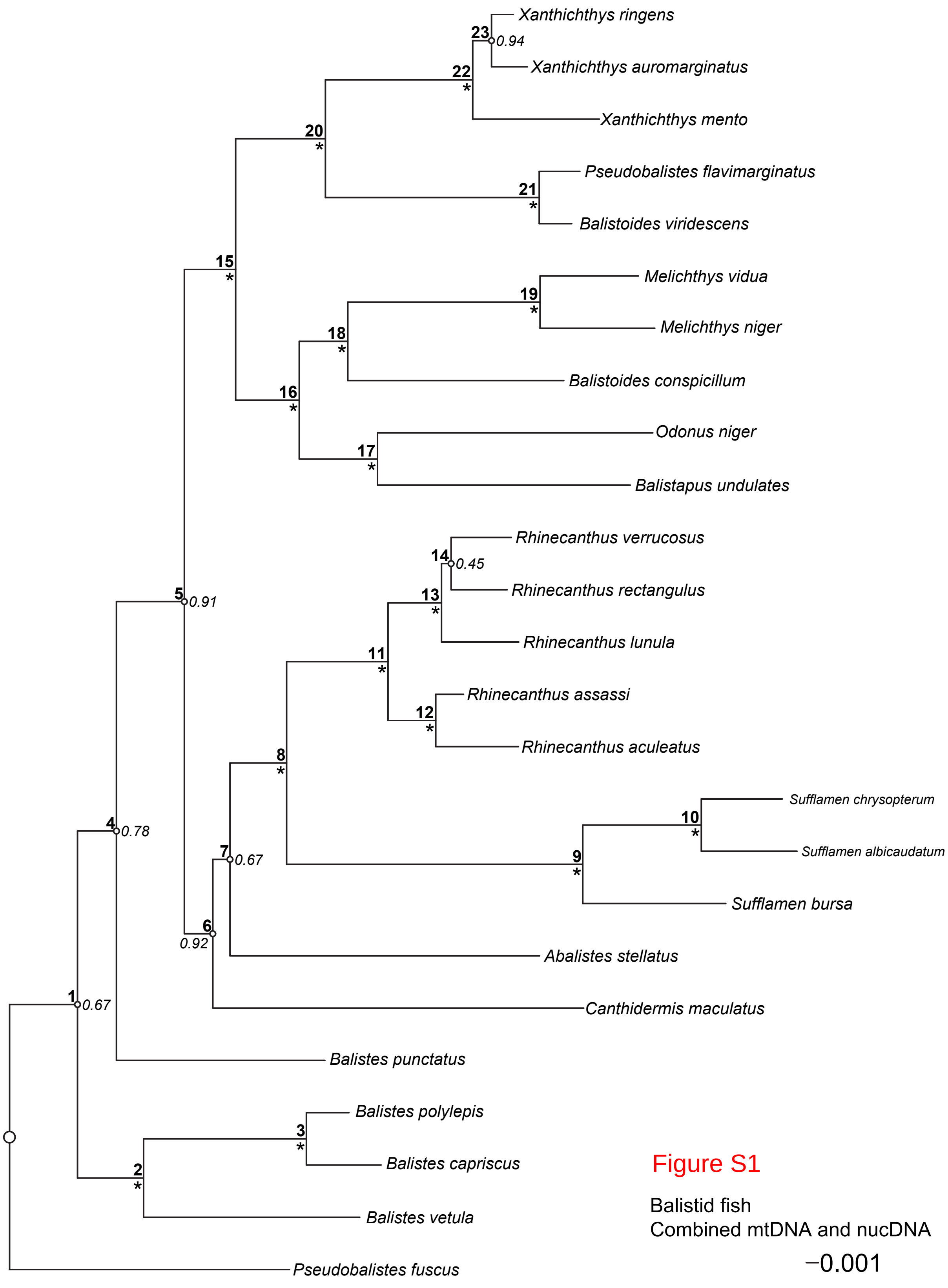

Figure S1

Balistid fish
Combined mtDNA and nucDNA

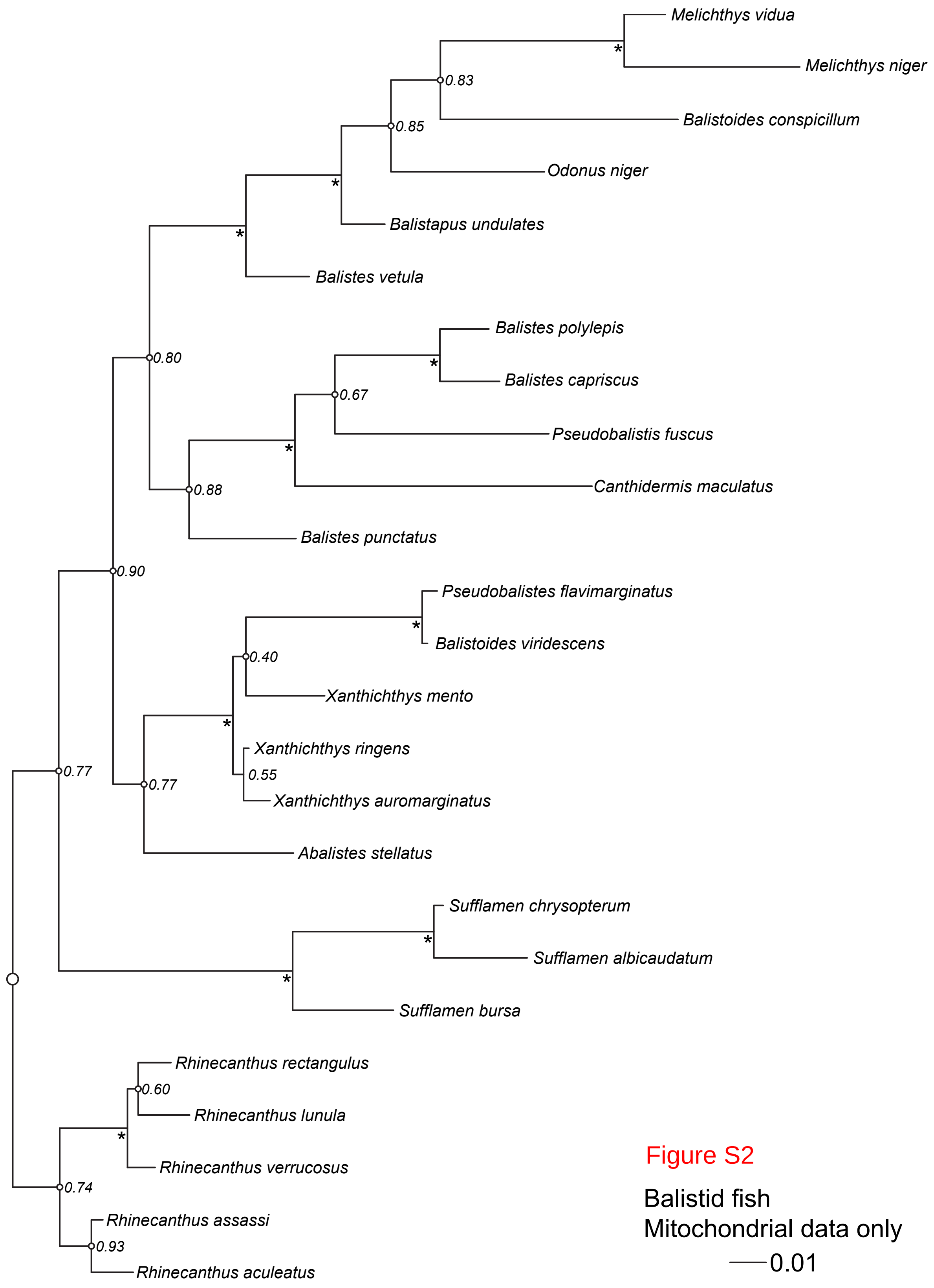

Figure S2

Balistid fish
Mitochondrial data only

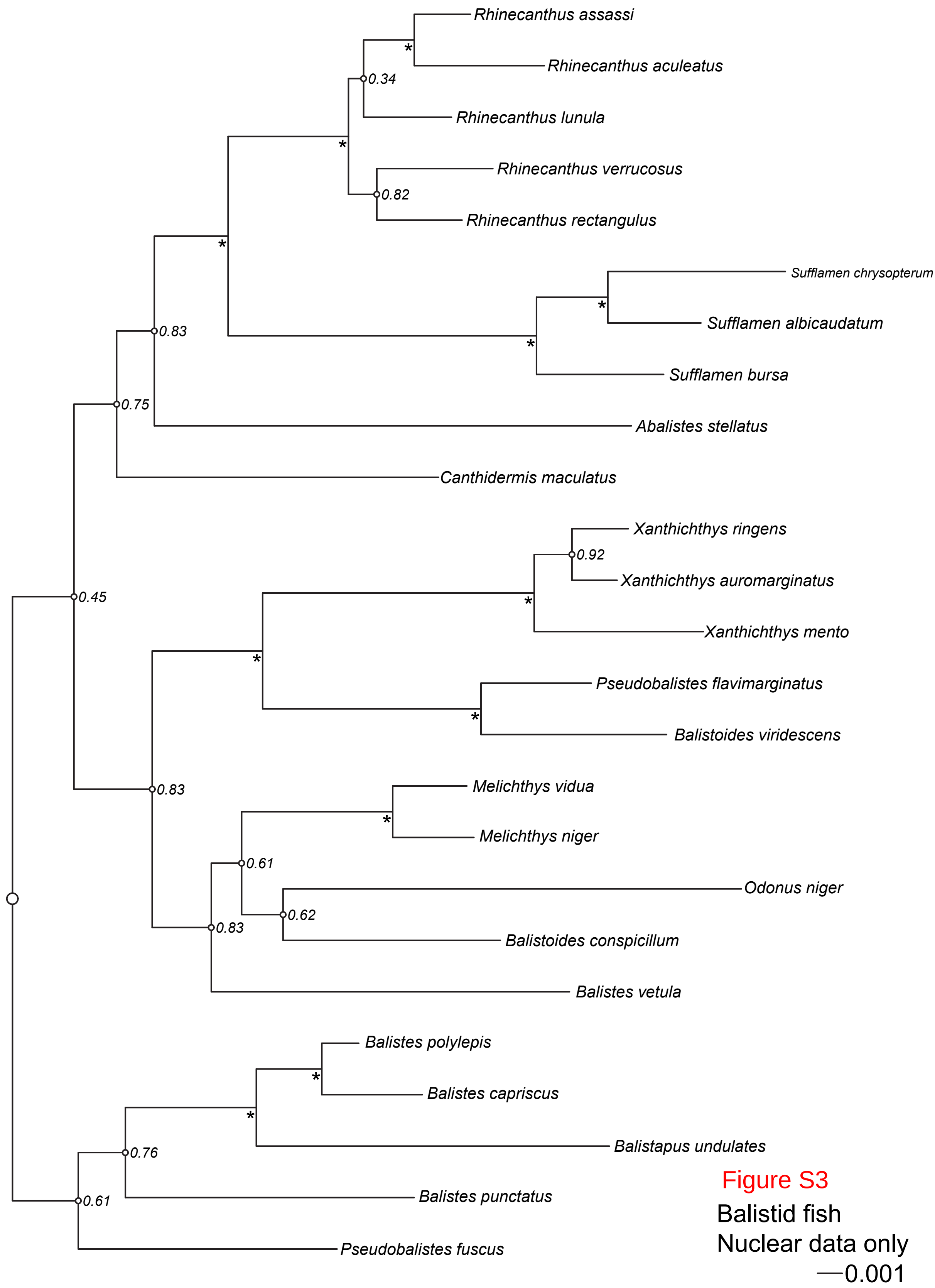

Figure S3

Balistid fish

Nuclear data only

Scarine fish
Combined mtDNA and nucDNA
Figure S4

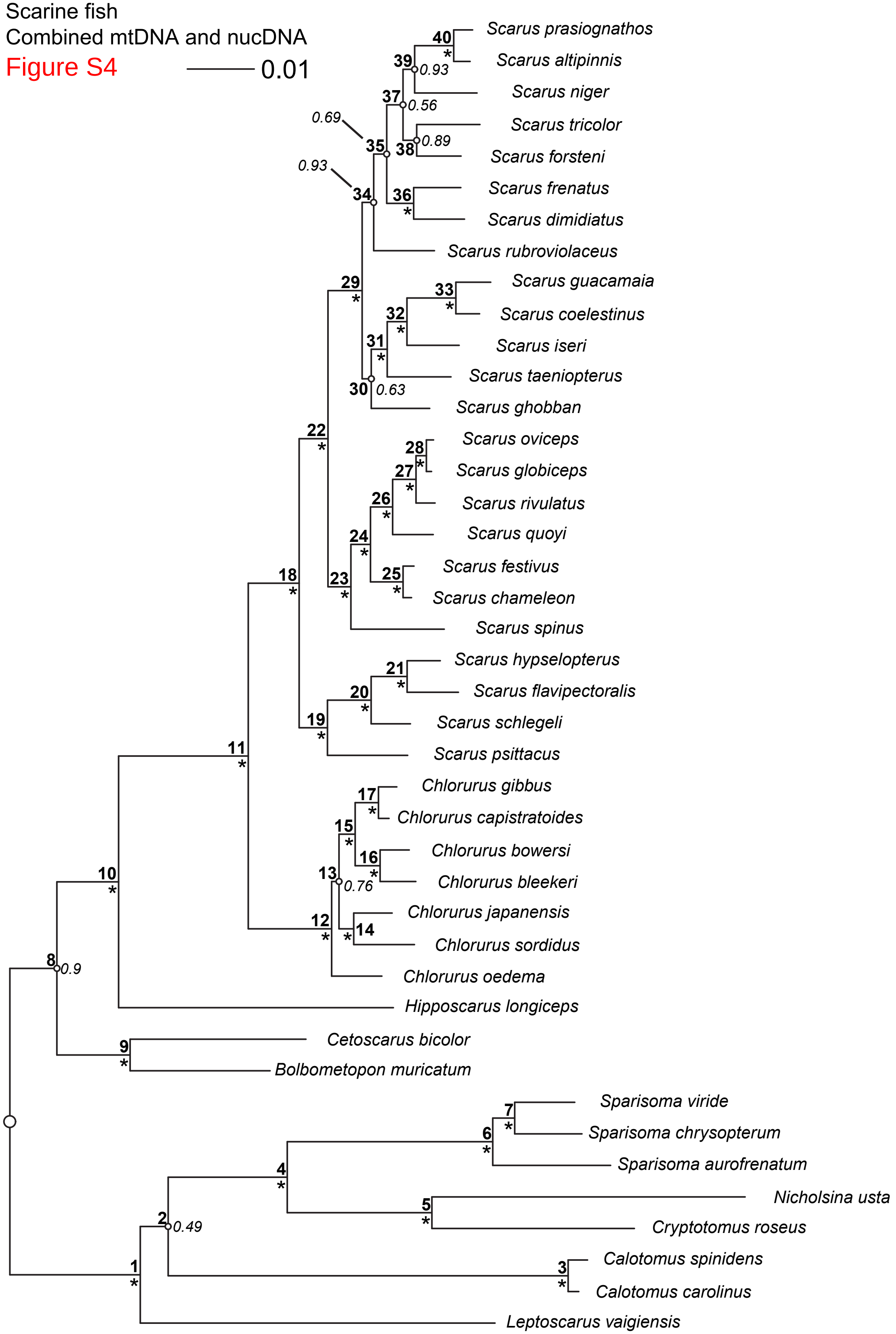

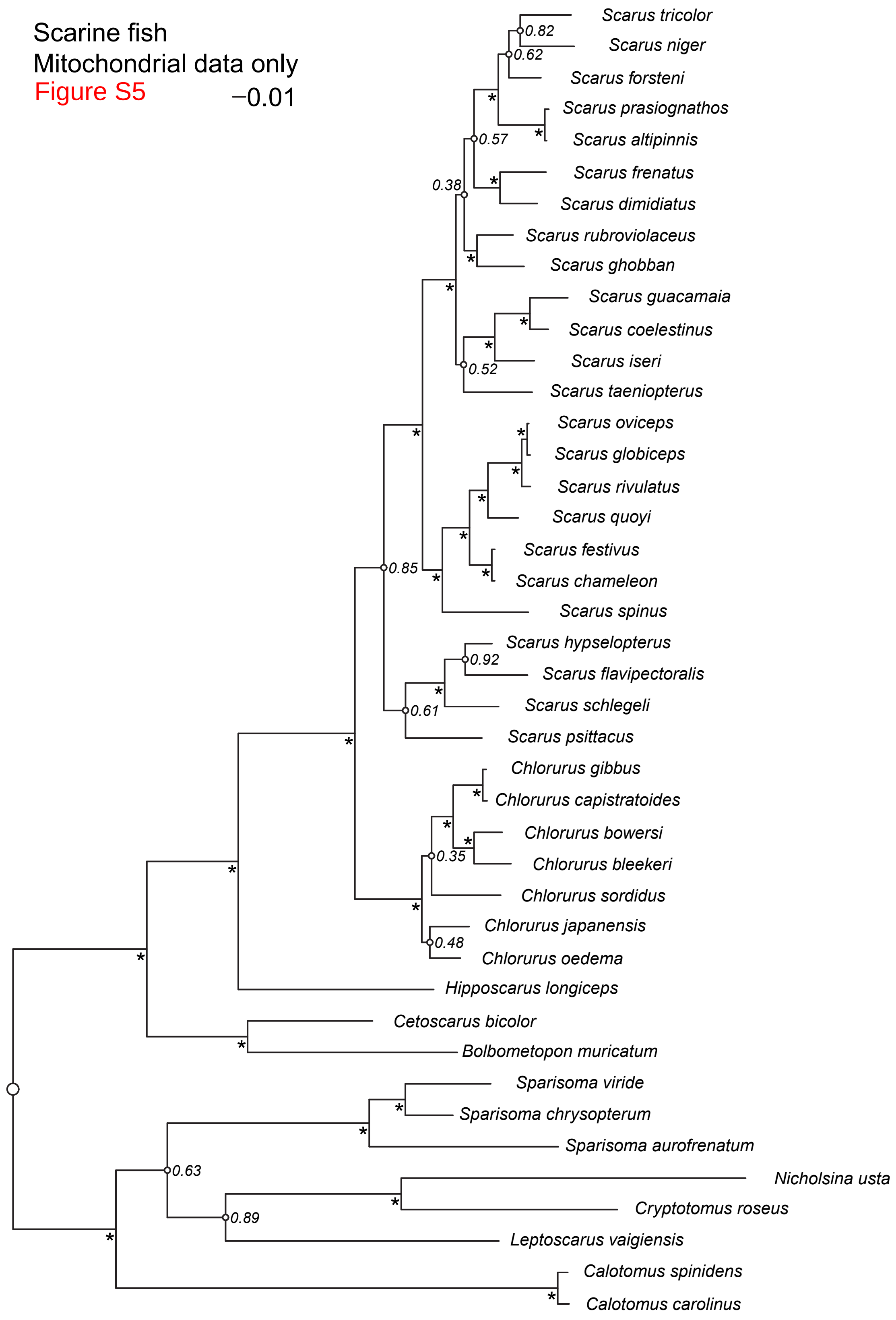

Scarine fish
Mitochondrial data only
Figure S5

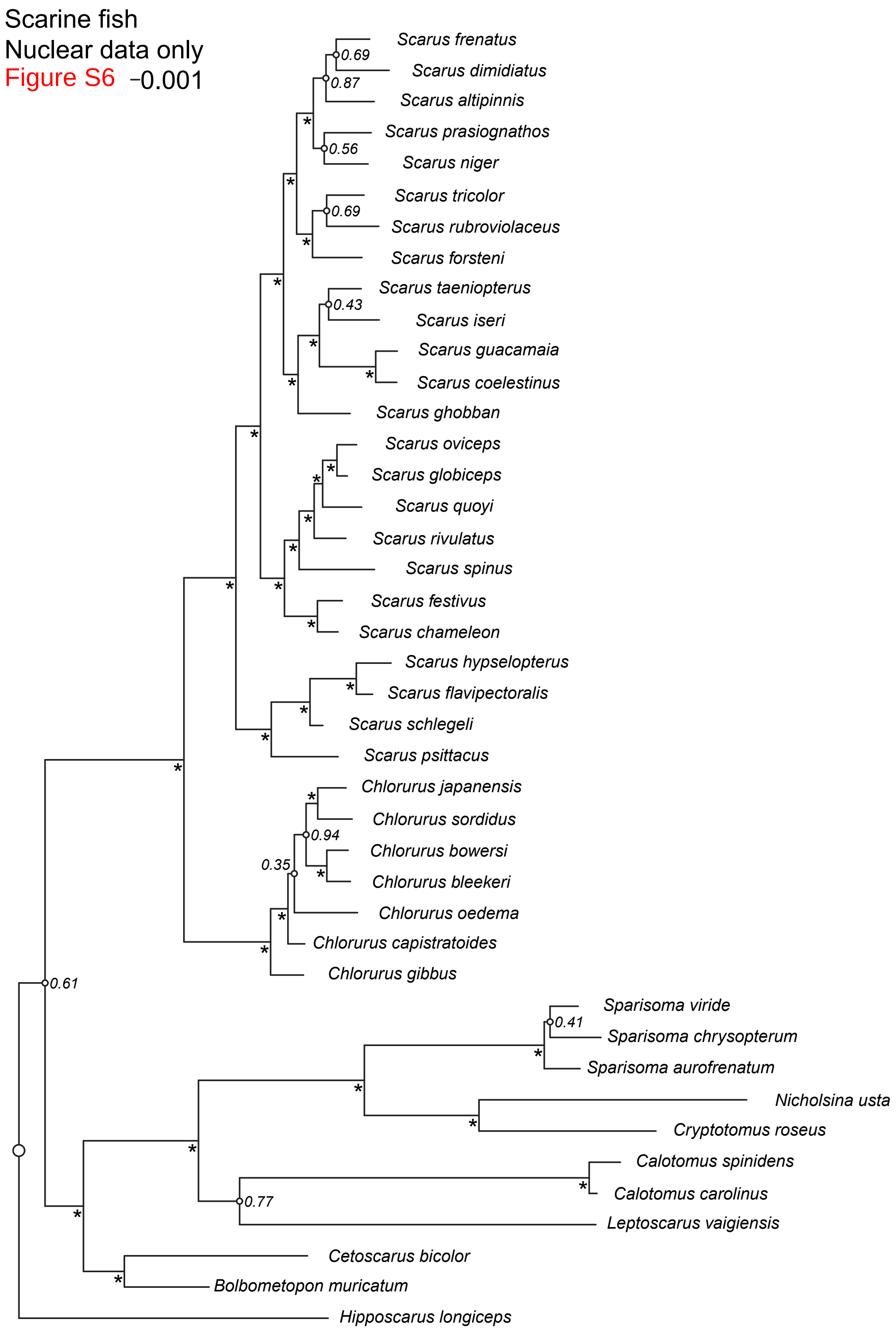
Scarine fish
Nuclear data only
Figure S6
0.001
Scarus frenatus
0.69
Scarus dimidiatus
0.87
Scarus altipinnis
Scarus prasiognathos
0.56
Scarus niger
Scarus tricolor
0.69
Scarus rubroviolaceus
Scarus forsteni
Scarus taeniopterus
0.43
Scarus iseri
Scarus guacamaia
Scarus coelestinus
Scarus ghobban
Scarus oviceps
Scarus globiceps
Scarus quoyi
Scarus rivulatus
Scarus spinus
Scarus festivus
Scarus chameleon
Scarus hypselopterus
Scarus flavipectoralis
Scarus schlegeli
Scarus psittacus
Chlorurus japanensis
Chlorurus sordidus
0.94
Chlorurus bowersi
0.35
Chlorurus bleekeri
Chlorurus oedema
Chlorurus capistratoides
Chlorurus gibbus
0.61
Sparisoma viride
0.41
Sparisoma chrysopterum
Sparisoma aurofrenatum
Nicholsina usta
Cryptotomus roseus
Calotomus spinidens
Calotomus carolinus
0.77
Leptoscarus vaigiensis
Cetoscarus bicolor
Bolbometopon muricatum
Hipposcarus longiceps

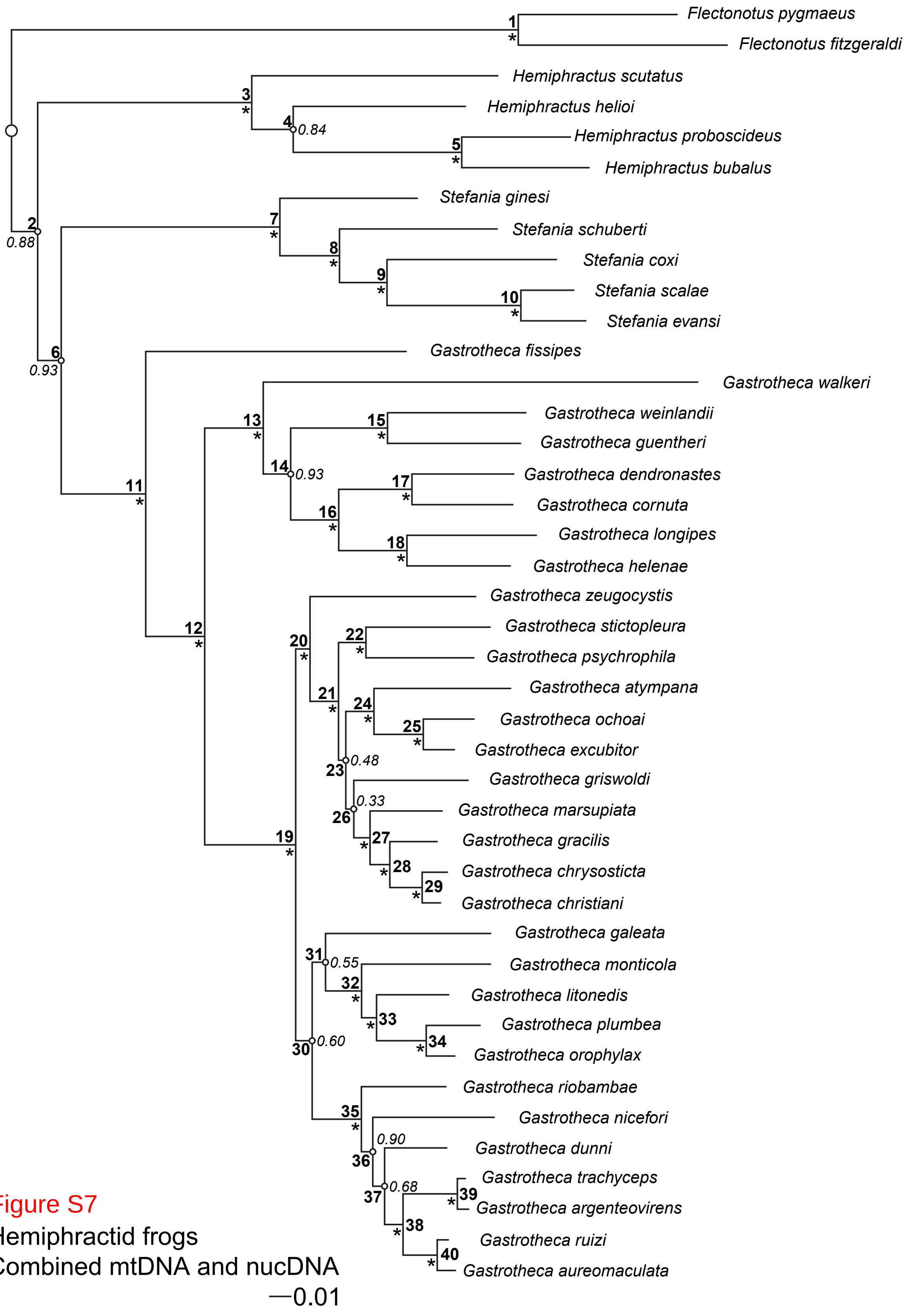

Figure S7
Hemiphractid frogs
Combined mtDNA and nucDNA

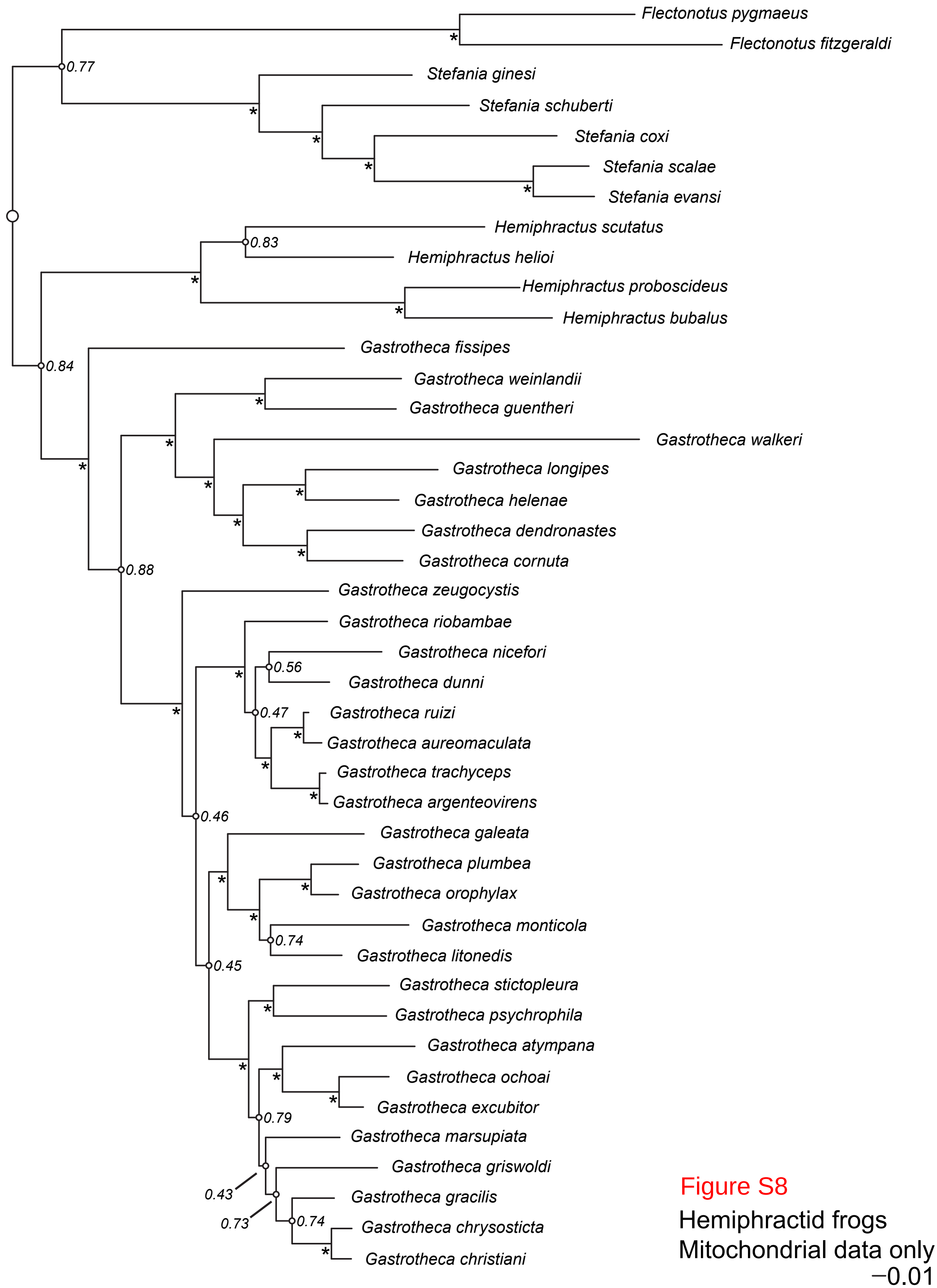

Figure S8

Hemiphractid frogs

Mitochondrial data only

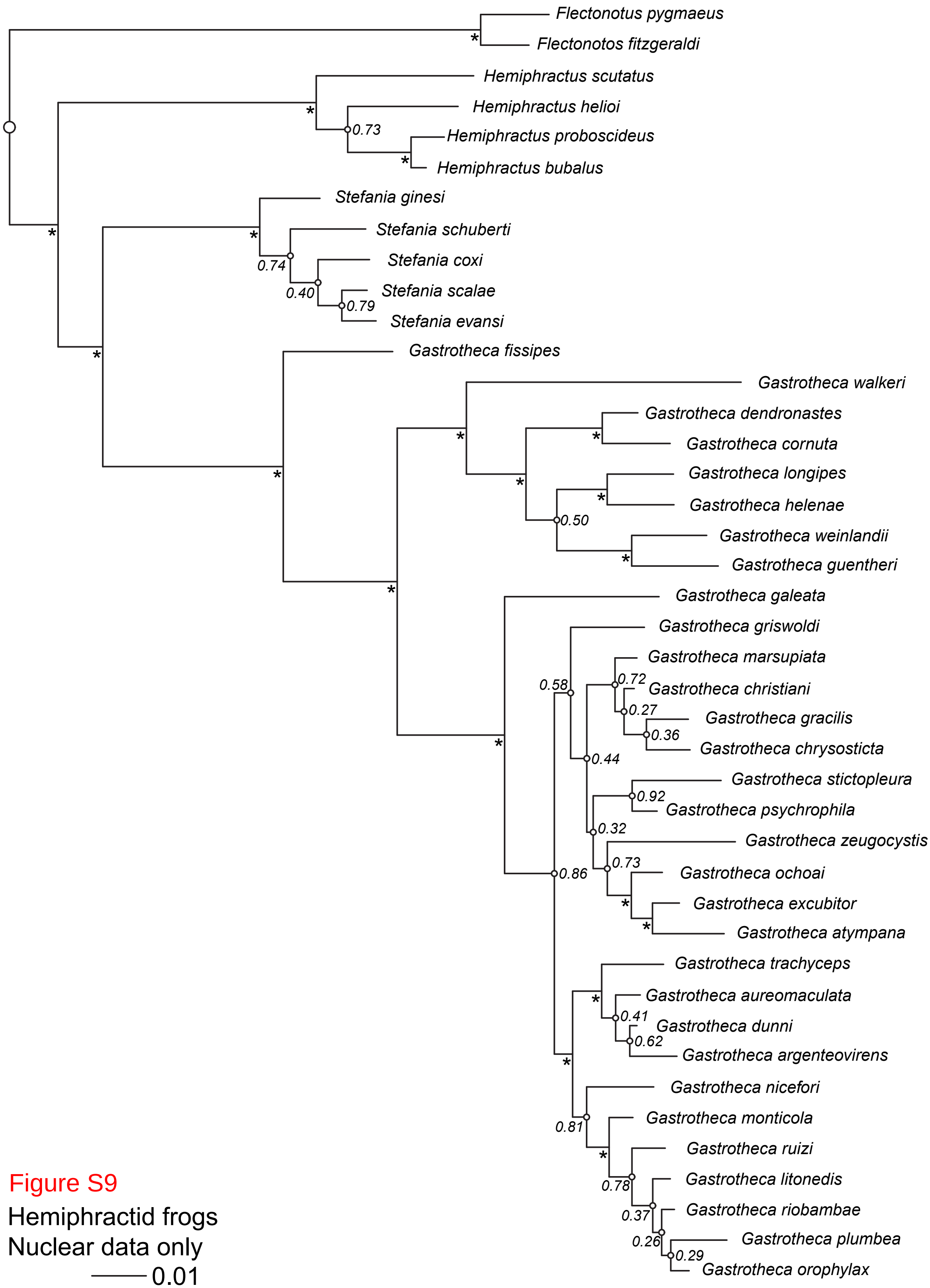

Figure S9

Hemiphractid frogs

Nuclear data only

0.01

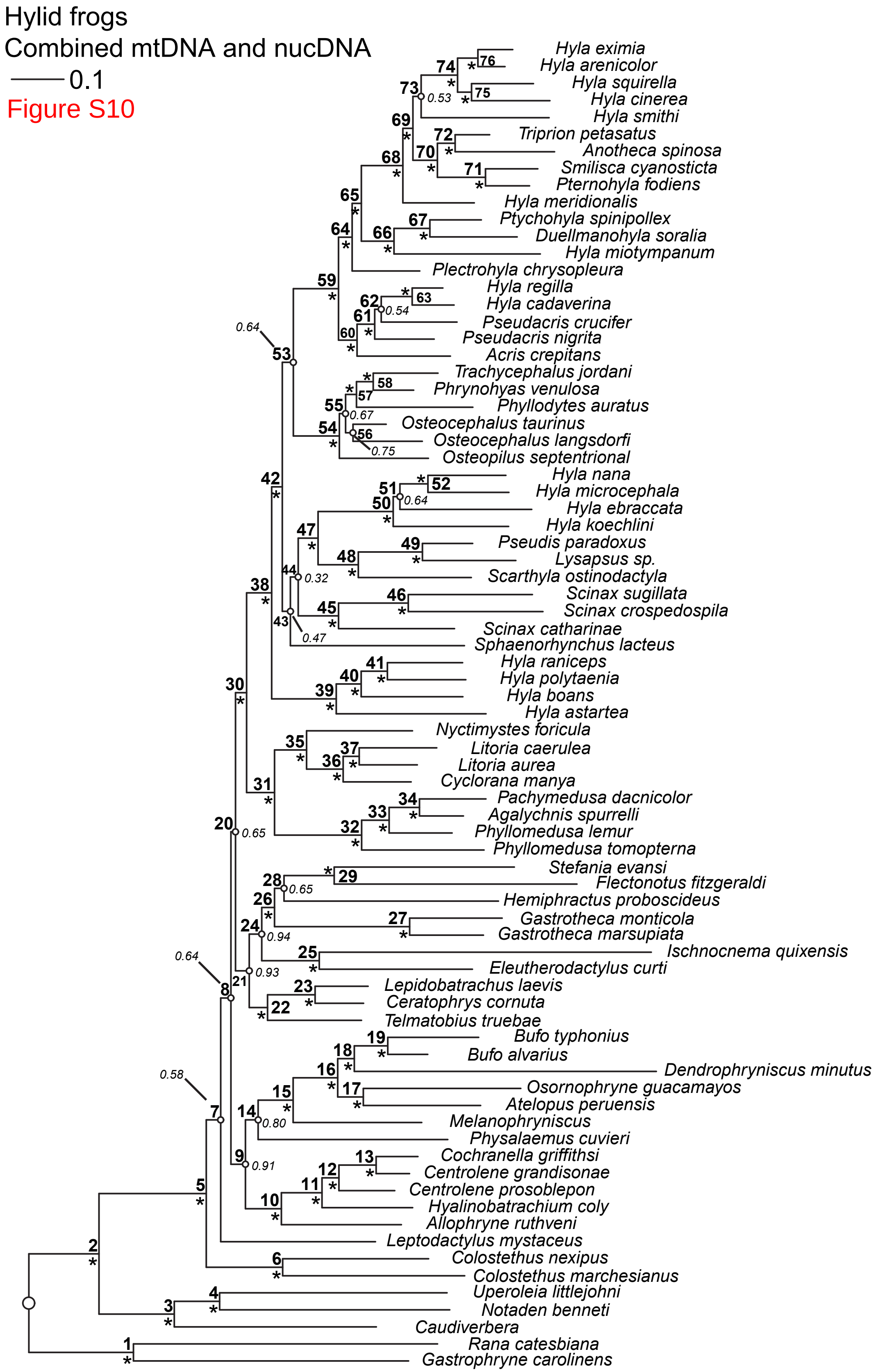
Hylid frogs
Combined mtDNA and nucDNA
0.1
Figure S10
Hyla eximia
Hyla arenicolor
Hyla squirella
Hyla cinerea
Hyla smithi
Triprion petasatus
Anotheca spinosa
Smilisca cyanosticta
Pternohyla fodiens
Hyla meridionalis
Ptychohyla spinipollex
Duellmanohyla soralia
Hyla miotympanum
Plectrohyla chrysopleura
Hyla regilla
Hyla cadaverina
Pseudacris crucifer
Pseudacris nigrita
Acris crepitans
Trachycephalus jordani
Phrynohyas venulosa
Phyllodytes auratus
Osteocephalus taurinus
Osteocephalus langsdorfi
Osteopilus septentrional
Hyla nana
Hyla microcephala
Hyla ebraccata
Hyla koechlini
Pseudis paradoxus
Lysapsus sp.
Scarthyla ostinodactyla
Scinax sugillata
Scinax crospedospila
Scinax catharinae
Sphaenorhynchus lacteus
Hyla raniceps
Hyla polytaenia
Hyla boans
Hyla astartea
Nyctimystes foricula
Litoria caerulea
Litoria aurea
Cyclorana manya
Pachymedusa dacnicolor
Agalychnis spurrelli
Phyllomedusa lemur
Phyllomedusa tomopterna
Stefania evansi
Flectonotus fitzgeraldi
Hemiphractus proboscideus
Gastrotheca monticola
Gastrotheca marsupiata
Ischnocnema quixensis
Eleutherodactylus curti
Lepidobatrachus laevis
Ceratophrys cornuta
Telmatobius truebae
Bufo typhonius
Bufo alvarius
Dendrophryniscus minutus
Osornophryne guacamayos
Atelopus peruensis
Melanophryniscus
Physalaemus cuvieri
Cochranella griffithsi
Centrolene grandisonae
Centrolene prosoblepon
Hyalinobatrachium coly
Allophryne ruthveni
Leptodactylus mystaceus
Colostethus nexipus
Colostethus marchesianus
Uperoleia littlejohni
Notaden benneti
Caudiverbera
Rana catesbiana
Gastrophryne carolinens

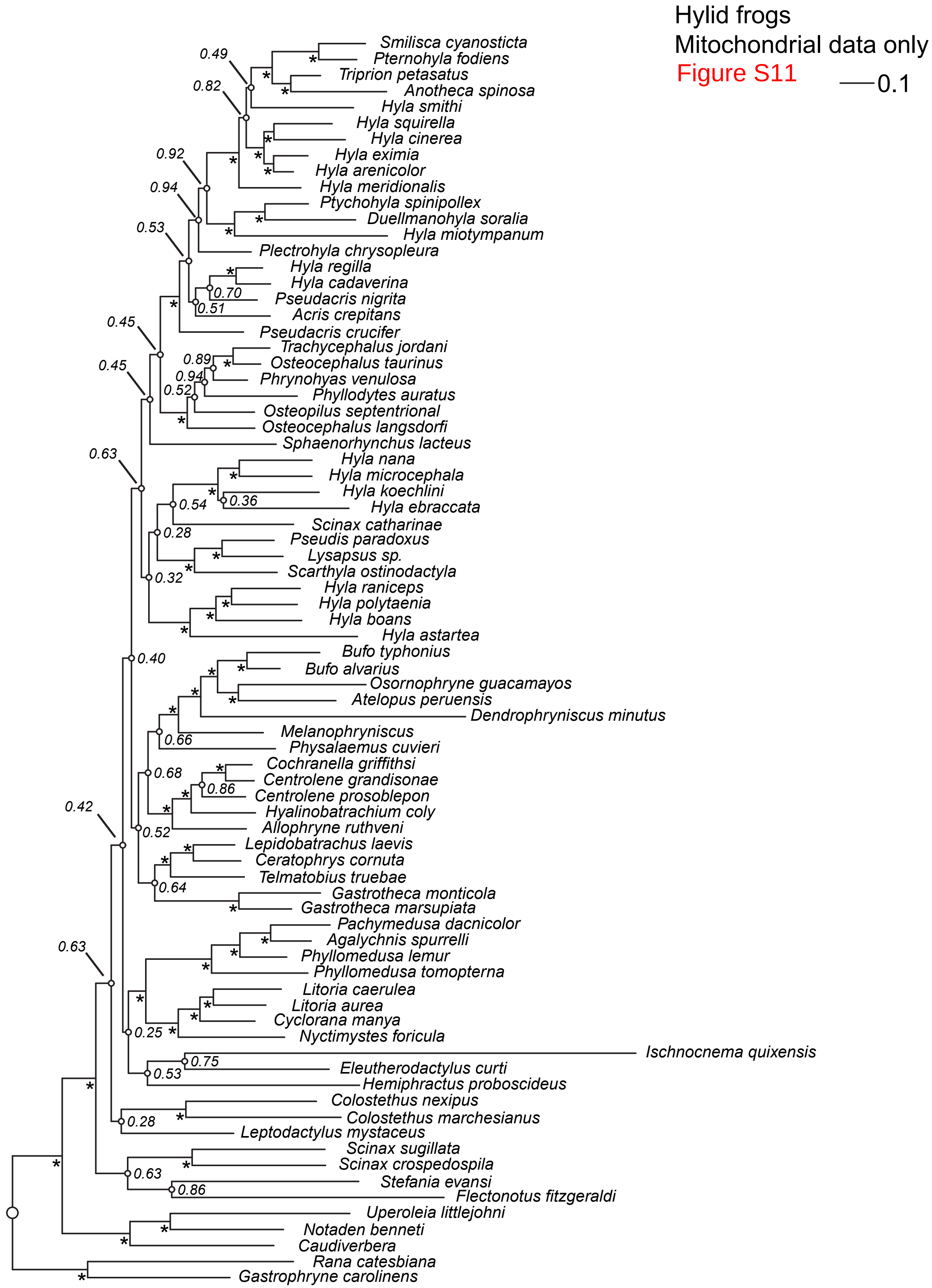

Hylid frogs
Mitochondrial data only
Figure S11
0.1
Smilisca cyanosticta
Pternohyla fodiens
Triprion petasatus
Anotheca spinosa
Hyla smithi
Hyla squirella
Hyla cinerea
Hyla eximia
Hyla arenicolor
Hyla meridionalis
Ptychohyla spinipollex
Duellmanohyla soralia
Hyla miotympanum
Plectrohyla chrysopleura
Hyla regilla
Hyla cadaverina
Pseudacris nigrita
Acris crepitans
Pseudacris crucifer
Trachycephalus jordani
Osteocephalus taurinus
Phrynohyas venulosa
Phyllodytes auratus
Osteopilus septentrional
Osteocephalus langsdorfi
Sphaenorhynchus lacteus
Hyla nana
Hyla microcephala
Hyla koechlini
Hyla ebraccata
Scinax catharinae
Pseudis paradoxus
Lysapsus sp.
Scarthyla ostinodactyla
Hyla raniceps
Hyla polytaenia
Hyla boans
Hyla astartea
Bufo typhonius
Bufo alvarius
Osornophryne guacamayos
Atelopus peruensis
Dendrophryniscus minutus
Melanophryniscus
Physalaemus cuvieri
Cochranella griffithsi
Centrolene grandisonae
Centrolene prosoblepon
Hyalinobatrachium coly
Allophryne ruthveni
Lepidobatrachus laevis
Ceratophrys cornuta
Telmatobius truebae
Gastrotheca monticola
Gastrotheca marsupiata
Pachymedusa dacnicolor
Agalychnis spurrelli
Phyllomedusa lemur
Phyllomedusa tomopterna
Litoria caerulea
Litoria aurea
Cyclorana manya
Nyctimystes foricula
Ischnocnema quixensis
Eleutherodactylus curti
Hemiphractus proboscideus
Colostethus nexipus
Colostethus marchesianus
Leptodactylus mystaceus
Scinax sugillata
Scinax crospedospila
Stefania evansi
Flectonotus fitzgeraldi
Uperoleia littlejohni
Notaden benneti
Caudiverbera
Rana catesbiana
Gastrophryne carolinens
0.49
0.82
0.92
0.94
0.53
0.70
0.51
0.45
0.89
0.94
0.52
0.45
0.63
0.54
0.36
0.28
0.32
0.40
0.66
0.68
0.86
0.52
0.42
0.64
0.63
0.25
0.75
0.53
0.28
0.63
0.86

Hylid frogs
Nuclear data only
Figure S12 −0.01

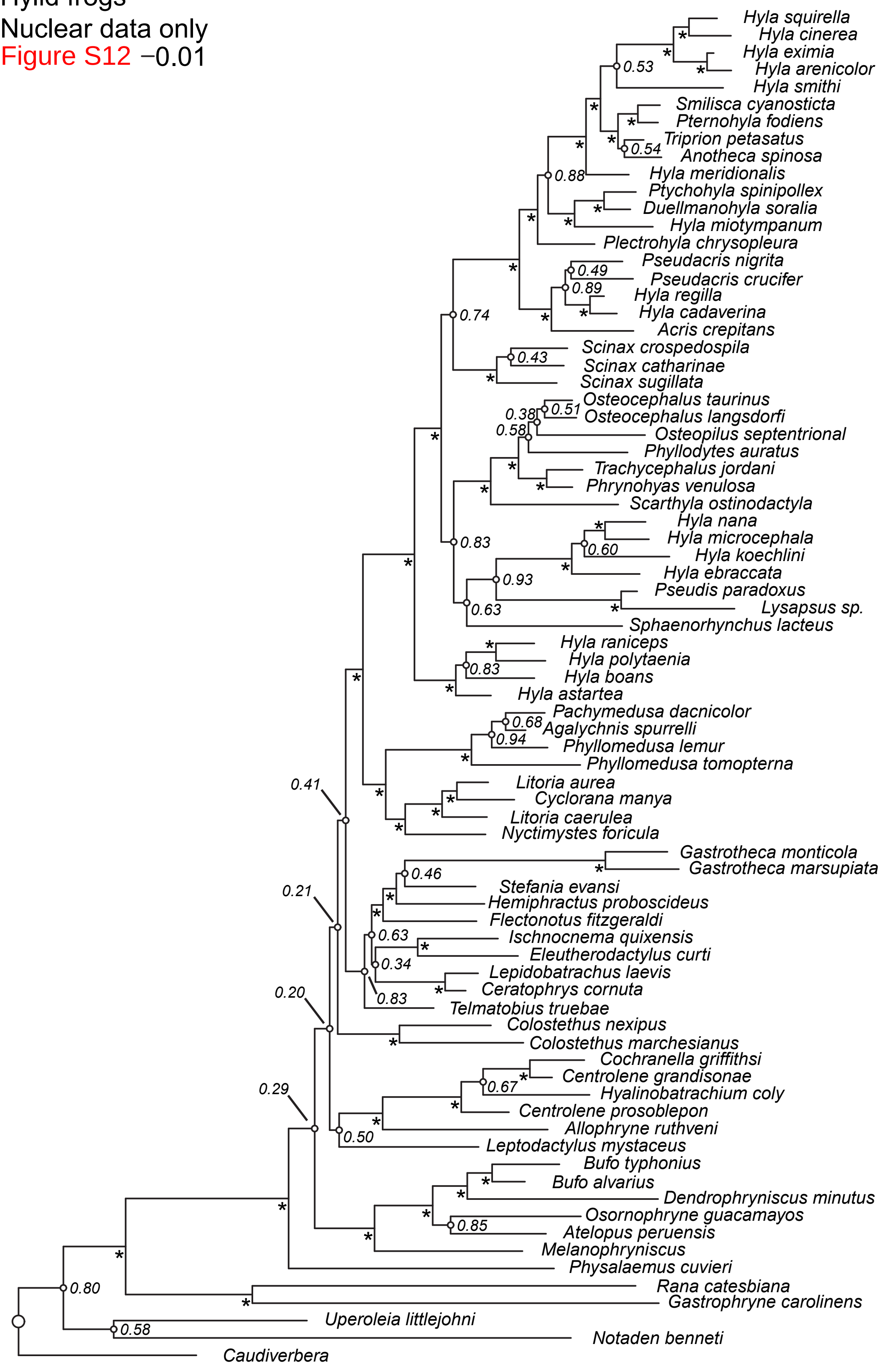

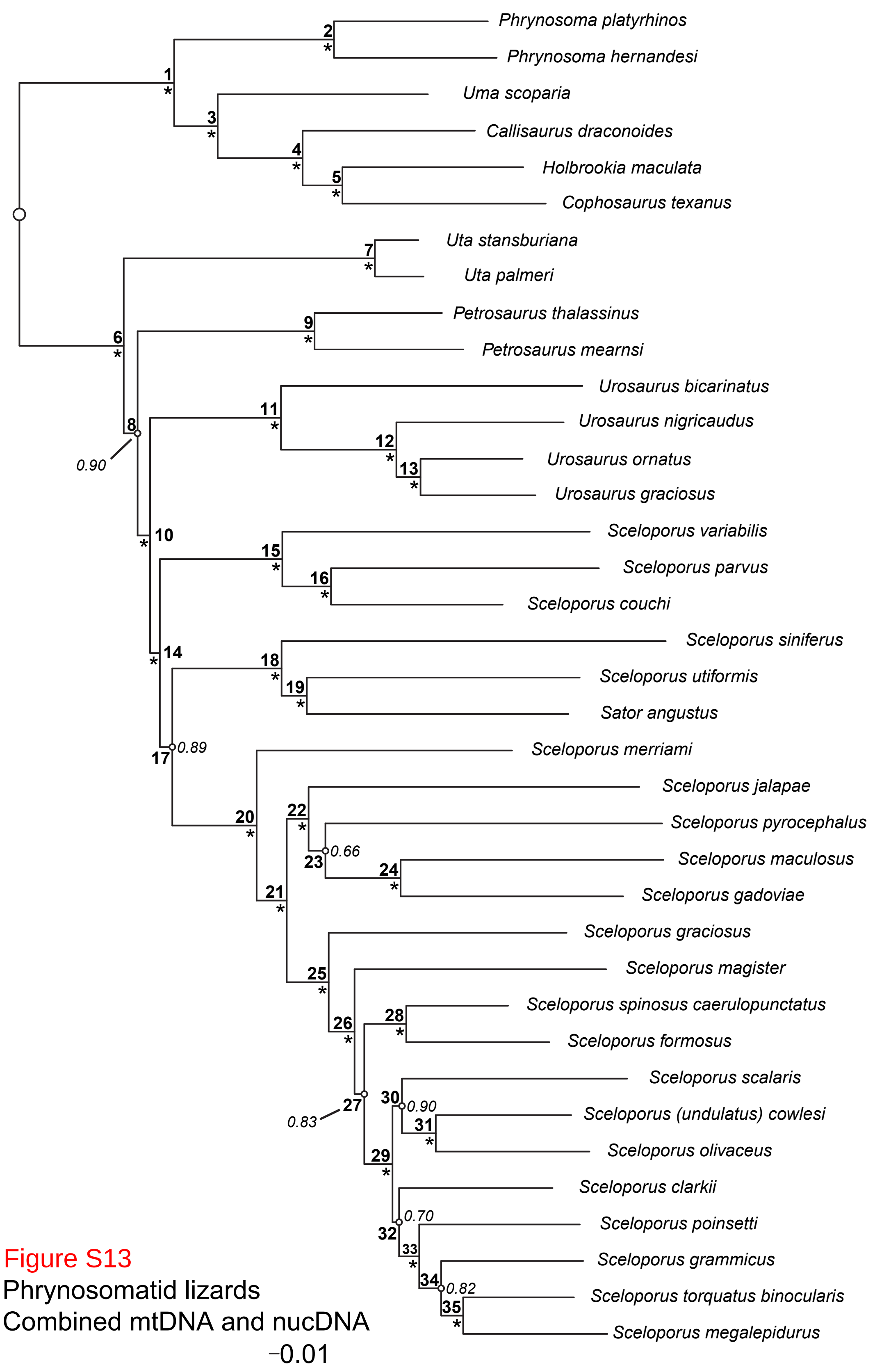

Figure S13
Phrynosomatid lizards
Combined mtDNA and nucDNA

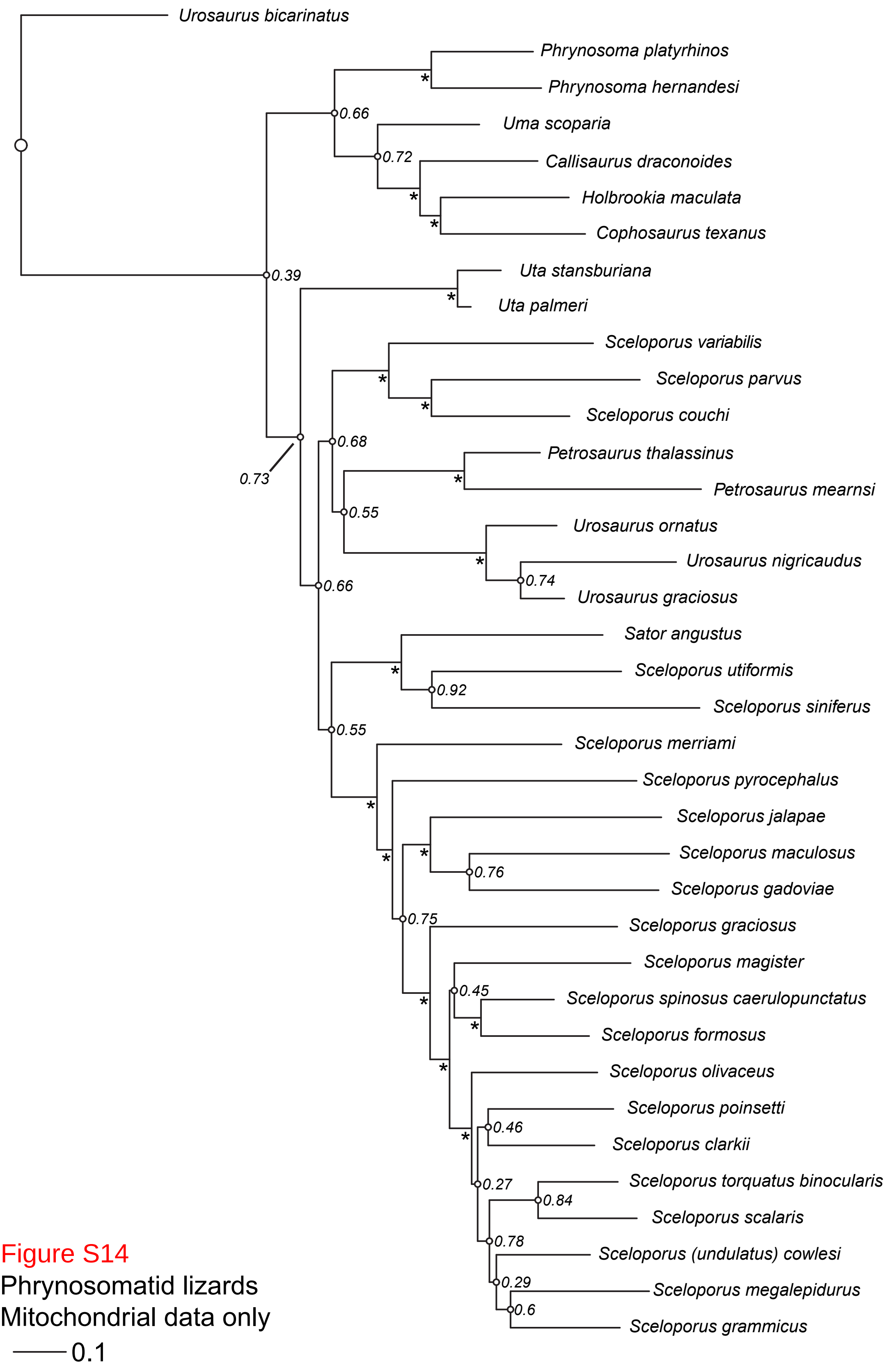

Figure S14
Phrynosomatid lizards
Mitochondrial data only

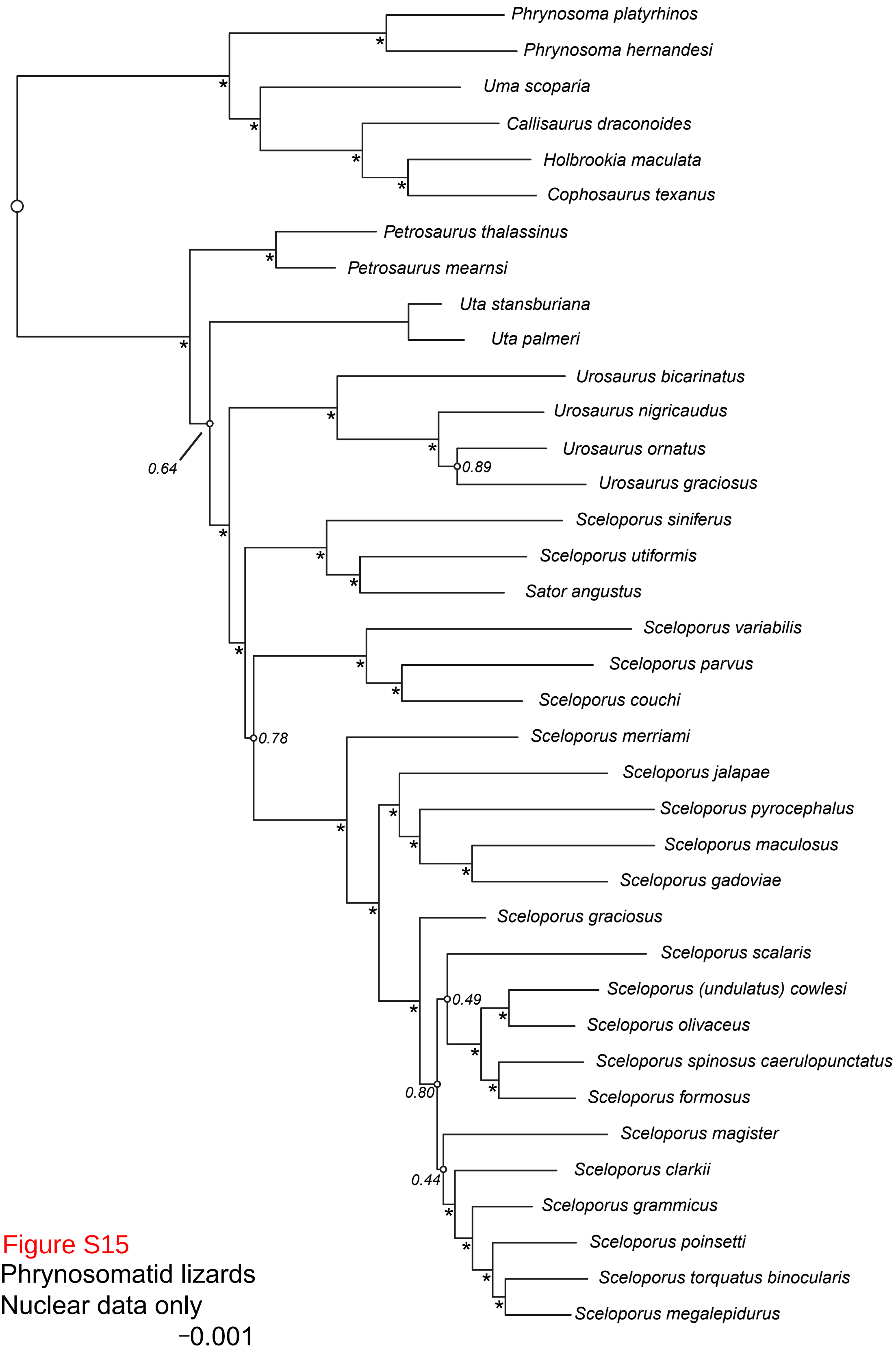

Figure S15
Phrynosomatid lizards
Nuclear data only

Alcid Birds
Combined mtDNA and nucDNA
Figure S16

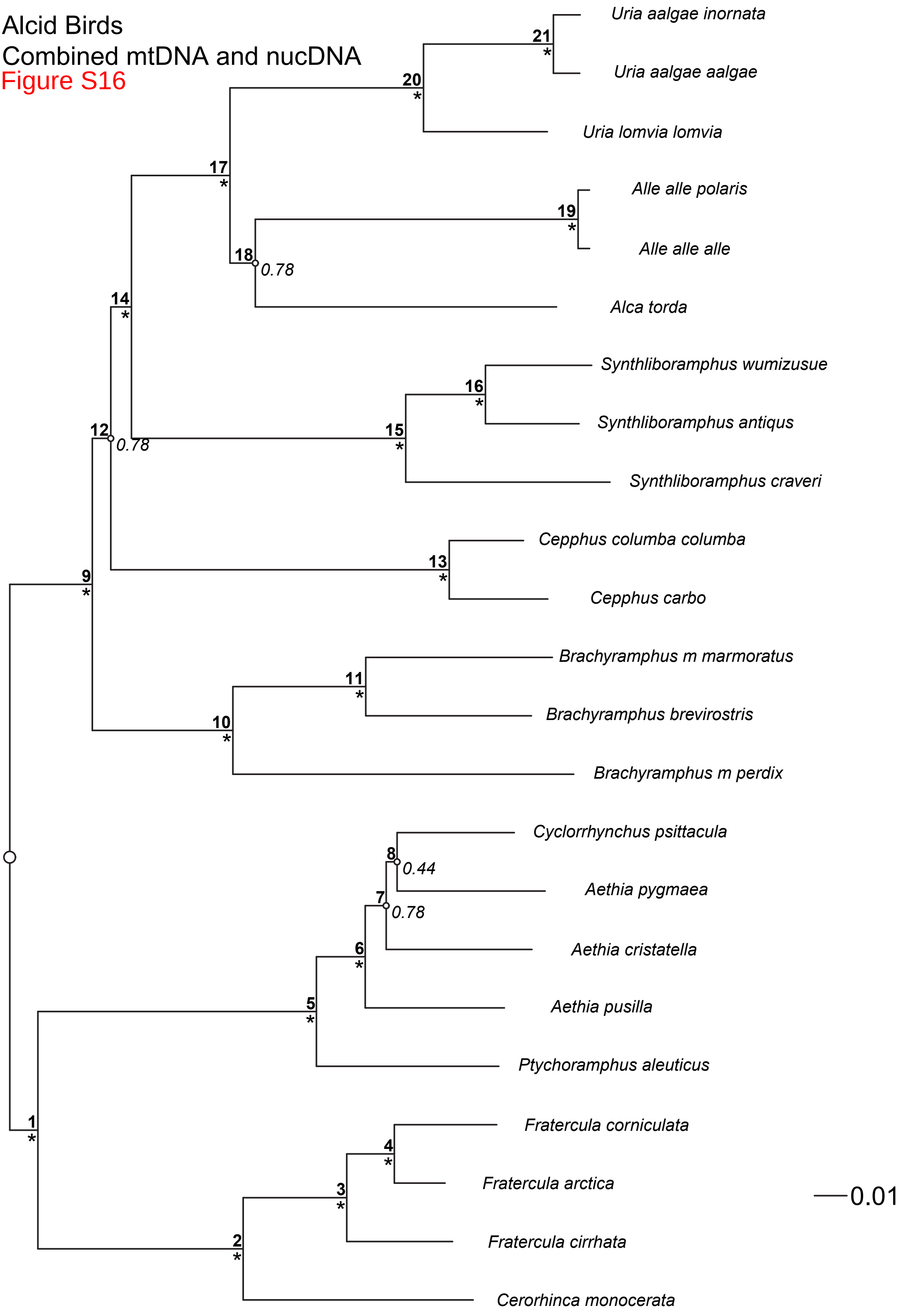

Alcid birds
Mitochondrial data only
Figure S17

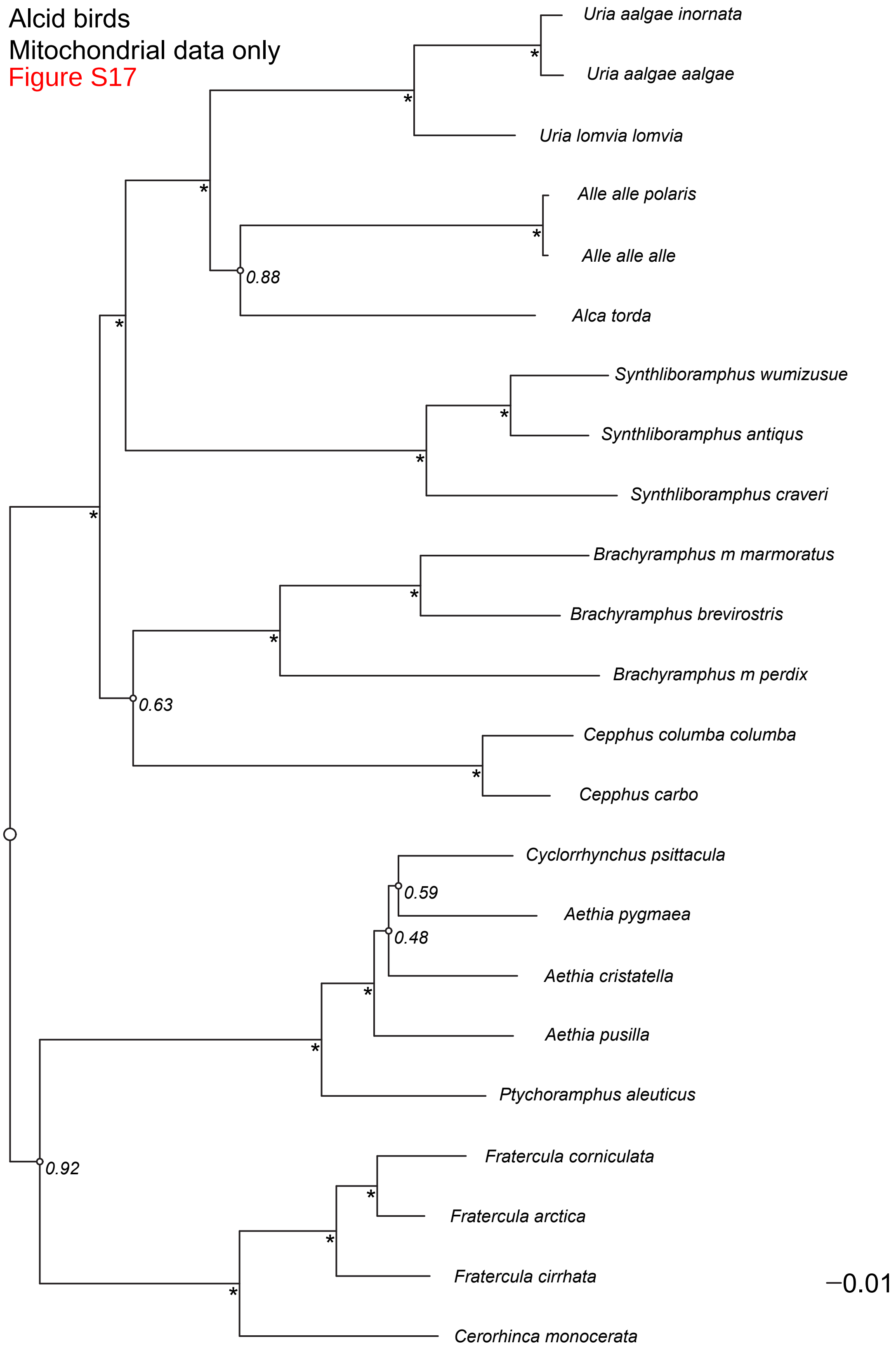

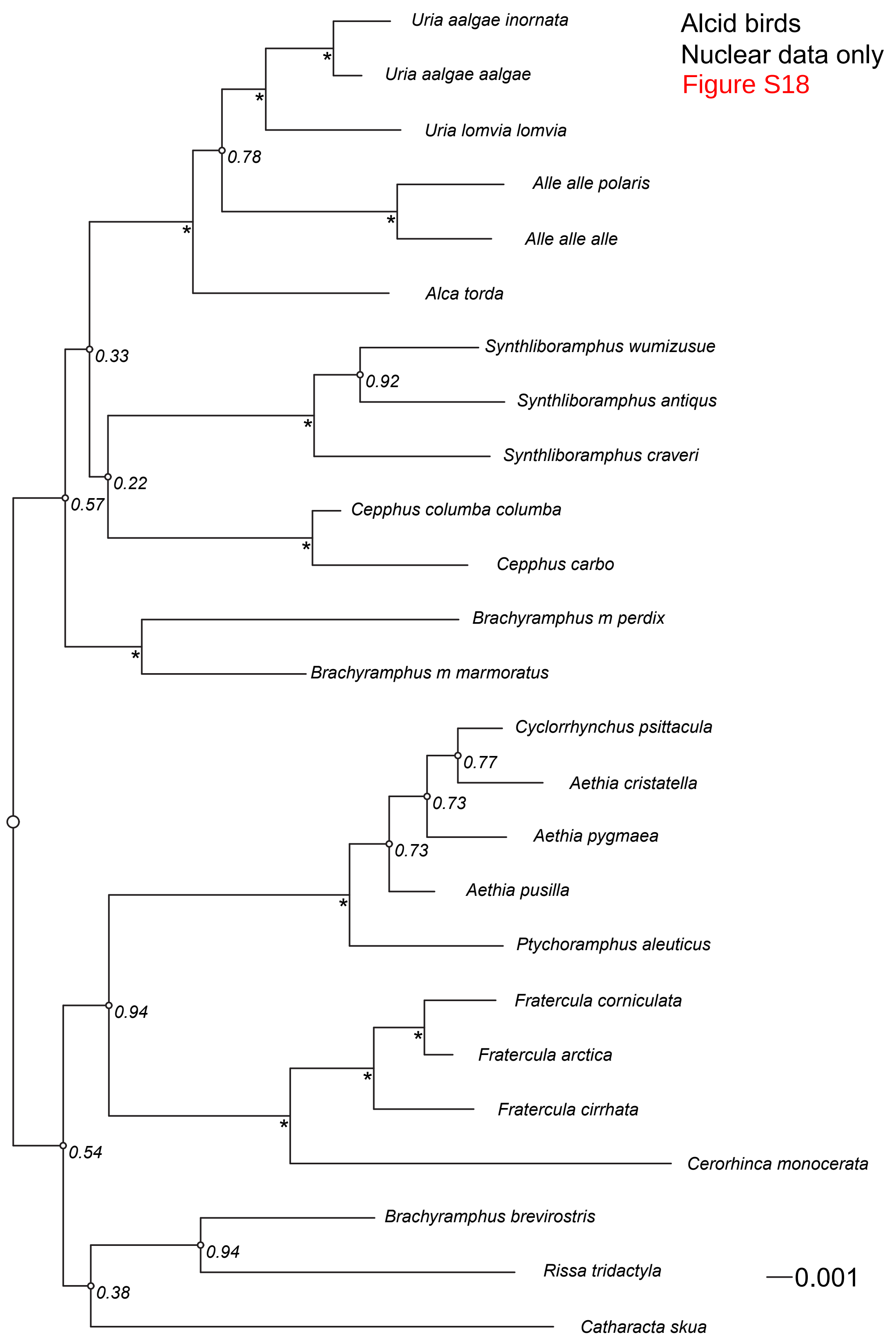
Alcid birds
Nuclear data only
Figure S18
Uria aalgae inornata
Uria aalgae aalgae
Uria lomvia lomvia
Alle alle polaris
Alle alle alle
Alca torda
Synthliboramphus wumizusue
Synthliboramphus antiqus
Synthliboramphus craveri
Cepphus columba columba
Cepphus carbo
Brachyramphus m perdix
Brachyramphus m marmoratus
Cyclorrhynchus psittacula
Aethia cristatella
Aethia pygmaea
Aethia pusilla
Ptychoramphus aleuticus
Fratercula corniculata
Fratercula arctica
Fratercula cirrhata
Cerorhinca monocerata
Brachyramphus brevirostris
Rissa tridactyla
Catharacta skua
0.78
0.33
0.92
0.22
0.57
0.77
0.73
0.73
0.94
0.54
0.94
0.38
0.001

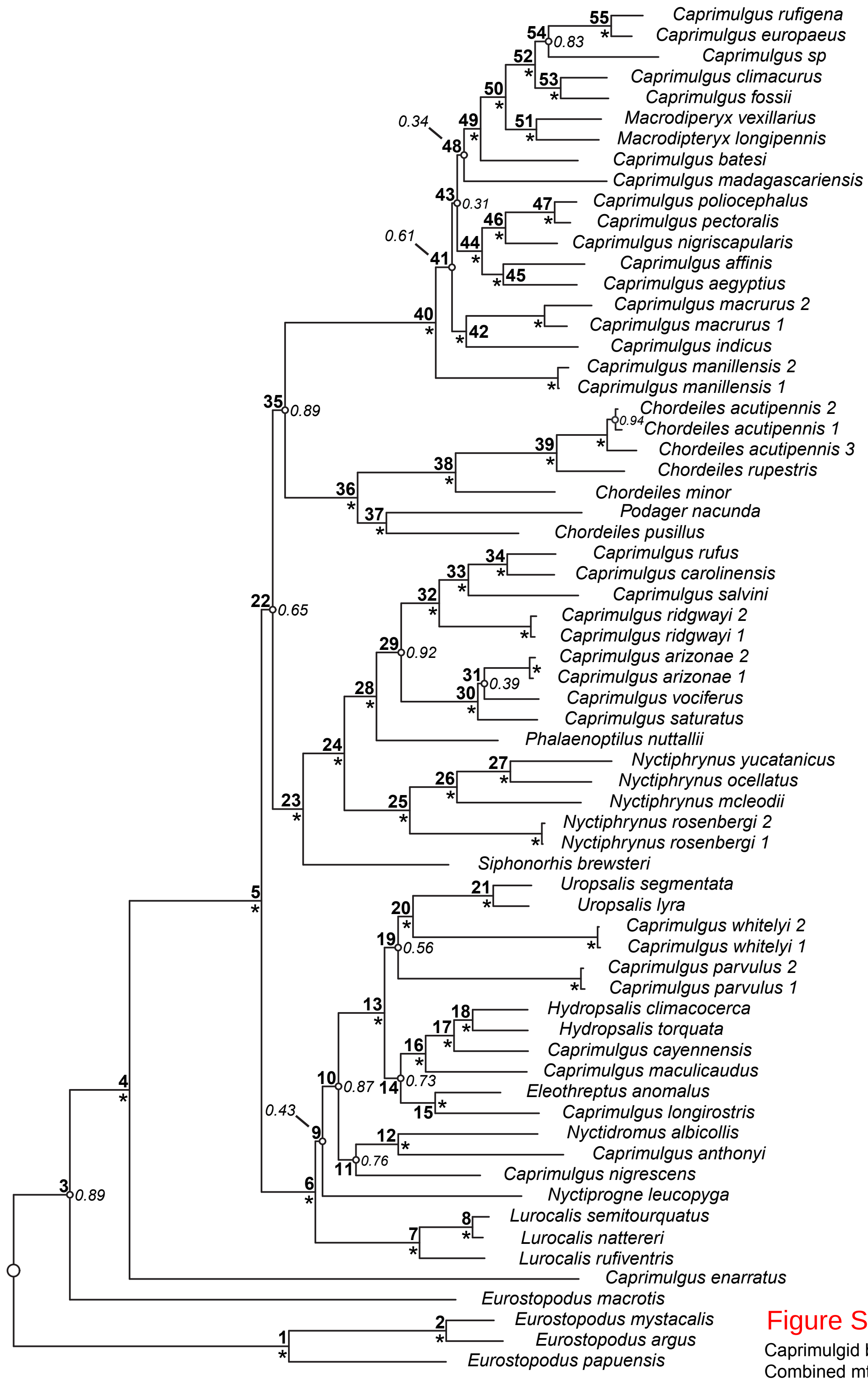

Figure S19

Caprimulgid birds

Combined mtDNA and nucDNA

Caprimulgid birds
Mitochondrial data only
Figure S20

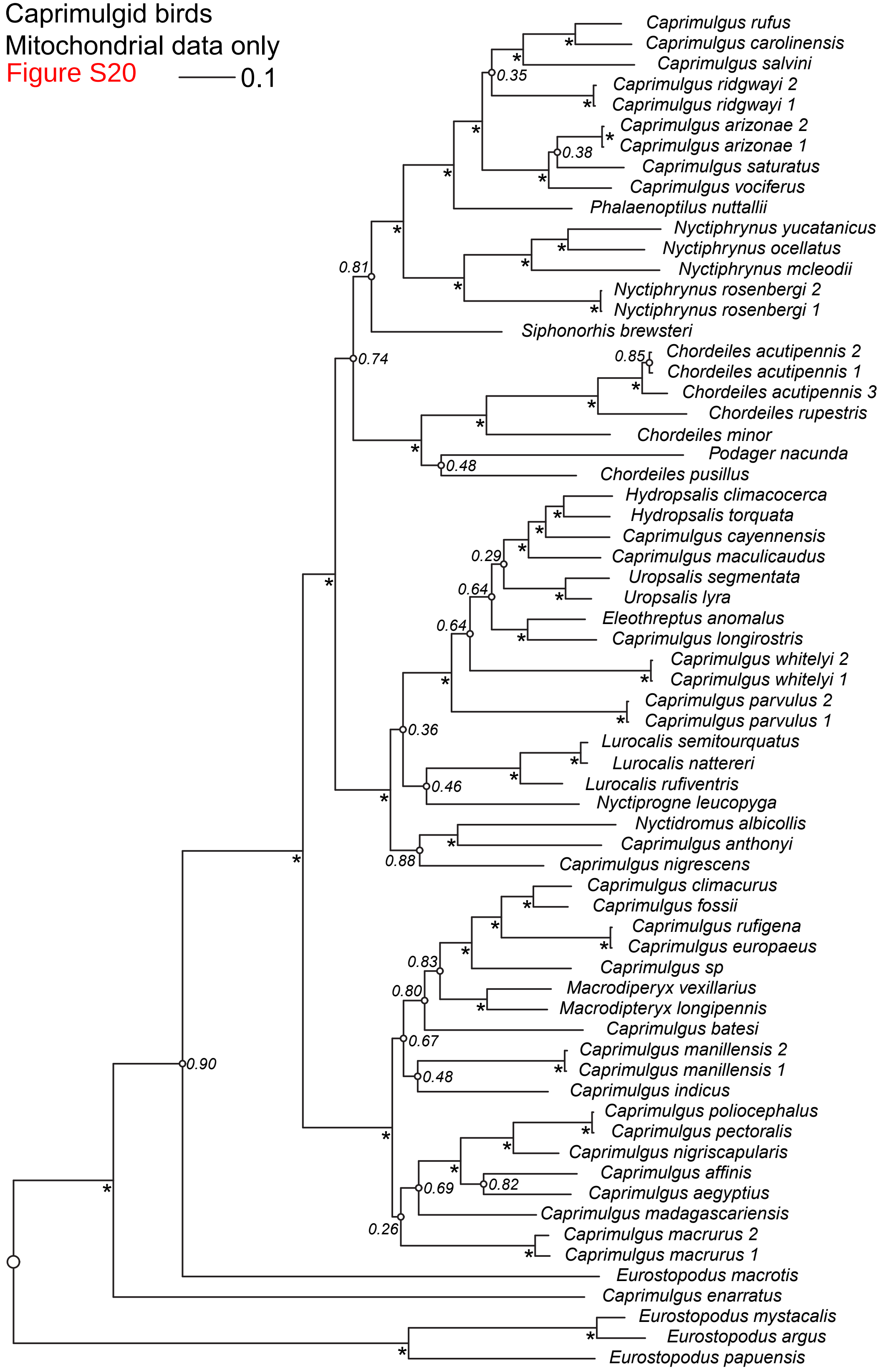

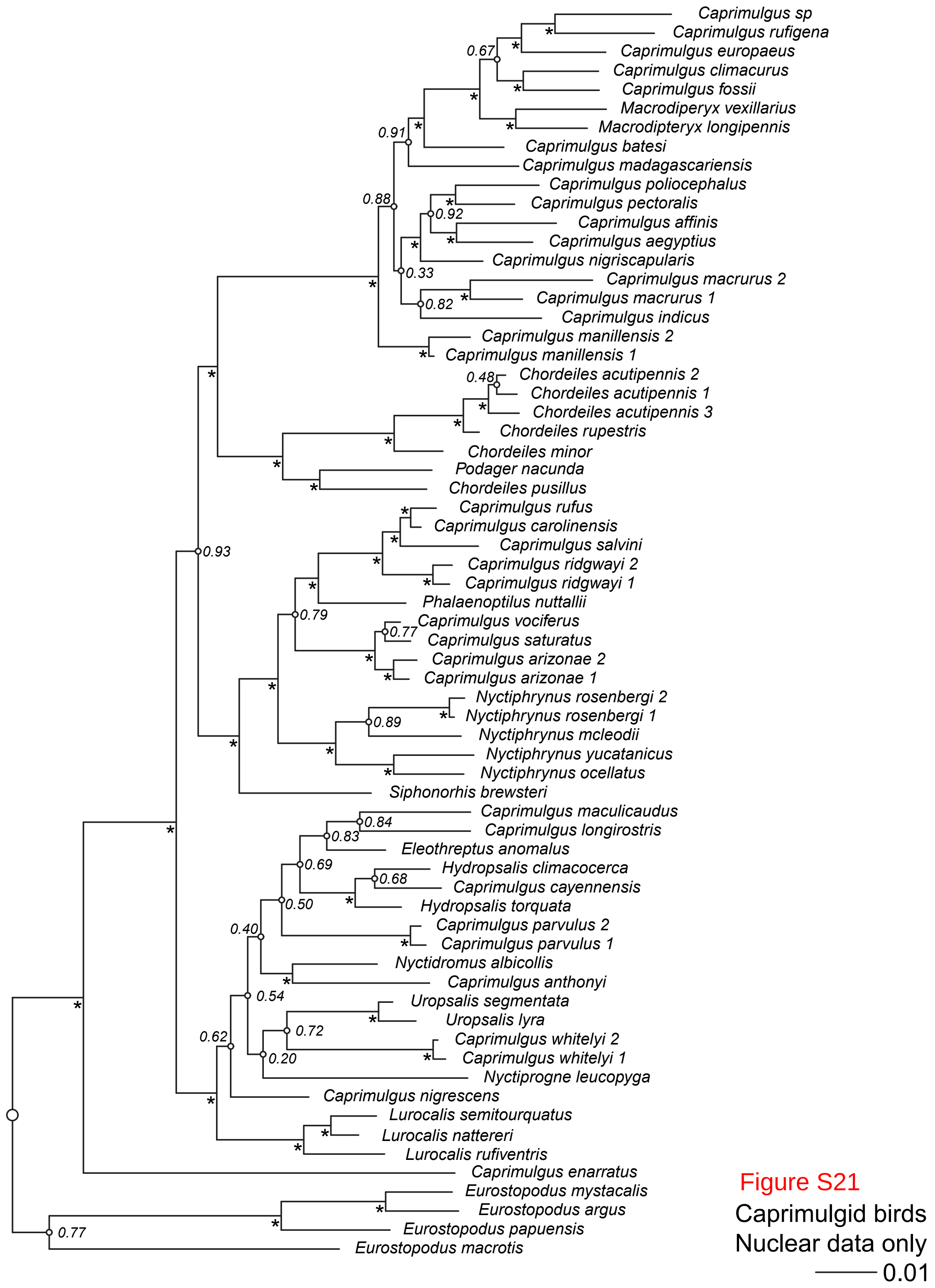

Figure S21
Caprimulgid birds
Nuclear data only

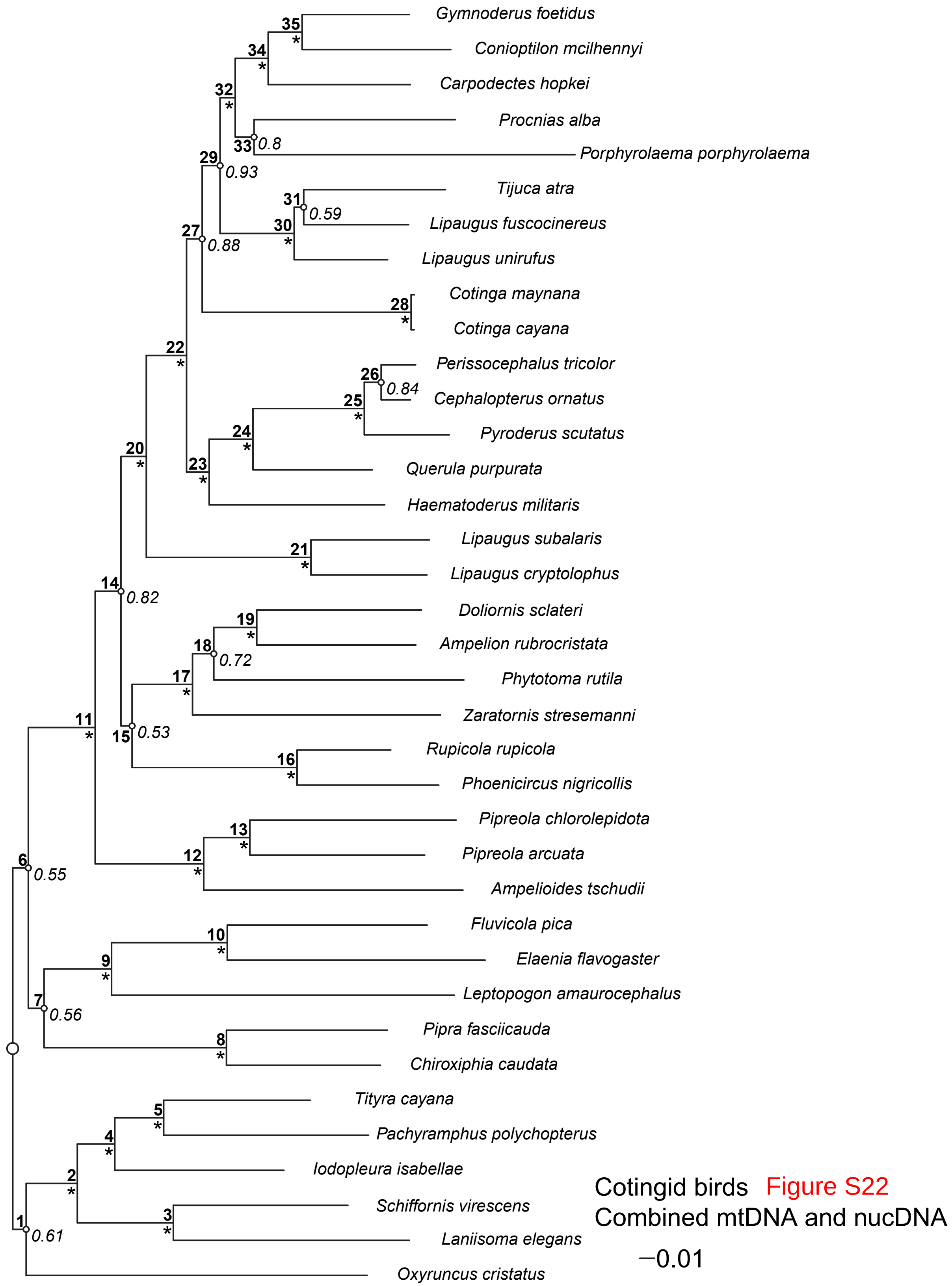
Gymnoderus foetidus
Conioptilon mcilhennyi
Carpodectes hopkei
Procnias alba
Porphyrolaema porphyrolaema
Tijuca atra
Lipaugus fuscocinereus
Lipaugus unirufus
Cotinga maynana
Cotinga cayana
Perissocephalus tricolor
Cephalopterus ornatus
Pyroderus scutatus
Querula purpurata
Haematoderus militaris
Lipaugus subalaris
Lipaugus cryptolophus
Doliornis sclateri
Ampelion rubrocristata
Phytotoma rutila
Zaratornis stresemanni
Rupicola rupicola
Phoenicircus nigricollis
Pipreola chlorolepidota
Pipreola arcuata
Ampelioides tschudii
Fluvicola pica
Elaenia flavogaster
Leptopogon amaurocephalus
Pipra fasciicauda
Chiroxiphia caudata
Tityra cayana
Pachyramphus polychopterus
Iodopleura isabellae
Schiffornis virescens
Laniisoma elegans
Oxyruncus cristatus
Cotingid birds
Figure S22
Combined mtDNA and nucDNA
0.01

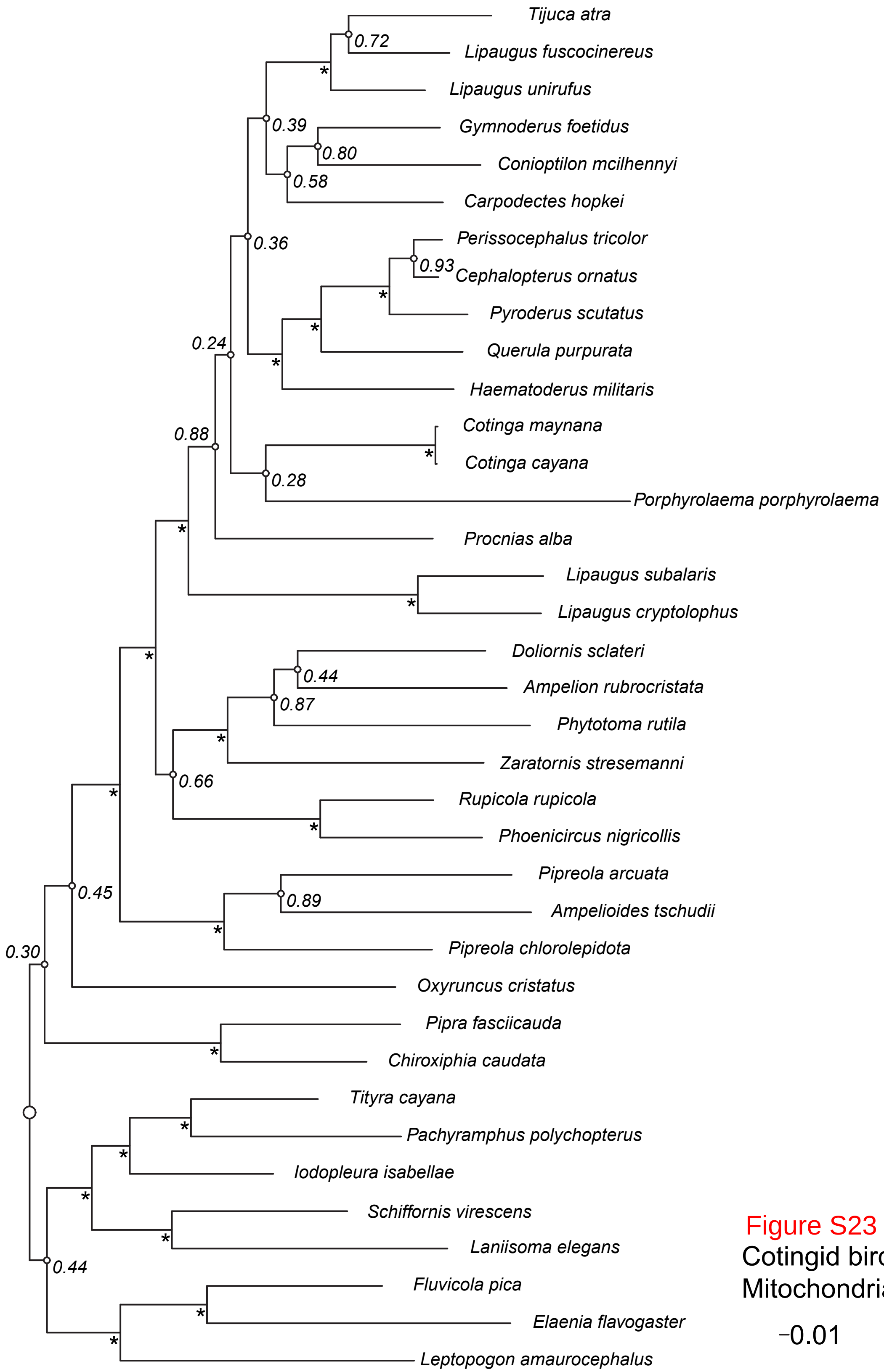

Figure S23

Cotingid birds

Mitochondrial data only

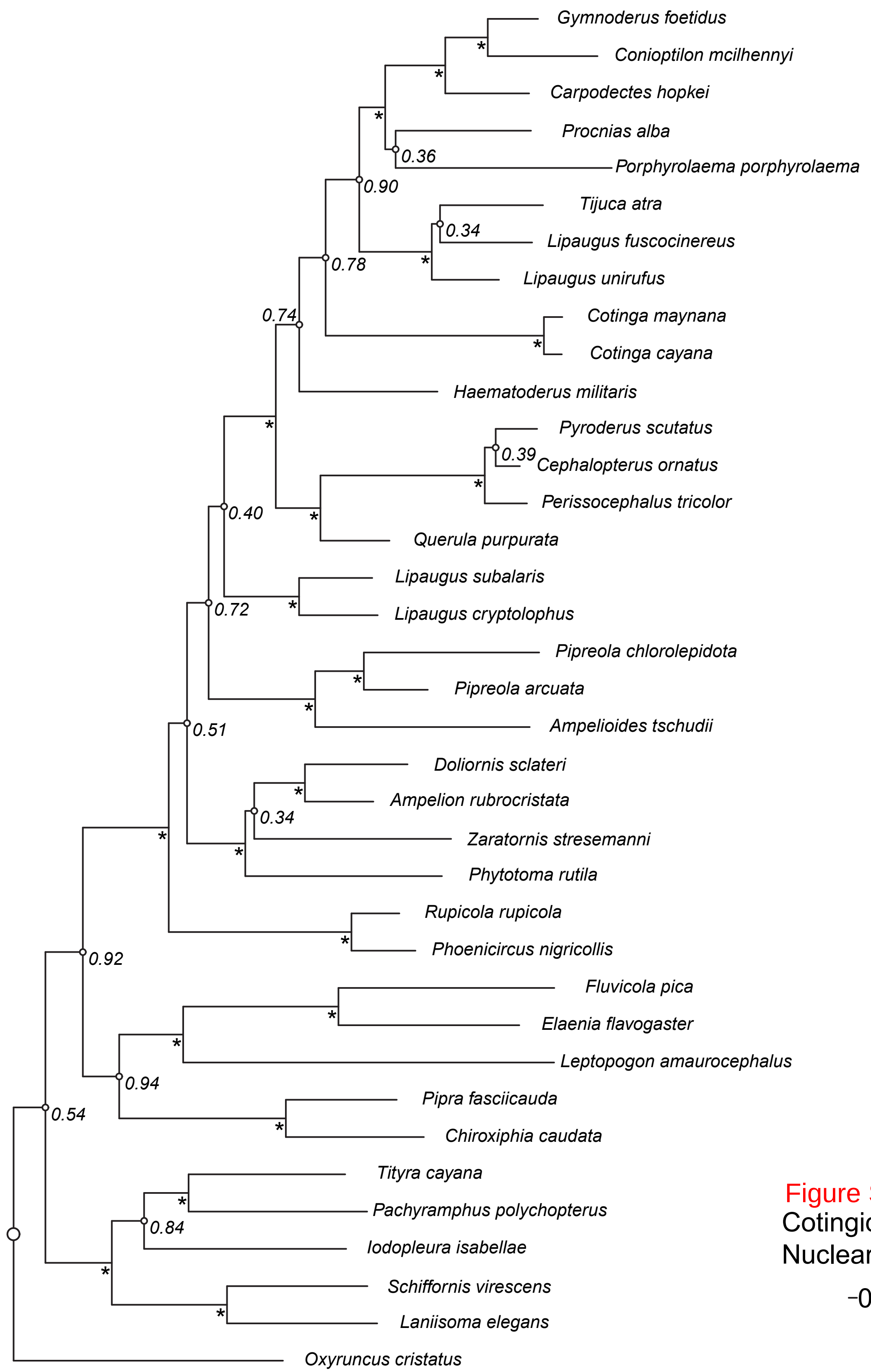

Figure S24
Cotingid birds
Nuclear data only

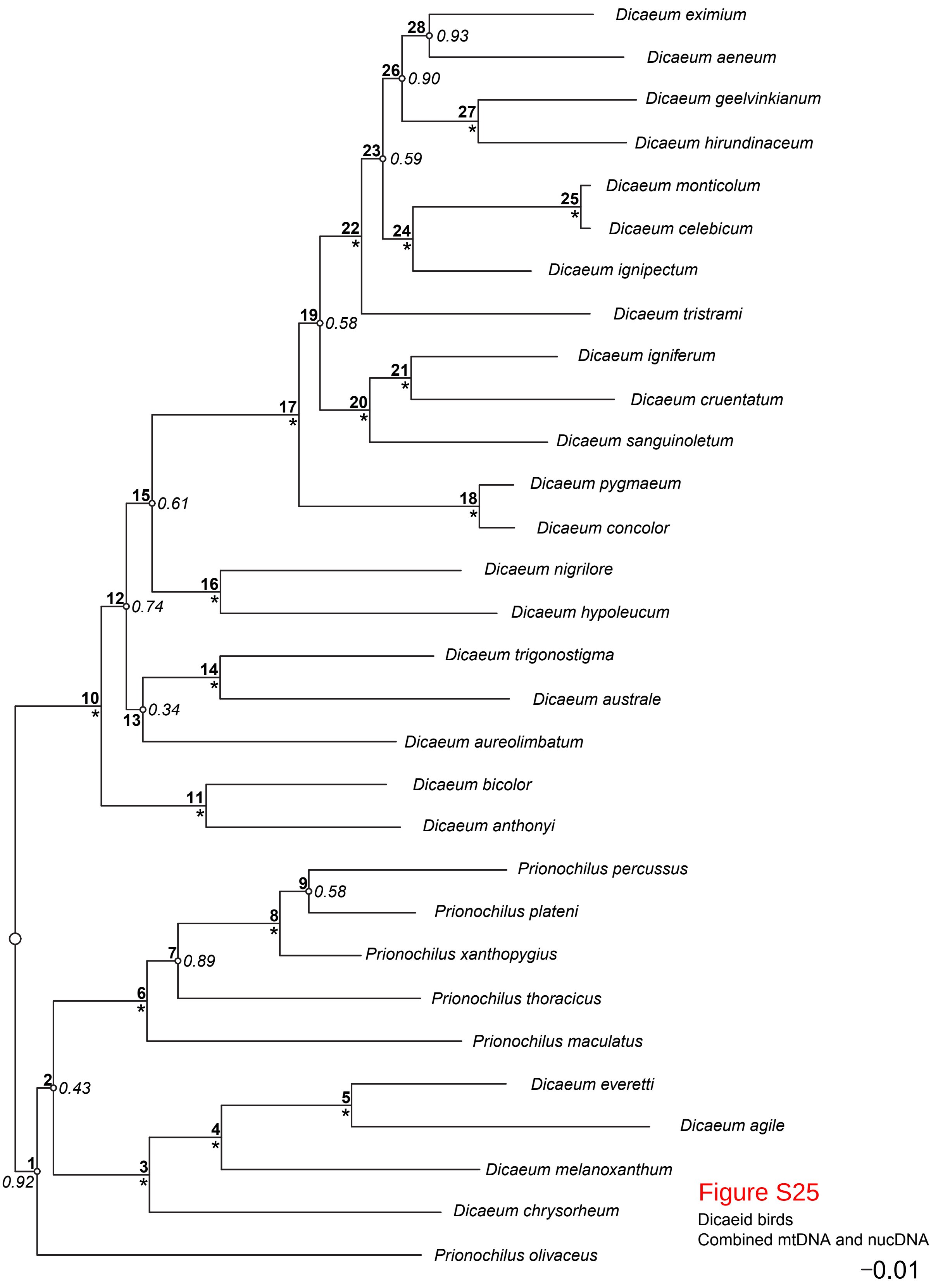

Figure S25
Dicaeid birds
Combined mtDNA and nucDNA

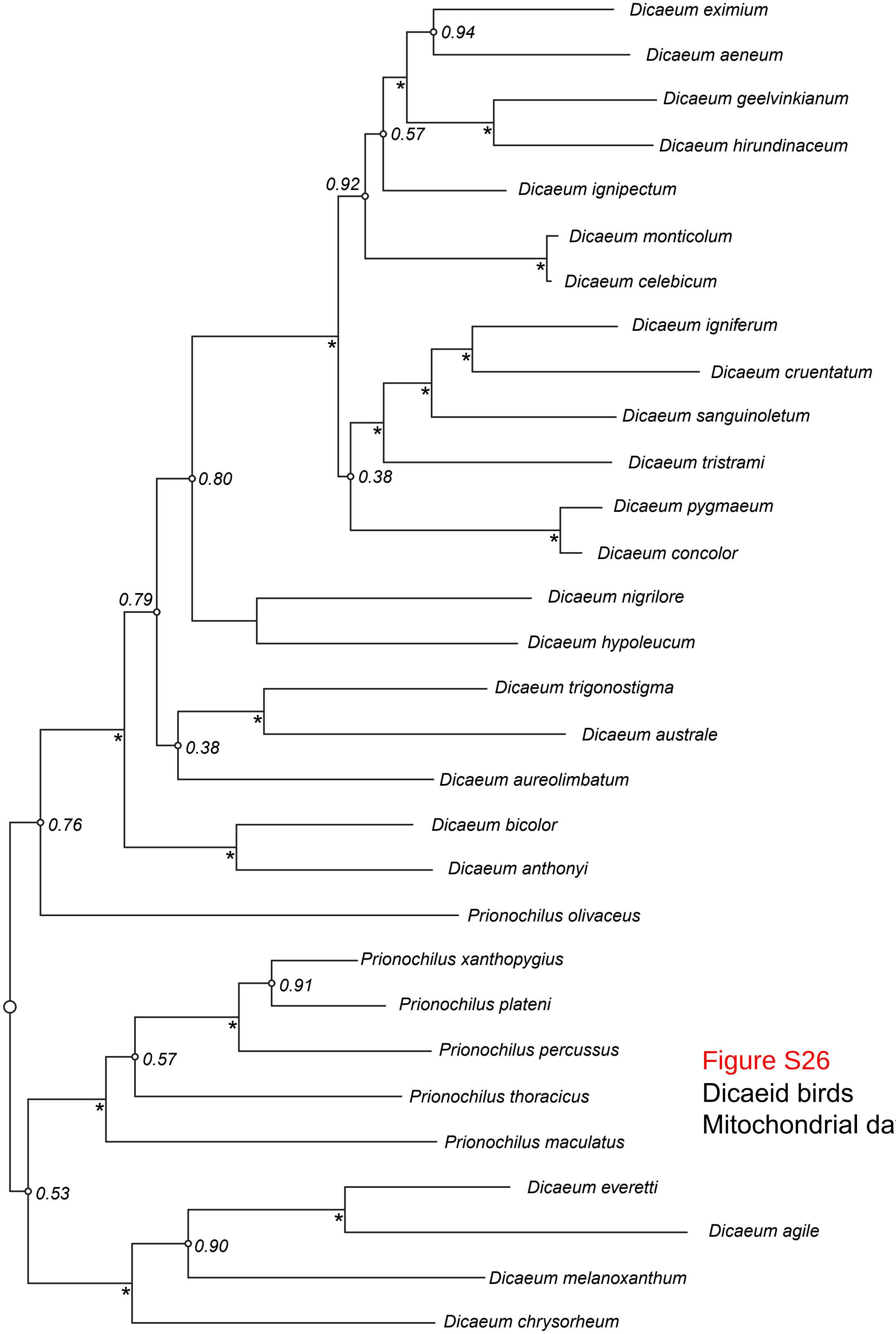

Figure S26
Dicaeid birds
Mitochondrial data only

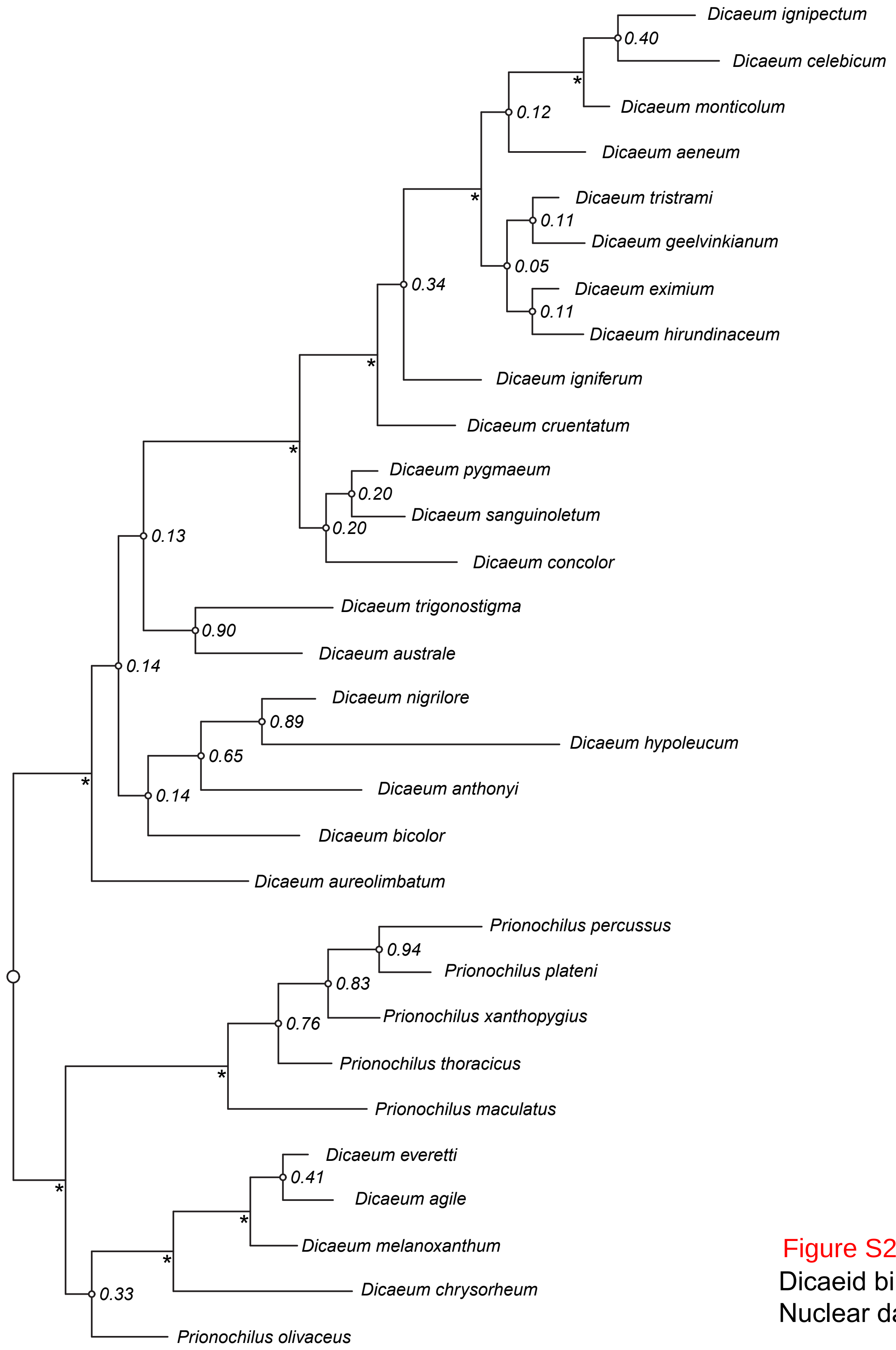

Figure S27

Dicaeid birds

Nuclear data only

−0.01

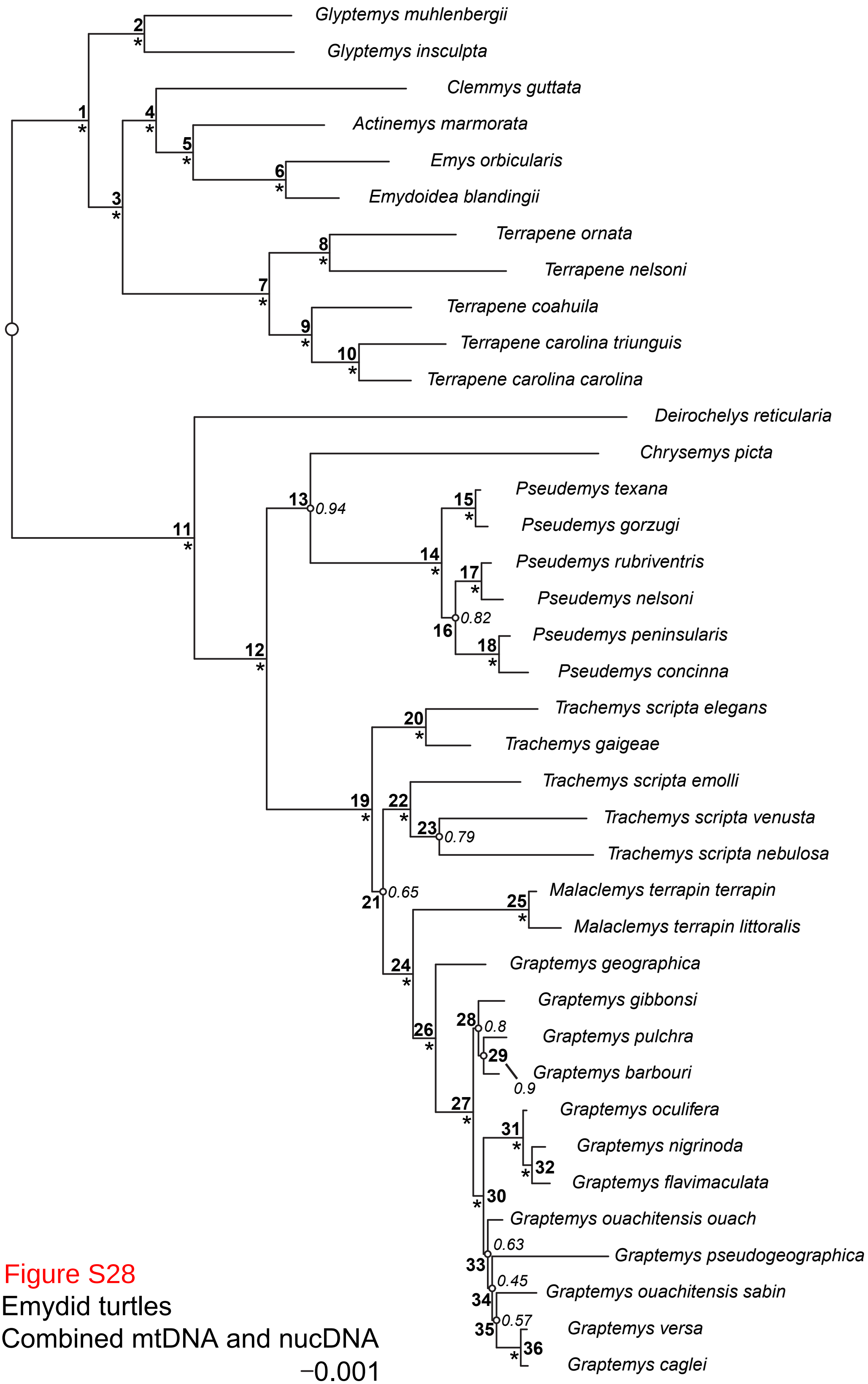

Figure S28
Emydid turtles
Combined mtDNA and nucDNA

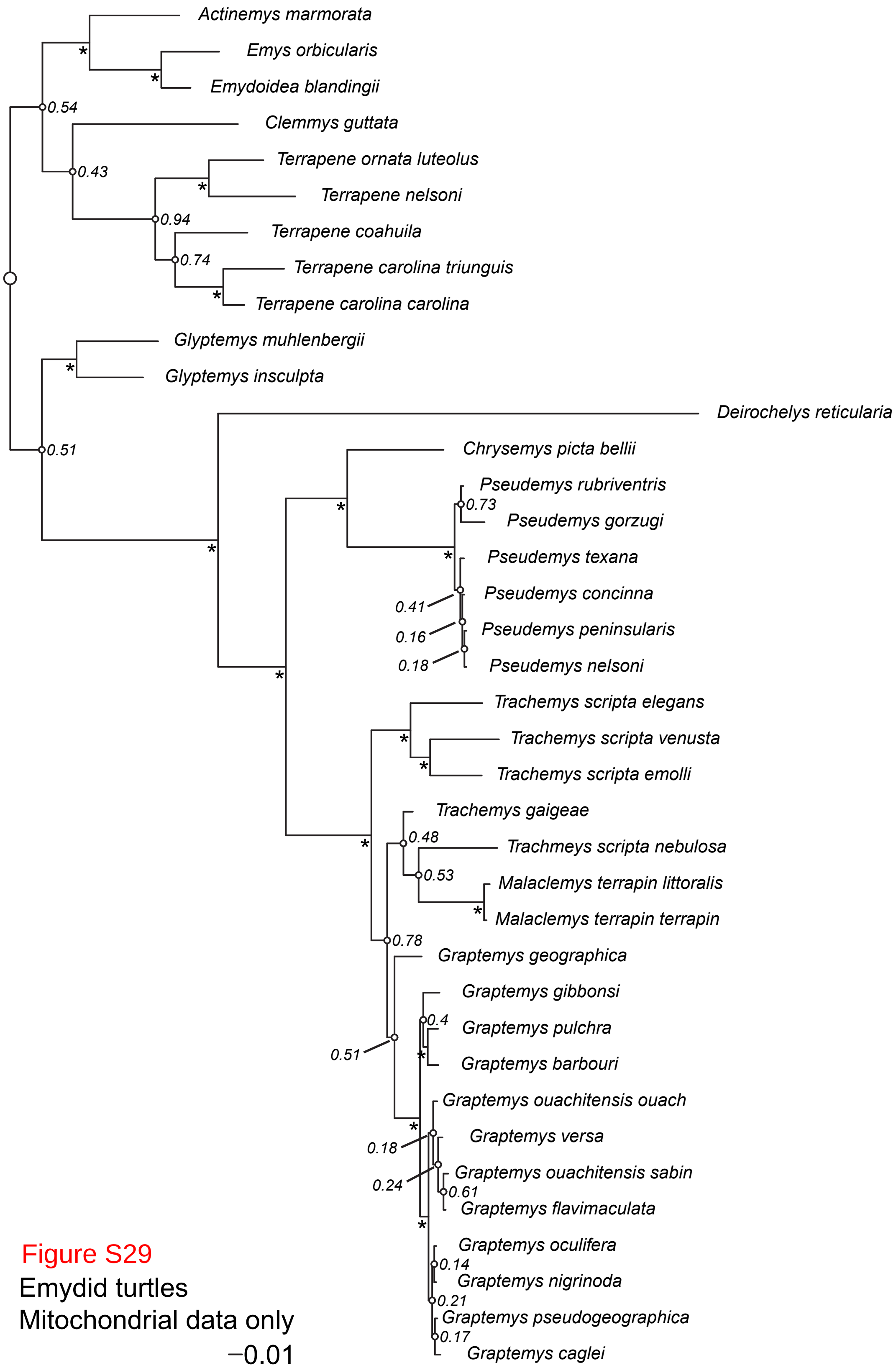

Figure S29
Emydid turtles
Mitochondrial data only

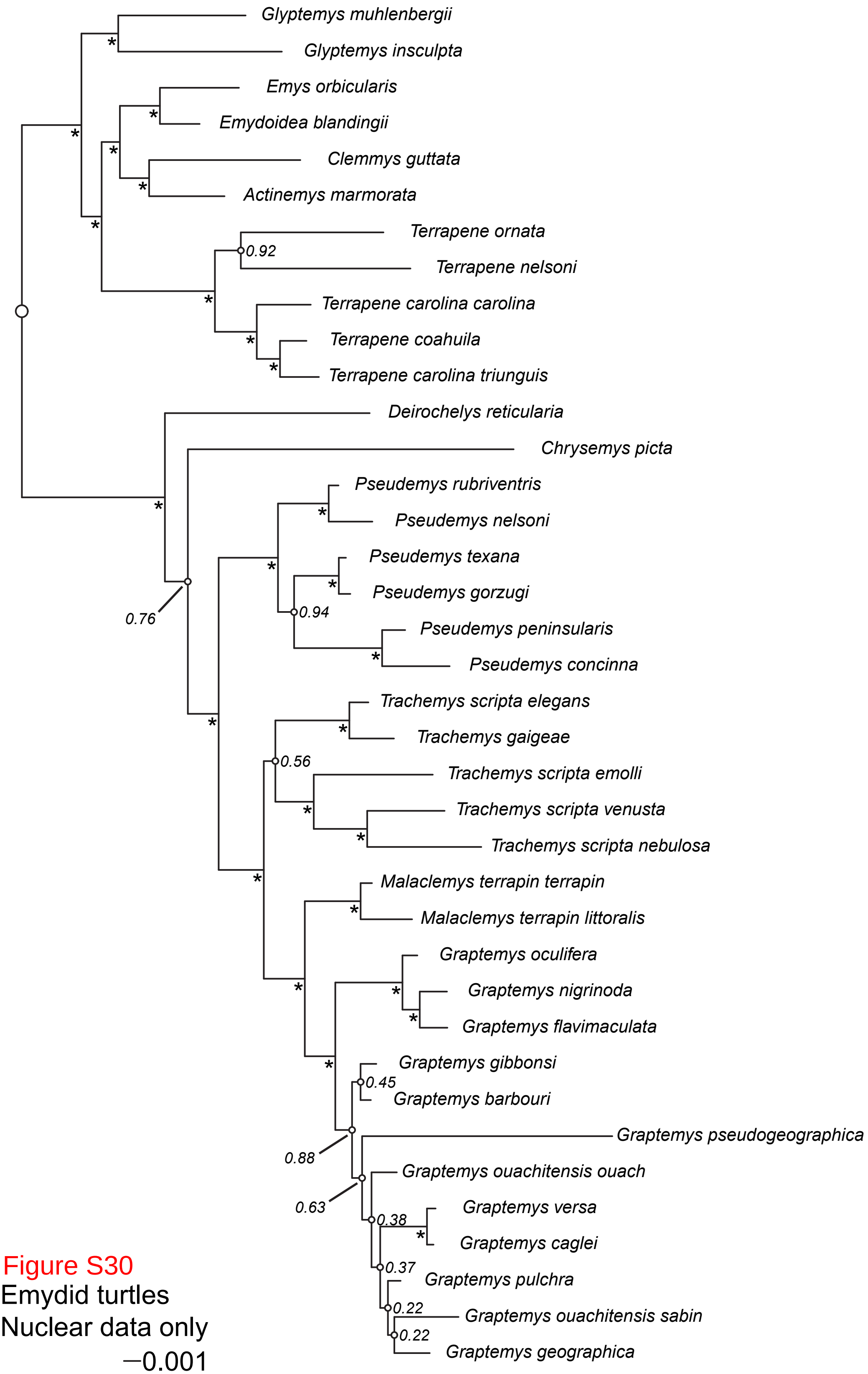

Figure S30
Emydid turtles
Nuclear data only

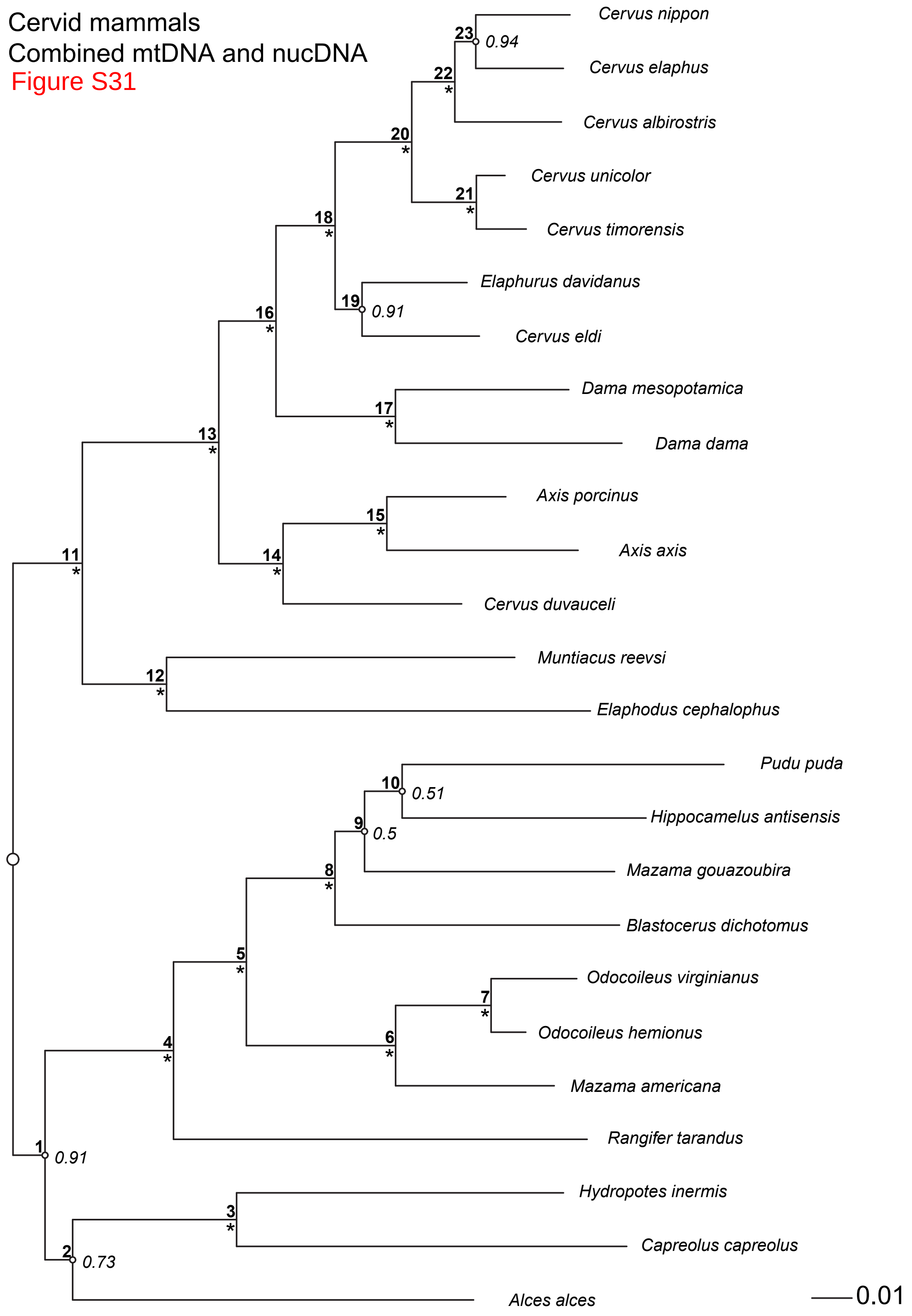
Cervid mammals
Combined mtDNA and nucDNA
Figure S31
Cervus nippon
Cervus elaphus
Cervus albirostris
Cervus unicolor
Cervus timorensis
Elaphurus davidanus
Cervus eldi
Dama mesopotamica
Dama dama
Axis porcinus
Axis axis
Cervus duvauceli
Muntiacus reevsi
Elaphodus cephalophus
Pudu puda
Hippocamelus antisensis
Mazama gouazoubira
Blastocerus dichotomus
Odocoileus virginianus
Odocoileus hemionus
Mazama americana
Rangifer tarandus
Hydropotes inermis
Capreolus capreolus
Alces alces
23
0.94
22
*
20
*
21
*
18
*
19
0.91
16
*
17
*
13
*
15
*
14
*
11
*
12
*
10
0.51
9
0.5
8
*
5
*
7
*
6
*
4
*
1
0.91
3
*
2
0.73
0.01

Cervid mammals
Mitochondrial data only
Figure S32

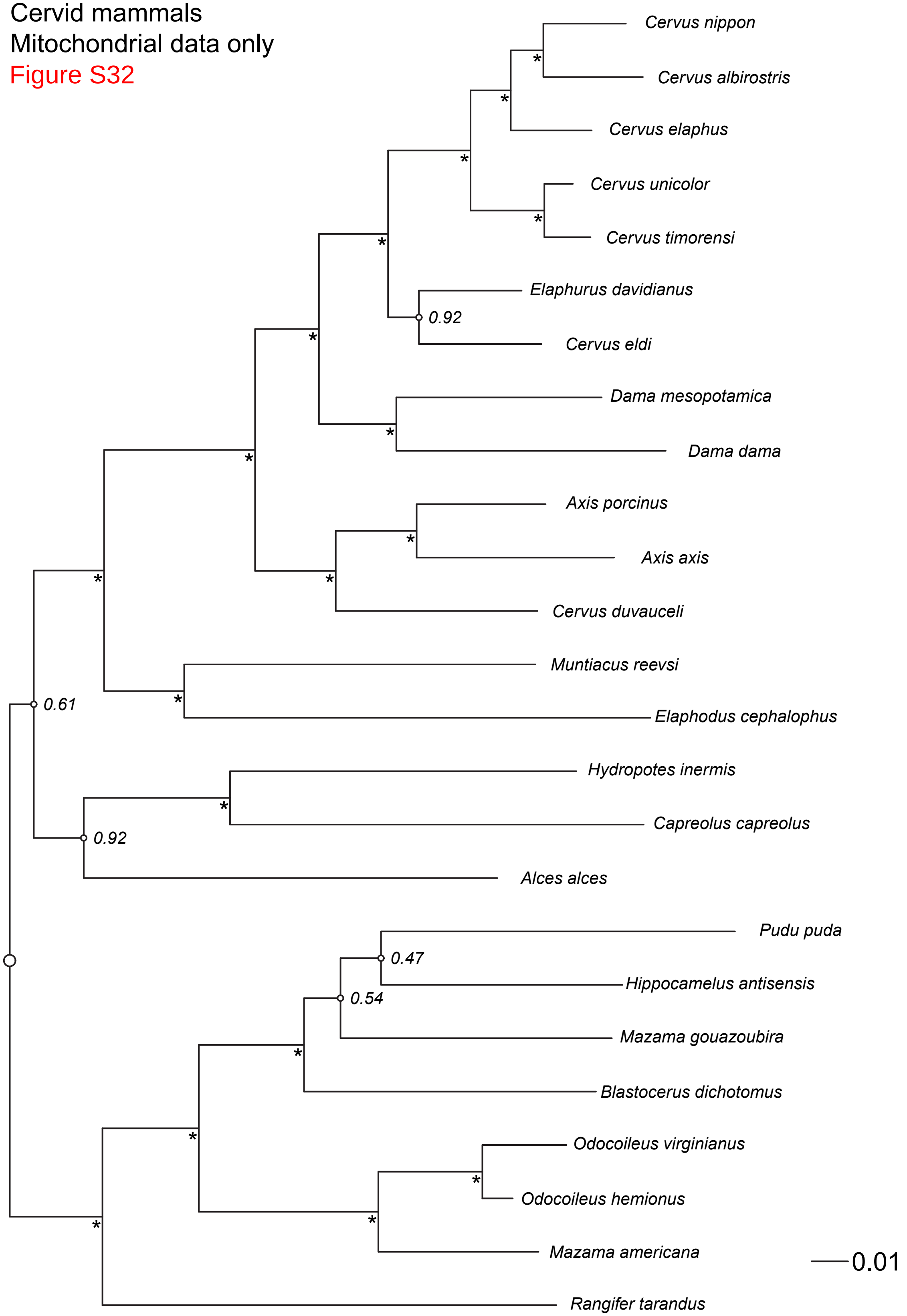

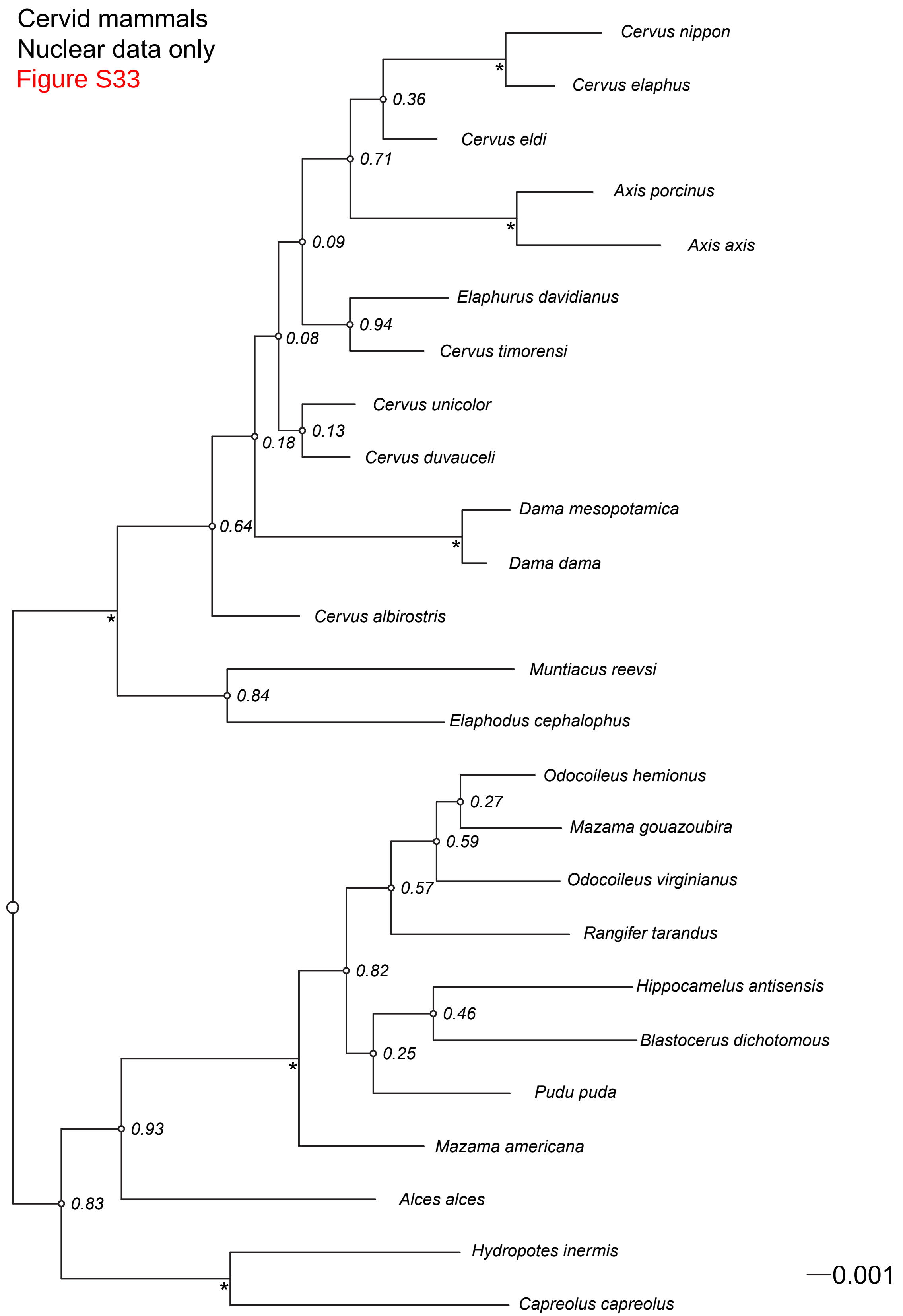
Cervid mammals
Nuclear data only
Figure S33
Cervus nippon
*
Cervus elaphus
0.36
Cervus eldi
0.71
Axis porcinus
*
Axis axis
0.09
Elaphurus davidianus
0.94
0.08
Cervus timorensi
Cervus unicolor
0.13
0.18
Cervus duvauceli
Dama mesopotamica
0.64
*
Dama dama
*
Cervus albirostris
Muntiacus reevsi
0.84
Elaphodus cephalophus
Odocoileus hemionus
0.27
Mazama gouazoubira
0.59
Odocoileus virginianus
0.57
Rangifer tarandus
0.82
Hippocamelus antisensis
0.46
Blastocerus dichotomous
0.25
*
Pudu puda
0.93
Mazama americana
Alces alces
0.83
Hydropotes inermis
*
Capreolus capreolus
0.001

Murid rodents (Philippines)
Combined mtDNA and nucDNA

Figure S34

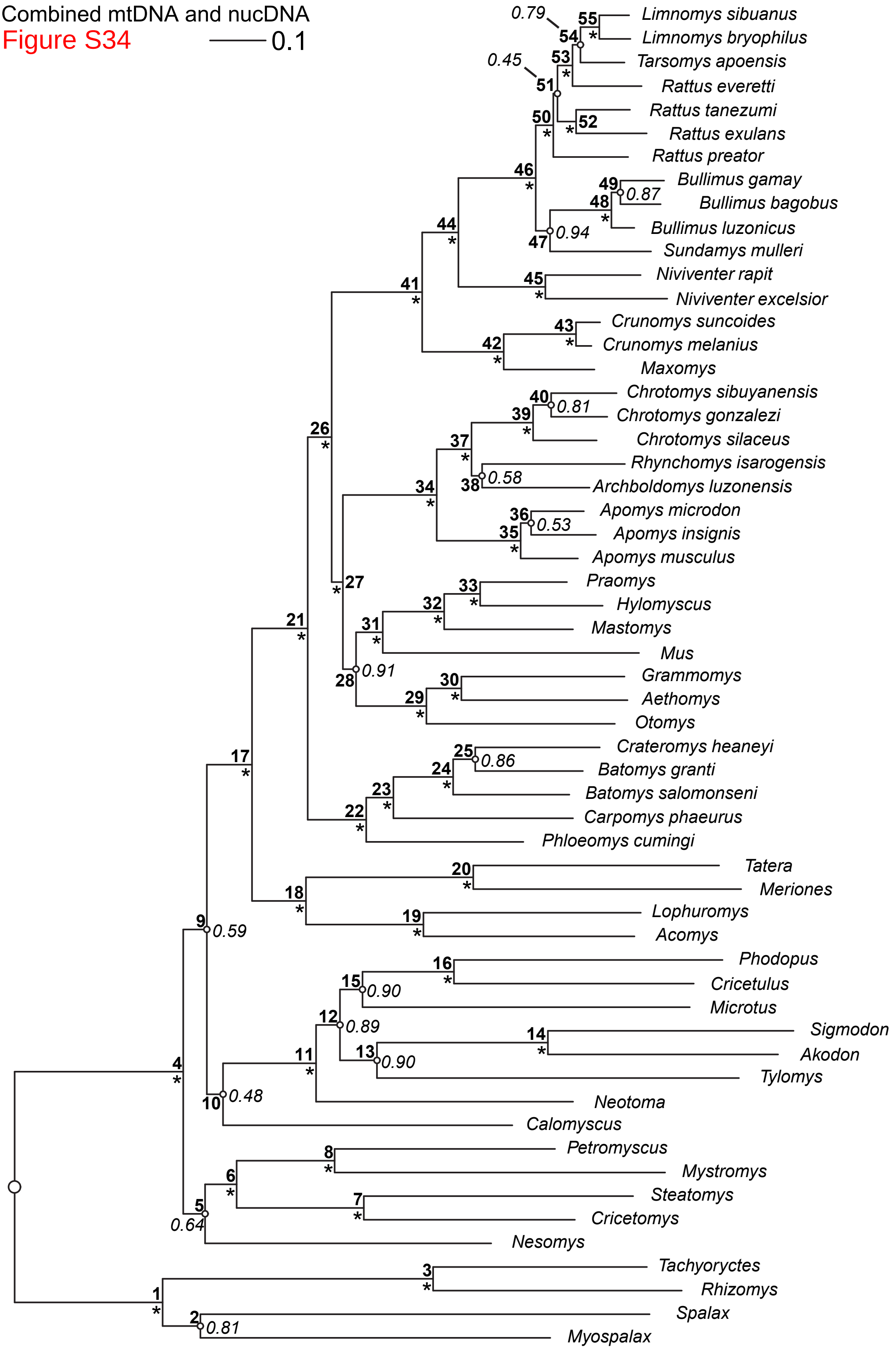

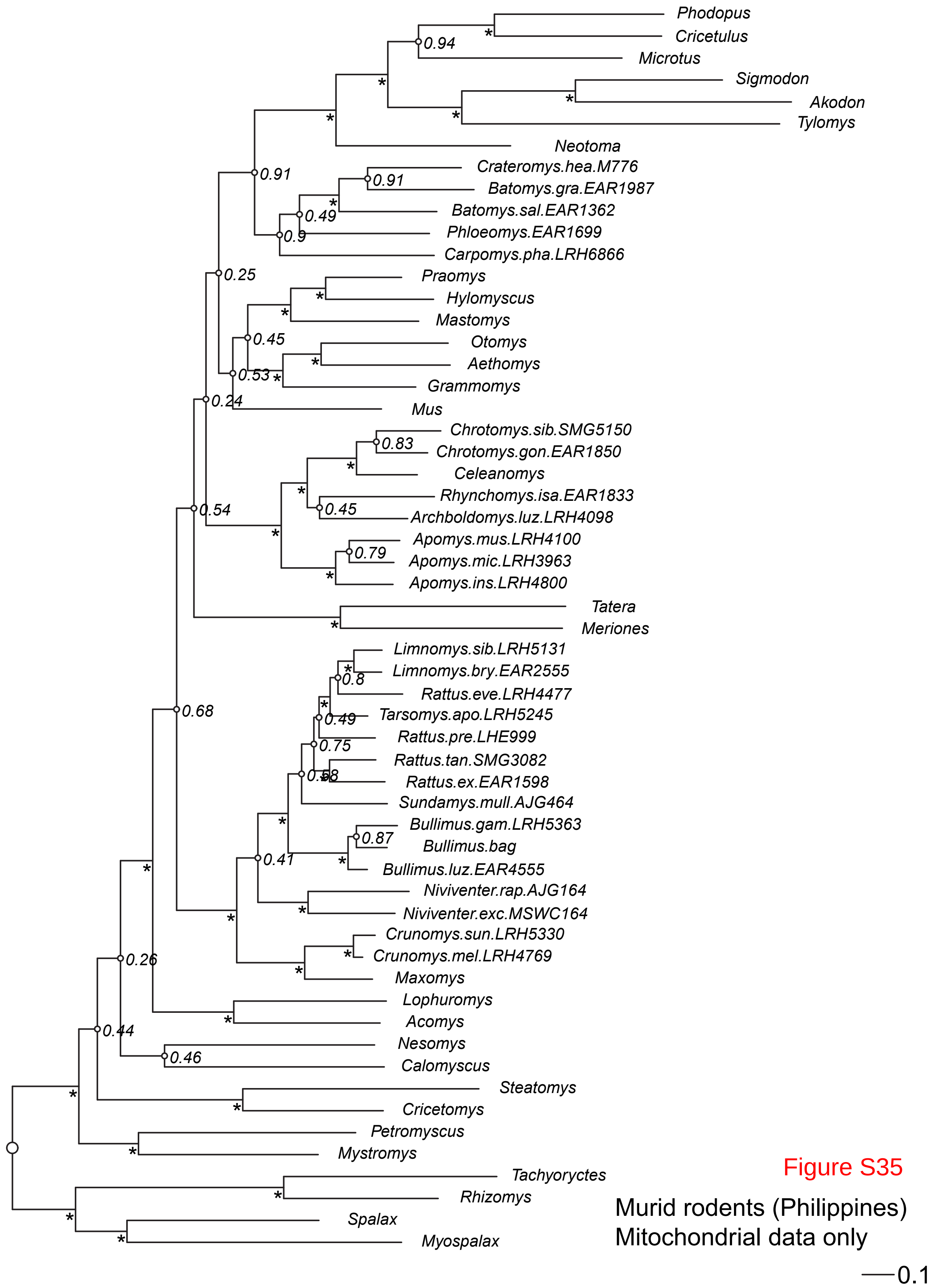

Figure S35

Murid rodents (Philippines)
Mitochondrial data only

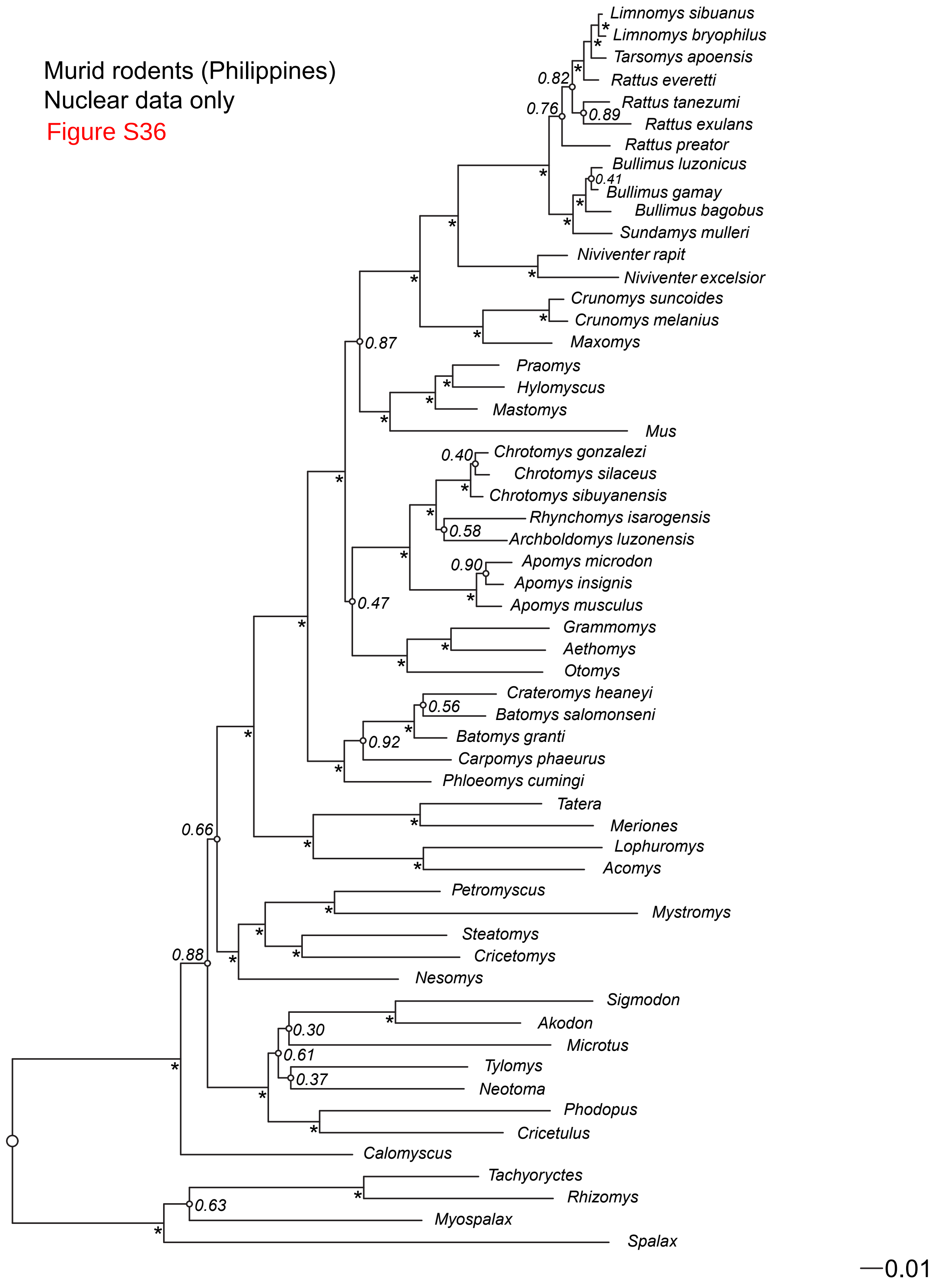
Murid rodents (Philippines)
Nuclear data only
Figure S36
Limnomys sibuanus
Limnomys bryophilus
Tarsomys apoensis
Rattus everetti
Rattus tanezumi
Rattus exulans
Rattus preator
Bullimus luzonicus
Bullimus gamay
Bullimus bagobus
Sundamys mulleri
Niviventer rapit
Niviventer excelsior
Crunomys suncoides
Crunomys melanius
Maxomys
Praomys
Hylomyscus
Mastomys
Mus
Chrotomys gonzalezi
Chrotomys silaceus
Chrotomys sibuyanensis
Rhynchomys isarogensis
Archboldomys luzonensis
Apomys microdon
Apomys insignis
Apomys musculus
Grammomys
Aethomys
Otomys
Crateromys heaneyi
Batomys salomonseni
Batomys granti
Carpomys phaeurus
Phloeomys cumingi
Tatera
Meriones
Lophuromys
Acomys
Petromyscus
Mystromys
Steatomys
Cricetomys
Nesomys
Sigmodon
Akodon
Microtus
Tylomys
Neotoma
Phodopus
Cricetulus
Calomyscus
Tachyoryctes
Rhizomys
Myospalax
Spalax
0.82
0.76
0.89
0.41
0.87
0.40
0.58
0.90
0.47
0.56
0.92
0.66
0.88
0.30
0.61
0.37
0.63
0.01

Murid Rodents (Sahul)
Combined mtDNA and nucDNA
Figure S37

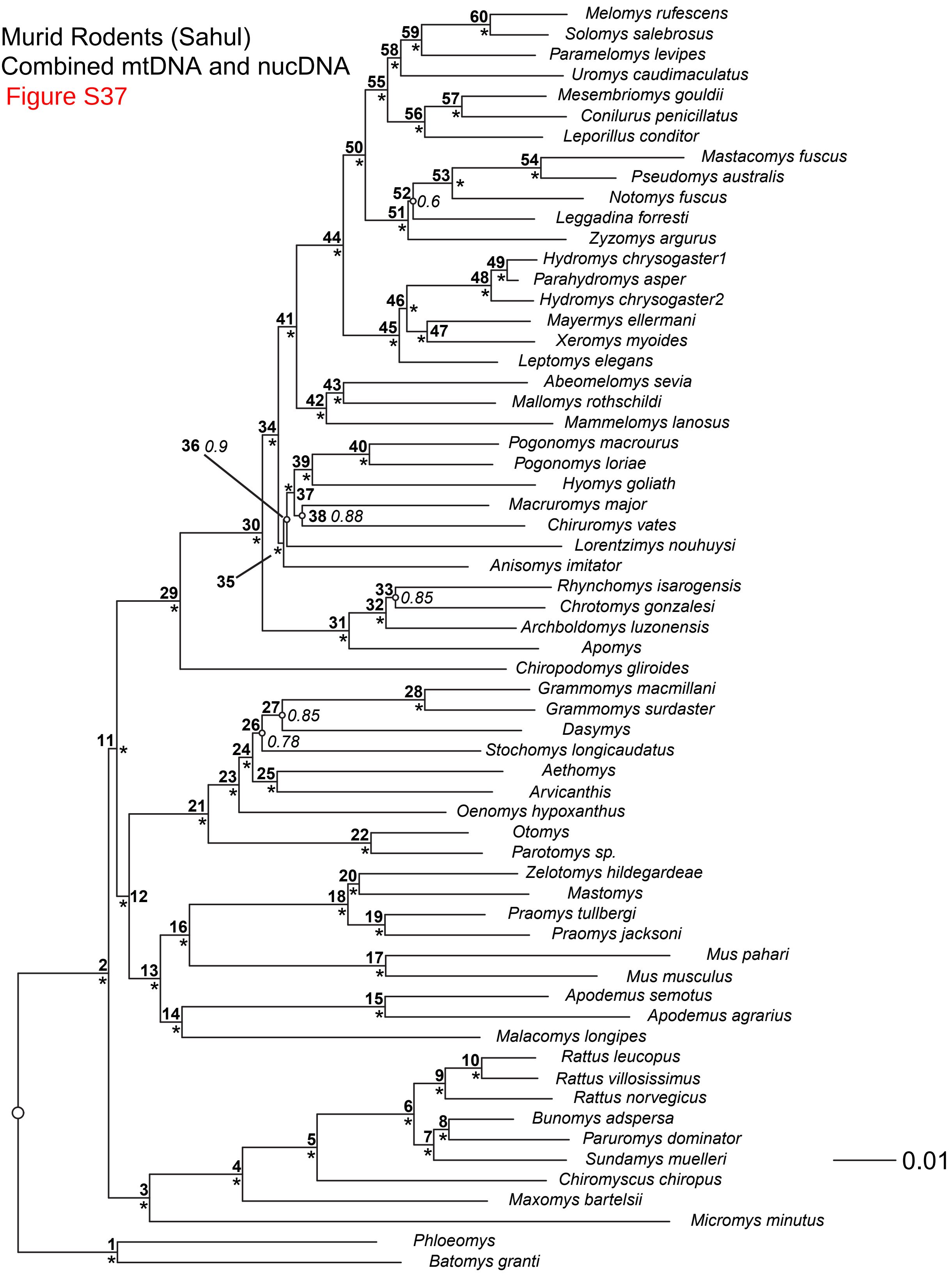

Murid rodents (Sahul)
Mitochondrial data only
Figure S38

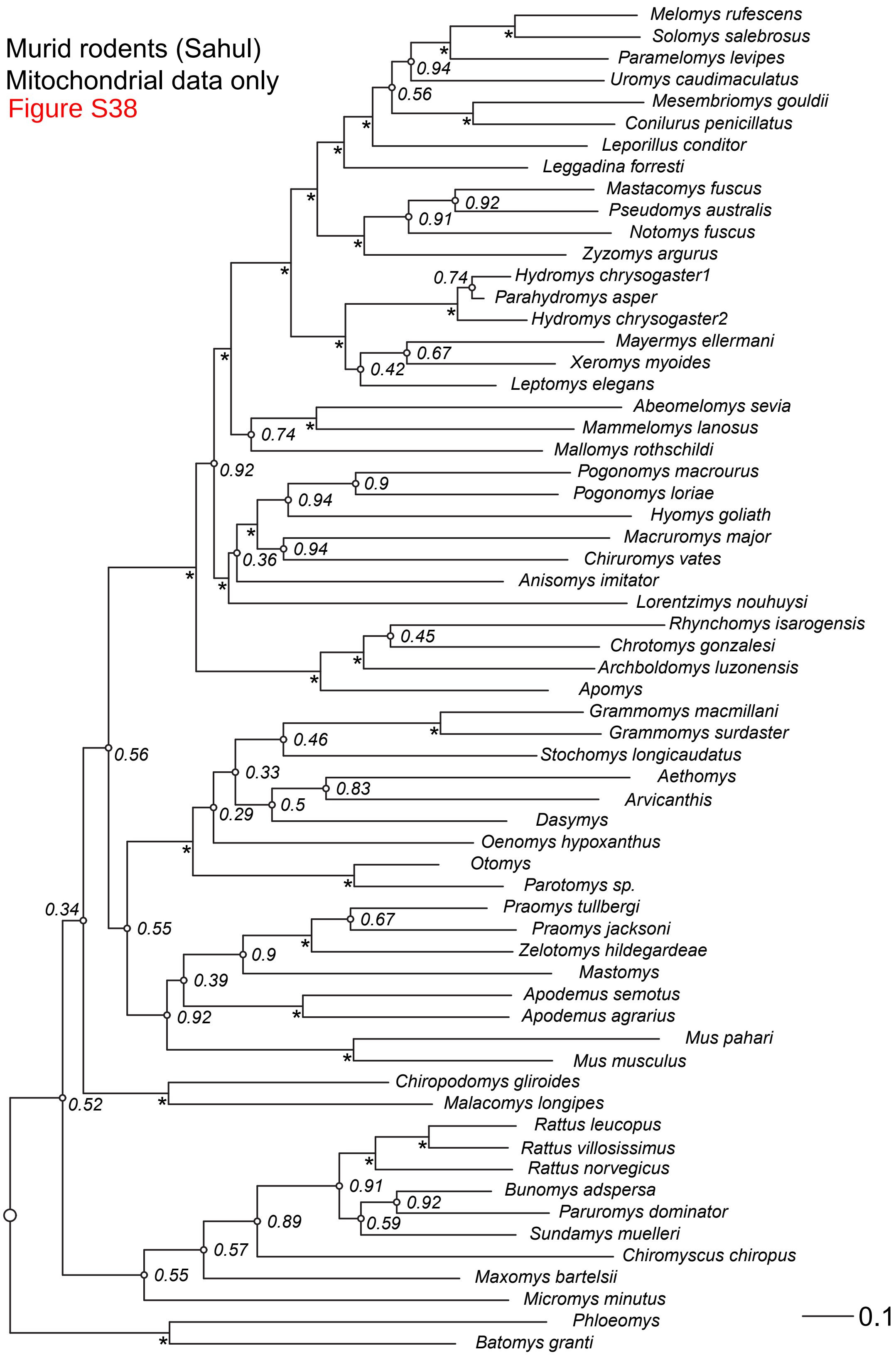

Murid rodents (Sahul)
Nuclear data only
Figure S39

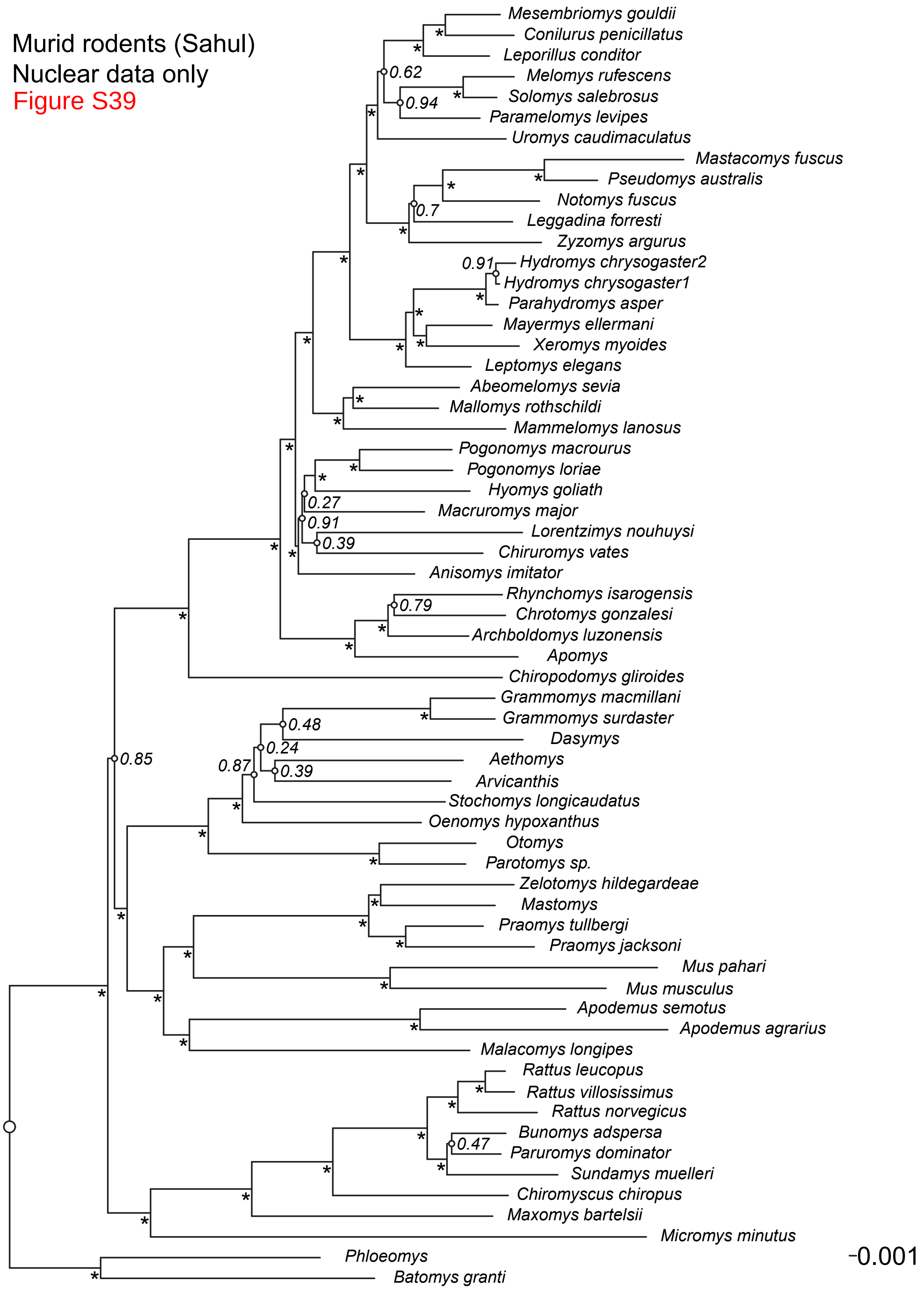

Supplement: Additional file 2 — Phylogenies for each vertebrate clade. Supplemental figures S1 through S39. Phylogenies for each sampled vertebrate clade based on a partitioned Bayesian analysis of combined data (first tree), mitochondrial DNA (second tree), and nuclear DNA (third tree). An asterisk next to a node indicates strong support, (Pp) ≥ 0.95. Small white circles on a node indicate (Pp) < 0.95 and these values are listed. Integers next to each node in the combined tree correspond to clade numbers used in analyses. The outgroup taxa are excluded for all groups to facilitate presentation of branch lengths, and the root is indicated with an open circle. Figures S1, S2, S3: balistid fish; Figures S4, S5, S6: scarine fish; Figures S7, S8, S9: hemiphractid frogs; Figures S10, S11, S12: hylid frogs; Figures S13, S14, S15: phrynosomatid lizards; Figures S16, S17, S18: alcid birds; Figures S19, S20, S21: caprimulgid birds; Figures S22, S23, S24: cotingid birds; Figures S25, S26, S27: dicaeid birds; Figures S28, S29, S30: emydid turtles; Figures S31, S32, S33: cervid mammals; Figures S34, S35, S36: murid rodents (Philippines); Figures S37, S38, S39: murid rodents (Sahul = Australia-New Guinea). PDF file. [file 1471-2148-11-300-S2.PDF]
