# Supplementary material for: What are the consequences of combining nuclear and mitochondrial data for phylogenetic analysis? Lessons from Plethodon salamanders and 13 other vertebrate clades
Source: BMC Evol Biol. 2011 Oct 13;11:300. doi: 10.1186/1471-2148-11-300 (PMC3203092; doi:10.1186/1471-2148-11-300)
Supplement: Additional file 3 — Plethodon specimens used in this study. New data for this study were collected from the following specimens of Plethodon and outgroups from the listed localities. Whenever possible, existing data were matched by individual to the new data. Numbers following species names correspond to specimen numbers used in the figures. Acronyms for voucher specimens are as follows: AC = Andy Coleman field series; APPSU = Appalachian State University collection; DBS = Don B. Shepard field series; DWW = David W. Weisrock field series; JB = Joseph Bernardo field series, JJW = John J. Wiens field series; RH = Richard Highton field series; RMB = Ronald M. Bonett specimen number; RWV = R. Wayne VanDevender field series; SDF = San Diego Natural History Museum field series; UTA A = University of Texas at Arlington amphibian collection; UABC = Universidad Autonoma de Baja California. PDF file. [file 1471-2148-11-300-S3.PDF]

Additional file 3 – *Plethodon* specimens used in this study

New data for this study were collected from the following specimens of *Plethodon* and outgroups from the listed localities. Whenever possible, existing data was matched by individual to the new data. Numbers following species names correspond to specimen numbers used in the figures. Acronyms for voucher specimens are as follows: AC = Andy Coleman field series; APPSU = Appalachian State University collection; DBS = Don B. Shepard field series; DWW = David W. Weisrock field series; JB = Joseph Bernardo field series (to be deposited in M. L. Bean Museum at Brigham Young University), JJW = John J. Wiens field series (to be deposited at U.S. National Museum); RH = Richard Highton field series; RMB = Ronald M. Bonett specimen number; RWV = R. Wayne VanDevender field series; SDF = San Diego Natural History Museum field series; UTA A = University of Texas at Arlington amphibian collection; UABC = Universidad Autonoma de Baja California.

Species	Voucher	State	County	Locality
Aneides lugubris	SDF 2655	CA		Vivarium (no voucher)
Desmognathus carolinensis-1	JJW 1688	NC	Madison County	Cherokee National Forest; near head of Betty Place Trail, along West Branch of Shut-in Creek; off of Upper Shut-In Road south of Highway 25/70 , west of Hot Springs: 35°51.694 N; 82°54.574 W
Desmognathus	JJW 1689	NC	Madison County	Cherokee National Forest; near head of Betty Place Trail, along West Branch of Shut-in Creek;

carolinensis-2				off of Upper Shut-In Road south of Highway 25/70 , west of Hot Springs: 35°51.694 N; 82°54.574 W
Eurycea bislineata-1	JJW 1832	NY	Rockland	Orangeburg, Tackamack Town Park, Clausland Mountain Road, ca. 1 mile west of Route 5: 41.0626 N; 73.94008 W
Eurycea bislineata-2	JJW 1837	NJ	Warren	Delaware Water Gap; Worthington State Forest, Dunnfield Creek Natural Area at Highway 80 near Pennsylvania border N 40 58.328 W 075 07.542
Ensatina eschscholtzii	SDF 1861	CA	Monterey	No voucher
Ensatina klauberi	UABC 1434, SDF 1167			Baja California, Mexico
Plethodon albagula	RMB 2041	AR	Garland	Tributary of Cearley Creek, ~4 mi west of Royal on Highway 270
Plethodon amplus	UTA A-56963	NC	Henderson	Grant Mountain

Plethodon angusticlavius	RWV S58A	AR	Newton	RMB locality #9, 2002
Plethodon aureolus-1	JJW 1759	TN	Monroe	Cherokee National Forest, Grassy Gap on Sassafras Ridge, on Highway 165
Plethodon aureolus-2	JJW 1762	TN	Monroe	Cherokee National Forest, Grassy Gap on Sassafras Ridge, on Highway 165
Plethodon caddoensis	UTA A-56964	AR	Montgomery	Caddo Mountains, ca. 1.5 miles W of Black Springs on SH 8
Plethodon chatahoochee	JJW 1767	GA	Union	Chattahoochee National Forest, near Blairsville, Gumlog Road; 2515 Old Gumlog Spur
Plethodon cheoah	JJW 1730	NC	Graham	Nantahala National Forest; Stecoah Gap, off Highway 143 NE Robbinsville
Plethodon chlorobryonis	UTA A-56966			
Plethodon cinereus-1	APPSU 23844	NC	Caldwell	Grandfather Mountain, along US Highway 221 at Dixon Creek
Plethodon cinereus-2	JJW 1081	NY	Suffolk	Manorville, on Highway 111 N of Long Island Expressway (Interstate 495) exit 70, behind Manorville Community Church
Plethodon	JJW 1641	VA	Washingt	Mount Rogers National Recreation Area, vicinity

cinereus-3			on	of Beartree Campground
Plethodon cinereus-4	PH 9	VA	Madison	Shenandoah National Park: Hawksbill Mountain (38° 33’ 20” N; 78° 23’ 11” W)
Plethodon cylindraceus	UTA A-56967	NC	Buncombe	Shumont Mt. Road, 1 mile E. of NC 9
Plethodon dorsalis	APPSU 24549	KY	Bullitt	Coll. by John McGregor
Plethodon electromorphus	RH 54087	PA	Beaver	near Frankfort Springs, 49º 29’ 50” N; 80º 25’ 52” W
Plethodon elongatus	RH 75-29	OR		
Plethodon fourchensi-2	DBS 485	AR	Polk	Fourche Mountains, Foran Gap
Plethodon glutinosus-1	APPSU 10209	GA	Dade	Trenton, Highway 136, 1.3 mi N of Interstate Highway 59
Plethodon glutinosus-2	JJW 1704	TN	Cocke	Cherokee National Forest; road to Meadow Creek Firetower off Paint Creek Road, Burnettt Creek
Plethodon	JJW 1809	NY	Rockland	Orangeburg, Highway 9W; ~1 mi north of road

glutinosus-3				to Snedens Landing
Plethodon glutinosus-4	UTA A-56969	IN	Monroe	Kerr Creek, off of Kerry Drive and Route 46, Bloomington
Plethodon grobmani-1	AC-02-41	AL	Butler	Greenville
Plethodon grobmani-2	UTA A-56970	FL	Jackson	Spring Creek, E. of Marianna
Plethodon hoffmani	JB 201-05	MD	Allegheny	Jennings Run
Plethodon hubrichti	JJW 1782	VA	Bedford	Jefferson National Forest; Blue Ridge Parkway, just west of Peaks of Otter Campground, mountain slope across from Headforemost Mountain Viewpoint
Plethodon idahoensis	UTA A56971	ID	Idaho	Selway River at O'Hara
Plethodon jordani	JB 201-73-01	NC	Haywood	Great Smoky Mountains: Mount Sterling
Plethodon kentucki	RH 66693	KY	Bell	Log Mountain
Plethodon kiamichi	RH 58676	OK	LeFlore	34º 36' 55" N; 94º 29' 50" W

Plethodon kisatchie	RMB 2990	LA	Grant	Kisatchie National Forest, small unmarked road off SR 500 between Georgetown and Zion, ca. 5 miles W junction of SH 165 and SR 500
Plethodon longicrus	APPSU 24566	NC	Henderson	Bearwallow Mountain, forest service road to fire tower; 35º 27.77' N; 82º 22.19' W
Plethodon meridianus	APPSU 24811	NC	Burke	South Mountain State Park; Waterfall Trail near park headquarters
Plethodon metcalfi	RH 76539	NC	Jackson	Sandy Gap; 35º 12’ 07” N; 83º 05’ 21” W; 1274 m; Blue Ridge Isolate
Plethodon mississippi	APPSU 10214	AL	Tuscaloosa	Tuscaloosa, Lake Nicol at Lake Nicol Road
Plethodon montanus	DWW 223	NC	Watagua	Grady Winkler Road, 36º 16.028’ N; 81º 41.517’ W
Plethodon nettingi	RH 66737	WV	Pocohontas	Gaudineer Knob
Plethodon oconaluftee	JJW 1626	TN	Monroe	Nantahala National Forest: Sassafras Ridge, Grassy Gap off Highway 143
Plethodon ocmulgee	RH 77028	GA	Dodge	32º 05’ 38” N; 82º 53’ 35” W
Plethodon ouachitae	DBS 405	OK	Le Flore	Winding Stair Mountain
Plethodon petraeus	RWV S21-B	GA	Walker	Pigeon Mountain

Plethodon punctatus	JJW 1796	W V	Pendleton	Shenandoah Mountain, Hall Spring Hunters Access Road off of Route 33, 0.2 mi (by road) from Virginia line
Plethodon richmondi	RMB 2790	KY	Owen	US 127 at Eagle Creek
Plethodon savananah	RH 70361	GA	Richmond	ENE of Hephzibah just NW intersection of U.S. Route 25 (Peach Orchard Road) and Rheney Road
Plethodon sequoyah	APPSU 24547	OK	McCurtai n	Beaver's Bend State Park
Plethodon serratus	JJW 1765	TN	Monroe	Cherokee National Forest, Grassy Gap on Sassafras Ridge, on Highway 165
Plethodon shenandoa h-1	PS H10	VA	Page	Hawksbill Mountain, Shenandoah National Park
Plethodon shenandoa h-2	PS H8	VA	Page	Hawksbill Mountain, Shenandoah National Park
Plethodon shermani	DWW 25	NC	Clay	Tusquitee Isolate; 35º 9.205' N; 83º 45.067' W
Plethodon teyahalee	RH 55224	NC	Macon	35° 06' 20" N; 83° 17' 05" W
Plethodon	UTA A-	W	Lewis	Tributary of Tilton River

vandykei	56609	A		
Plethodon variolatus	UTA A-56960	SC	Berkeley	Francis Marion National Forest, McConnel Landing, along Santee River
Plethodon vehiculum	UTA A-56610	WA	Lewis	Tributary of Tilton River
Plethodon ventralis	UTA A-56974	KY	Pulaski	Hale Cave
Plethodon virginia	JJW 1800	WV	Pendleton	Hall Spring Hunters Access Road, Shenandoah Mountain, Route 33, 0.2 mi (by road) from Virginia line
Plethodon websteri	APPSU 24208	SC	McCormick	Stevens Creek at State Secondary Route 88 close to Clarks Hill
Plethodon wehrlei	RWV S18	NC	Allegheny	Saddle Mountain
Plethodon welleri	JB 202-19	VA	Grayson	Whitetop Mountain
Plethodon yonahlossee-1	JJW 1616	VA	Washington	Mount Rogers National Recreation Area: vicinity of Beartree Campground, Lum Trail
Plethodon yonahlossee-2	RMB 2458	NC	Watauga	0.7 mi E junction with Shulls Mill Road
