# Supplementary material for: What are the consequences of combining nuclear and mitochondrial data for phylogenetic analysis? Lessons from Plethodon salamanders and 13 other vertebrate clades
Source: BMC Evol Biol. 2011 Oct 13;11:300. doi: 10.1186/1471-2148-11-300 (PMC3203092; doi:10.1186/1471-2148-11-300)
Supplement: Additional file 4 — GenBank accession numbers for new data collected for this study. Sequences that are less than 200 bp (denoted by *****) are not accepted by GenBank and are available from M.C. Fisher-Reid upon request. Dashes (-) indicate that the sequence was not collected for that individual at that locus. [file 1471-2148-11-300-S4.PDF]

Additional File 4 - GenBank accession numbers for new data collected for this study. Sequences that are less than 200 bp (denoted by *****) are not accepted by GenBank and are available from M.C. Fisher-Reid upon request. Dashes (-) indicate that the sequence was not collected for that individual at that locus.

Species	Voucher	GAP-D	RHO	RPL-12	ILF3	Mlc2a
Aneides lugubris	SDF 2655	-	JN798356	JN798416	-	*****
Desmognathus carolinensis-1	JJW 1688	-	JN798357	-	-	JN798304
Desmognathus carolinensis-2	JJW 1689	-	JN798358	-	-	JN798305
Eurycea bislineata-1	JJW 1832	-	JN798359	JN798417	-	-
Eurycea bislineata-2	JJW 1837	-	JN798360	JN798418	-	-
Ensatina eschscholtzii	SDF 1861	-	JN798361	JN798419	-	-
Ensatina klauberi	UABC 1434	-	JN798362	-	-	JN798306

Plethodon albagula	RMB 2041	JN798216	JN798363	JN798420	JN798264	JN798307
Plethodon amplus	UTA A-56963	JN798217	JN798364	JN798421	JN798265	JN798308
Plethodon angusticlavius	RWV S58A	JN798218	-	-	-	-
Plethodon aureolus-1	JJW 1759	JN798219	JN798365	JN798422	JN798266	JN798309
Plethodon aureolus-2	JJW 1762	JN798220	JN798366	JN798423	JN798267	JN798310
Plethodon caddoensis	UTA A-56964	JN798221	JN798367	JN798424	JN798268	JN798311
Plethodon chatahoochee	JJW 1767	JN798222	JN798368	JN798425	JN798269	JN798312
Plethodon cheoah	JJW 1730	JN798223	JN798369	JN798426	JN798270	JN798313
Plethodon chlorobryonis	UTA A-56966	JN798224	JN798370	JN798427	JN798271	JN798314
Plethodon cinereus-1	APPSU 23844	JN798225	JN798371	-	-	JN798315

Plethodon cinereus-2	JJW 1081	JN798226	JN798372	-	-	-
Plethodon cinereus-3	JJW 1641	JN798227	JN798373	JN798428	-	JN798316
Plethodon cinereus-4	PH 9	JN798228	JN798374	-	-	JN798317
Plethodon cylindraceus	UTA A-56967	JN798229	JN798375	JN798429	JN798272	JN798318
Plethodon dorsalis	APPSU 24549	-	JN798376	JN798430	JN798273	JN798319
Plethodon electromorphus	RH 54087	JN798230	JN798377	-	-	JN798320
Plethodon elongatus	RH 75-29	JN798231	-	-	-	-
Plethodon fourchensis	DBS 485	JN798232	-	JN798431	JN798274	JN798321
Plethodon glutinosus-1	APPSU 10209	-	*****	JN798432	-	JN798322
Plethodon glutinosus-2	JJW 1704	JN798233	JN798378	-	JN798275	JN798323
Plethodon glutinosus-3	JJW 1809	JN798234	JN798379	JN798433	-	JN798324

Plethodon glutinosus-4	UTA A-56969	JN798235	JN798380	JN798434	-	-
Plethodon grobmani-1	AC-02-41	JN798236	JN798381	JN798435	JN798276	JN798325
Plethodon grobmani-2	UTA A-56970	JN798237	JN798382	JN798436	JN798277	JN798326
Plethodon hoffmani	JB 201-05	JN798238	JN798383	-	-	JN798327
Plethodon hubrichti	JJW 1782	JN798239	JN798384	JN798437	-	JN798328
Plethodon idahoensis	UTA A56971	-	-	-	-	JN798329
Plethodon jordani	JB 201-73-01	-	JN798385	JN798438	JN798278	JN798330
Plethodon kentucki	RH 66693	JN798240	JN798386	JN798439	JN798279	JN798331
Plethodon kiamichi	RH 58676	JN798241	JN798387	JN798440	JN798280	JN798332
Plethodon kisatchie	RMB 2990	JN798242	JN798388	JN798441	JN798281	JN798333
Plethodon longicrus	APPSU 24566	JN798243	JN798389	JN798442	JN798282	JN798334
Plethodon	APPSU	JN798244	JN798390	JN798443	JN798283	JN798335

meridianus	24811					
Plethodon metcalfi	RH 76539	JN798245	*****	JN798444	JN798284	-
Plethodon mississippi	APPSU 10214	JN798246	JN798391	JN798445	JN798285	-
Plethodon montanus	DWW 223	JN798247	JN798392	JN798446	JN798286	JN798336
Plethodon nettingi	RH 66737	JN798248	JN798393	JN798447	-	-
Plethodon oconaluftee	JJW 1626	JN798249	JN798394	JN798448	JN798287	JN798337
Plethodon ocmulgee	RH 77028	JN798250	JN798395	JN798449	JN798288	JN798338
Plethodon ouachitae	DBS 405	JN798251	JN798396	JN798450	JN798289	-
Plethodon petraeus	RWV S21-B	JN798252	JN798397	JN798451	JN798290	JN798339
Plethodon punctatus	JJW 1796	JN798253	JN798398	-	JN798291	*****
Plethodon richmondi	RMB 2790	JN798254	JN798399	JN798452	-	JN798340
Plethodon savananah	RH 70361	JN798255	JN798400	JN798453	JN798292	JN798341

Plethodon sequoyah	APPSU 24547	-	JN798401	JN798454	JN798293	JN798342
Plethodon serratus	JJW 1765	JN798256	JN798402	-	-	JN798343
Plethodon shenandoah-1	PS H10	JN798257	JN798403	JN798455	-	JN798344
Plethodon shenandoah-2	PS H8	JN798258	JN798404	-	-	JN798345
Plethodon shermani	DWW 25	JN798259	JN798405	JN798456	JN798294	JN798346
Plethodon teyahalee	RH 55224	-	JN798406	JN798457	JN798295	JN798347
Plethodon vandykei	UTA A-56609	JN798260	-	-	-	*****
Plethodon variolatus	UTA A-56960	-	JN798407	JN798458	JN798296	JN798348
Plethodon vehiculum	UTA A-56610	-	JN798408	JN798459	-	JN798349
Plethodon ventralis	UTA A-56974	JN798261	JN798409	-	JN798297	JN798350
Plethodon	JJW 1800	-	JN798410	JN798460	JN798298	JN798351

virginia						
Plethodon websteri	APPSU 24208	-	JN798411	-	JN798299	JN798352
Plethodon wehrlei	RWV S18	-	JN798412	-	JN798300	JN798353
Plethodon welleri	JB 202-19	JN798262	JN798413	JN798461	JN798301	-
Plethodon yonahlossee-1	JJW 1616	JN798263	JN798414	JN798462	JN798302	JN798354
Plethodon yonahlossee-2	RMB 2458	-	JN798415	JN798463	JN798303	JN798355
