# Supplementary material for: What are the consequences of combining nuclear and mitochondrial data for phylogenetic analysis? Lessons from Plethodon salamanders and 13 other vertebrate clades
Source: BMC Evol Biol. 2011 Oct 13;11:300. doi: 10.1186/1471-2148-11-300 (PMC3203092; doi:10.1186/1471-2148-11-300)
Supplement: Additional file 5 — Primer sequences for new nuclear genes. Primers for five nuclear genes (RHO, RPL12, Mlc2a, ILF3, GAPD) from which new sequence data for Plethodon were collected for this study. Forward primers are indicated by "F" in the primer name, and reverse primers are indicated by "R" in the primer name. PDF file. [file 1471-2148-11-300-S5.PDF]

Additional File 5 – Primer sequences for new nuclear genes

Primers for five nuclear genes (RHO, RPL12, Mlc2a, ILF3, GAPD) from which new sequence data for *Plethodon* were collected for this study. Forward primers are indicated by "F" in the primer name, and reverse primers are indicated by "R" in the primer name.

Primer Name	Primer Sequence (5'-3')	Gene Name	Source
RHO3F	CTGAAGCCTGAGGTCAACAATGA	Rhodopsin Intron 3 (RHO)	K.H. Kozak (pers. comm.)
RHO4R	GTGACCTCTTTCTCAGCCTTCTG		
C31/32F	ATTCCACTGCACCGCTATTGAT	60s ribosomal protein L12 (RPL12)	T. Devitt (pers. comm.)
C31/32R	CCCAAGTTTGACCCTACAGAGAT		
C3/4F	ATGCGTGTGAATTCCACATAATTG	Myosin light chain 2 mRNA (Mlc2a)	T. Devitt (pers. comm.)
C3/4R	GAAGAACCCAACTGATGAATACCT		
C93/94F	GATTTCAATCCATTTGCTCTTGC	interleukin enhancer binding factor 3 (ILF3)	T. Devitt (pers. comm.)
C93/94R	AGGATAAGCCCACCGTTACACTATT		
GapDL890	ACCTTTAATGCGGGTGCTGG	glyceraldehyde-3-	Friesen et al. 1997

(F)	CATTGC	phosphate
GapDH950	CATCAAGTCCACAACACGGT	dehydrogenase
(R)	TGCTGTA	(GAPD)
