# Supplementary material for: What are the consequences of combining nuclear and mitochondrial data for phylogenetic analysis? Lessons from Plethodon salamanders and 13 other vertebrate clades
Source: BMC Evol Biol. 2011 Oct 13;11:300. doi: 10.1186/1471-2148-11-300 (PMC3203092; doi:10.1186/1471-2148-11-300)
Supplement: Additional file 6 — GenBank accession numbers for previously published data used in this study. Sources include: RAG-1, TPI, ND4 and Cyt-b data from Wiens et al. 2006. POMC and BDNF data from Vieites et al. 2007; Bonnet et al. 2009. ND2 data from Kozak et al. 2005; Weisrock et al. 2005; Kozak et al. 2006a; Kozak et al. 2006b. PDF file. [file 1471-2148-11-300-S6.PDF]

Additional File 6 - GenBank accession numbers for previously published data used in this study.

Sources include: RAG-1, TPI, ND4 and Cyt-*b* data from Wiens et al. 2006. POMC and BDNF data from Vieites et al. 2007; Bonnet et al. 2009. ND2 data from Kozak et al. 2005; Weisrock et al. 2005; Kozak et al. 2006a; Kozak et al. 2006b.

Taxon	RAG-1	TPI	POMC	BDNF	ND4	Cyt-*b*	ND2
Aneides flavipunctatus	-	-	-	-	-	-	AY612338
Aneides hardii	-	EF040665	-	-	-	-	-
Aneides lugubris	AY650118	-	EU275847	EU275893	AY691802	AY691758	-
Desmognathus brimleyorum	-	-	EU275819	EU275865	-	-	-
Desmognathus carolinensis -1	-	-	-	-	-	-	AY612368
Desmognathus carolinensis	-		-	-	-	-	AY612369

-2							
Desmognathus folksteri	-	EF040666	-	-	-	-	-
Desmognathus fuscus	-	-	EU275812	EU275858	-	-	-
Desmognathus monticola	AY691698	-	-	-	AY691782	AY691738	-
Desmognathus quadramaculatus	AY650117	-	-	-	AY691783	AY691739	-
Ensatina eschscholtzii-1	AY650119	-	EU275816	EU275862	AY691788	AY691744	-
Ensatina eschscholtzii-2	AY691702		-	-	AY691787	AY691743	-
Eurycea bislineata-1	AY650121	-	EU275815	EU275861	AY528327	AY528402	DQ018409
Eurycea bislineata-2	-	-	-	-	-	-	DQ018392
Eurycea	AY65012	-	-	-	AY52832	AY52840	-

longicauda	1				8	3	
Plethodon albagula	DQ995008	EF040667	-	-	DQ995081	DQ994907	DQ018688
Plethodon amplus	DQ995010	-	-	-	DQ995084	DQ994912	AY874880
Plethodon angusticlavius	DQ995011	EF040668	-	-	DQ995002	DQ994913	DQ018677
Plethodon aureolus-1	DQ995012	-	-	-	DQ995085	DQ994914	AY874997
Plethodon aureolus-2	-	-	-	-	DQ995086	DQ994914	-
Plethodon caddoensis	DQ995013	EF040669	-	-	DQ995087	DQ994916	AY874875
Plethodon chattahoochee	DQ995014	-	-	-	DQ995090	DQ994919	AY875003
Plethodon cheoah	DQ995015	EF040670	-	-	DQ995092	DQ994921	AY875045
Plethodon chlorobryonis	DQ995016	EF040671	-	-	DQ995094	DQ994923	AY875006
Plethodon	AY69170	-	FJ95136	-	AY69178	AY69174	DQ01866

cinereus-1	3		5		9	5	4
Plethodon cinereus-2	DQ995018	-	-	-	DQ995097	DQ994926	-
Plethodon cinereus-3	DQ995019	-	-	-	DQ995098	DQ994927	-
Plethodon cinereus-4	DQ995021	-	-	-	-	-	-
Plethodon cylindraceus	DQ995022	EF040672	-	-	DQ995099	DQ994928	AY875020
Plethodon dorsalis	DQ995023	EF040673	-	-	DQ995003	DQ994930	DQ018678
Plethodon electromorphus	DQ995025	-	-	-	DQ995005	DQ994932	DQ018665
Plethodon elongatus	AY650120	EF040674	EU275836	EU275882	AY691790	AY691746	-
Plethodon fourchensis	DQ995026	-	EU275838	EU275884	DQ995103	DQ994934	DQ018690
Plethodon glutinosus-1	-	EF040675	-	-	DQ995104	DQ994936	-
Plethodon glutinosus-2	-	-	-	-	DQ995107	DQ994939	AY875023

Plethodon glutinosus-3	-	-	-	-	DQ995105	DQ994937	-
Plethodon glutinosus-4	DQ995027	-	-	-	DQ995106	DQ994938	-
Plethodon grobmani-1	DQ995028	EF040676	-	-	DQ995108	DQ994940	DQ018695
Plethodon grobmani-2	-	-	-	-	DQ995109	DQ994941	-
Plethodon hoffmani	DQ995029	-	EU275837	EU275883	DQ995110	DQ994942	DQ018666
Plethodon hubrichti	DQ995030	EF040677	-	-	DQ995112	DQ994944	DQ018668
Plethodon idahoensis	DQ995031	-	-	-	DQ995113	DQ994945	-
Plethodon jordani	DQ995032	EF040678	EU275835	EU275881	DQ995114	DQ994946	AY874891
Plethodon kentucki	DQ995033	-	-	-	DQ995116	DQ994948	AY875027
Plethodon kiamichi	DQ995034	EF040679	-	-	DQ995118	DQ994950	DQ018696
Plethodon kisatchie	DQ995035	-	-	-	DQ995119	DQ994951	DQ018698
Plethodon	DQ99503	-	-	-	DQ99512	DQ99495	-

longicrus	7				2	3	
Plethodon meridianus	DQ995038	-	-	-	DQ995124	DQ994955	AY874898
Plethodon metcalfi	DQ995039	-	-	-	DQ995125	DQ994956	AY874903
Plethodon mississippi	-	-	-	-	DQ995127	DQ994958	DQ018700
Plethodon montanus	DQ995043	EF040680	-	-	DQ995131	DQ994962	AY874990
Plethodon nettingi	DQ995045	-	-	-	DQ995133	DQ994963	DQ018669
Plethodon ocanaluftee	DQ995046	EF040681	-	-	DQ995134	DQ994965	-
Plethodon ocmulgee	DQ995048	EF040682	-	-	DQ995137	DQ994968	DQ018702
Plethodon ouachitae	AY691704	-	EU275831	EU275877	DQ995140	DQ994971	DQ018703
Plethodon petraeus	DQ995049	EF040683	-	-	DQ995142	DQ994973	DQ018704
Plethodon punctatus	DQ995050	EF040684	-	-	DQ995143	DQ994974	DQ018685
Plethodon richmondi	DQ995053	EF040685	-	-	DQ995146	DQ994977	DQ018670

Plethodon savannah	DQ995055	EF040686	-	-	DQ995147	DQ994978	AY875042
Plethodon sequoyah	DQ995056	EF040687	-	-	DQ995148	DQ994979	DQ018705
Plethodon serratus	DQ995057	-	EU275830	EU275876	DQ995149	DQ994980	DQ018671
Plethodon shenandoah-1	DQ995062	-	-	-	DQ995152	DQ994983	DQ018674
Plethodon shenandoah-2	-	-	-	-	DQ995153	DQ994984	-
Plethodon shermani	DQ995065	EF040688	-	-	DQ995156	DQ994987	DQ018674
Plethodon teyahalee	DQ995068	-	EU275834	EU275880	DQ995159	DQ994990	AY875030
Plethodon vandykei	AY691715	EF040689	EU275833	EU275879	AY691803	AY691759	-
Plethodon variolatus	DQ995070	-	-	-	DQ995161	DQ994992	AY875043
Plethodon vehiculum	AY691716	EF040690	-	-	AY691804	AY691760	DQ018661
Plethodon	DQ99507	-	-	-	DQ99516	DQ99499	DQ01868

ventralis	1				2	3	0
Plethodon virginia	DQ995072	EF040691	-	-	DQ995164	DQ994995	DQ018675
Plethodon websteri	DQ995073	-	-	-	DQ995165	DQ994996	DQ018682
Plethodon wehrlei	DQ995075	EF040692	-	-	DQ995169	DQ994998	DQ018687
Plethodon welleri	AY691717	EF040693	-	-	AY691805	AY691761	DQ018683
Plethodon yonahlossee-1	DQ995077	EF040693	EU275832	EU275878	DQ995171	DQ995000	DQ018706
Plethodon yonahlossee-2	AY691718	-	-	-	AY691806	AY691762	DQ018707

Additional file 6 references

Bonnet R.M., Chippendale P.T., Moler P.E., Van Devender R.W., Wake D.B. 2009. Evolution of gigantism in amphiumid salamanders. PLoS ONE 4:E5615.

Kozak K.H., Larson A., Bonett R.M., Harmon L.J. 2005. Phylogenetic analysis of ecomorphological divergence, community structure, and diversification rates in dusky salamanders (Plethodontidae: *Desmognathus*). Evolution 59:2000–2016.

Kozak K.H., Weisrock D.W., Larson A. 2006a. Rapid lineage accumulation in a non-adaptive radiation: phylogenetic analysis of diversification rates in eastern North American woodland salamanders (Plethodontidae: *Plethodon*). Proc. R. Soc. Lond. B. 273:539–546.

Kozak K.H., Blaine R.A., Larson A. 2006b. Gene lineages and eastern North American palaeodrainage basins: phylogeography and speciation in salamanders of the *Eurycea bislineata* species complex. Mol. Ecol. 15:191–207.

Vieites D.R., Min M.-S., Wake D.B. 2007. Rapid diversification and dispersal during periods of global warming by plethodontid salamanders. Proc. Natl. Acad. Sci. U.S.A. 04:19903–19907.

Weisrock D.W., Kozak K.H., Larson A. 2005. Phylogeographic analysis of mitochondrial gene flow and introgression in the salamander, *Plethodon shermani*. Mol. Ecol. 14:1457–1472.

Wiens J.J., Engstrom T.N., Chippindale P.T. 2006. Rapid diversification, incomplete isolation, and the “speciation clock” in North American salamanders (genus *Plethodon*): testing the hybrid swarm hypothesis of rapid radiation. Evolution 60:2585–2603.
