# Supplementary material for: What are the consequences of combining nuclear and mitochondrial data for phylogenetic analysis? Lessons from Plethodon salamanders and 13 other vertebrate clades
Source: BMC Evol Biol. 2011 Oct 13;11:300. doi: 10.1186/1471-2148-11-300 (PMC3203092; doi:10.1186/1471-2148-11-300)
Supplement: Additional File 7 — Branch length correlations among data types for each vertebrate clade. For each clade, all branches shared between a pair of trees (combined + mtDNA, combined + nucDNA, mtDNA + nucDNA) were tested for correlation. Nearly all comparisons show significant positive correlations in all possible combinations between the lengths of shared branches among trees. The two clades that do not show significant correlations in all combinations (dicaeid birds and cervid mammals) have very small sample sizes of shared branches, making detection of significant patterns difficult. [file 1471-2148-11-300-S7.PDF]

Additional file 7 – Branch length correlations among data types for each vertebrate clade. For each clade, all branches shared between a pair of trees (combined + mtDNA, combined + nucDNA, mtDNA + nucDNA) were tested for correlation. Nearly all comparisons show significant positive correlations in all possible combinations between the lengths of shared branches among trees. The two clades that do not show significant correlations in all combinations (Dicaeid birds and Cervid mammals) have very small sample sizes of shared branches, making detection of significant patterns difficult.

Clade	Combined vs. mtDNA	Combined vs. nucDNA	mtDNA vs. nucDNA
Balistid fish	r_s=0.909, P < 0.0001	r_s=0.885, P < 0.0001	r_s=0.833, P < 0.05
Scarine fish	r_s=0.825, P << 0.0001	r_s=0.922, P << 0.0001	r_s=0.792, P << 0.0001
Hemiphractid frogs	r_s=0.838, P << 0.0001	r_s=0.680, P < 0.0005	r_s=0.620, P < 0.01
Hylid frogs	r_s=0.867, P << 0.0001	r_s=0.805, P << 0.0001	r_s=0.435, P < 0.005
Plethodon salamanders	r_s=0.960, P < 0.0001	r_s=0.728, P < 0.0001	r_s=0.526, P < 0.03
Phrynosomatid lizards	r_s=0.938, P << 0.0001	r_s=0.894, P << 0.0001	r_s=0.738, P < 0.0005
Alcid birds	r_s=0.9925, P << 0.0001	r_s=0.870, P < 0.0001	r_s=0.802, P < 0.001
Caprimulgid birds	r_s=0.842, P << 0.0001	r_s=0.820, P << 0.0001	r_s=0.555, P < 0.005
Cotingid birds	r_s=0.907, P << 0.0001	r_s=0.928, P << 0.0001	r_s=0.811, P < 0.0001
Dicaeid birds	r_s=0.905, P << 0.0001	r_s=0.209, P = 0.473	r_s=-0.0545, P = 0.884

		(small sample size)	(small sample size)
Emydid turtles	r_s=0.946, $P << 0.0001$	r_s=0.465, $P < 0.05$	r_s=0.685, $P < 0.05$
Cervid mammals	r_s=0.896, $P << 0.0001$	r_s=0.433, ns (small sample size)	r_s=-0.143, ns (small sample size)
Murid rodents (Philippines)	r_s=0.789, $P << 0.0001$	r_s=0.820, $P << 0.0001$	r_s=0.675, $P < 0.0001$
Murid rodents (Sahul)	r_s=0.867, $P << 0.0001$	r_s=0.958, $P << 0.0001$	r_s=0.776, $P << 0.0001$
