# Supplementary material for: What are the consequences of combining nuclear and mitochondrial data for phylogenetic analysis? Lessons from Plethodon salamanders and 13 other vertebrate clades
Source: BMC Evol Biol. 2011 Oct 13;11:300. doi: 10.1186/1471-2148-11-300 (PMC3203092; doi:10.1186/1471-2148-11-300)
Supplement: Additional file 8 — Summary of data for 13 vertebrate clades. Supplementary Tables S1-S13. Summary of data for 13 vertebrate clades, including taxon sampling, length of gene, number of variable characters, number of parsimony informative characters, the best-fitting model of evolution, and the best-fitting partitions for each gene region. PDF file. [file 1471-2148-11-300-S8.PDF]

Additional file 8 – Summary of data for 13 vertebrate clades

Table S1. Summary of data for balistid fish from Dornburg et al. (2008), including taxon sampling, length of gene, number of variable characters, number of parsimony informative characters, the best-fitting model of evolution, and the best-fitting partitions for each gene region.

Type of data	Locus	Length	Variable characters	Parsimony informative characters	Best-fitting model	Partitions	Number of taxa sampled
nucDNA	Rhodopsin	564	110	66	HKY+I+Γ	gene	26
nucDNA	Tmo-4C4	575	81	41	HKY+I+Γ	gene	22
nucDNA	RAG-1	1471	332	202	GTR+I+Γ	gene	28
	Combined nucDNA	2610	523	309			
mtDNA	12S	833	262	180	GTR+I+Γ	gene, stems, loops	28

mtDNA	16S	563	172	131	GTR+I+Γ	gene, stems, loops	28
	Combined mtDNA	1396	434	311			

Table S2. Summary of data for scarine fish from Smith et al. (2008), including taxon sampling, length of gene, number of variable characters, number of parsimony informative characters, the best-fitting model of evolution, and the best-fitting partitions for each gene region.

Type of Data	Locus	Length	Variable characters	Parsimony informative characters	Best-fitting model	Partitions	Number of taxa sampled
nucDNA	rag2	779	332	233	SYM+I+Γ	gene	50
nucDNA	tmo4c4	485	165	126	HKY+Γ	gene	50
nucDNA	otx1	672	144	111	GTR+I+Γ	gene	50
nucDNA	bmp4	488	164	118	GTR+I+Γ	gene	50
nucDNA	dlx2	527	157	105	HKY+I+	gene	49

					Γ		
	Combined nucDNA	2951	962	693			
mtDNA	12S	980	429	349	GTR+I+Γ	gene	50
mtDNA	16S	578	207	173	GTR+I+Γ	gene	50
mtDNA	cyt-*b*	583	253	230	GTR+I+Γ	gene	49
	Combined mtDNA	2141	889	752			

Table S3. Summary of data for hemiphractid frogs from Wiens et al. (2007), including taxon sampling, length of gene, number of variable characters, number of parsimony informative characters, the best-fitting model of evolution, and the best-fitting partitions for each gene region.

Type of data	Locus	Length	Variable characters	Parsimony informative characters	Best-fitting model	Partitions	Number of taxa sampled

nucDNA	POMC	500	213	139	GTR+Γ	codon	43
nucDNA	RAG-1	1407	424	252	GTR+I+Γ	codon	45
	Combined nucDNA	1907	522	391			
mtDNA	12S	1040	531	403	GTR+I+Γ	stems, loops	50
mtDNA	ND1	820	513	443	GTR+I+Γ	codon, stems, loops	43
mtDNA	16S	602	233	175	GTR+I+Γ	stems, loops	46
	Combined mtDNA	2462	1434	1158			

Table S4. Summary of data for hylid frogs from Wiens et al. (2005), including taxon sampling, length of gene, number of variable characters, number of parsimony informative characters, the best-fitting model of evolution, and the best-fitting partitions for each gene region.

Type of data	Locus	Length	Variable characters	Parsimony informative	Best-fitting model	Partitions	Number of taxa sampled

				characters			
nucDNA	POMC	547	330	274	GTR+I+Γ	codon	79
nucDNA	c-myc	832	440	307	GTR+I+Γ	codon	80
	Combined nucDNA	1379	770	581			
mtDNA	12S	1078	693	558	GTR+I+Γ	stems, loops	80
mtDNA	ND1	1218	777	664	GTR+I+Γ	codon, structural, loops	77
	Combined mtDNA	2296	1470	1222			

Table S5. Summary of data for phrynosomatid lizards from Wiens et al. (2010b), including taxon sampling, length of gene, number of variable characters, number of parsimony informative characters, the best-fitting model of evolution, and the best-fitting partitions for each gene region.

Type of data	Locus	Length	Variable character	Parsimony	Best-fitting	Partitions	Number of taxa

			s	informative characters	model		sampled
nucDNA	BDNF	670	71	28	HKY+I+Γ	codon	16
nucDNA	ECEL	613	168	102	GTR+I+Γ	codon	35
nucDNA	PRLR	581	255	128	HKY+Γ	codon	38
nucDNA	PTPN	713	243	135	HKY+Γ	codon	36
nucDNA	RAG-1	1045	370	184	GTR+I+Γ	codon	38
nucDNA	TRAF6	557	151	91	HKY+Γ	codon	39
	Combined nucDNA	4179	1258	668			
mtDNA	12S	762	349	228	GTR+I+Γ	stems, loops	37
mtDNA	16S	470	172	122	GTR+I+Γ	stems, loops	35
mtDNA	ND1	969	410	288	GTR+I+Γ	codon	11
mtDNA	ND2	1705	978	780	GTR+I+	codon,	28

					Γ	stems, loops	
mtDNA	ND4	699	409	337	GTR+I+Γ	codon	32
	Combined mtDNA	4605	2318	1755			

Table S6. Summary of data for alcid birds from Pereira and Baker (2008), including taxon sampling, length of gene, number of variable characters, number of parsimony informative characters, the best-fitting model of evolution, and the best-fitting partitions for each gene region.

Type of data	Locus	Length	Variable characters	Parsimony informative characters	Best-fitting model	Partitions	Number of taxa sampled
nucDNA	Rag-1	2742	333	120	GTR+I+Γ	codon	26
	Combined nucDNA	2742	333	120			
mtDNA	12S	552	167	119	GTR+I+	codon	26

					Γ		
mtDNA	16S	1031	367	268	GTR+I+Γ	codon	26
mtDNA	COI	1080	378	315	GTR+I+Γ	codon	26
mtDNA	cyt-b	1002	375	298	GTR+I+Γ	codon	26
mtDNA	ND2	996	462	368	GTR+I+Γ	codon	26
	Combined mtDNA	4661	1749	1368			

Table S7. Summary of data for caprimulgid birds from Han et al. (2010), including taxon sampling, length of gene, number of variable characters, number of parsimony informative characters, the best-fitting model of evolution, and the best-fitting partitions for each gene region.

Type of data	Locus	Length	Variable characters	Parsimony informative characters	Best-fitting model	Partitions	Number of taxa sampled
nucDNA	cmyc	1318	363	211	GTR+I+	intron/ex	72

					Γ	on/UTR	
nucDNA	GH	1757	638	355	SYM+Γ	intron/exon	72
	Combined nucDNA	3075	1001	566			
mtDNA	cyt-*b*	1143	564	504	GTR+I+Γ	codon	72
	Combined mtDNA	1143	759	648			

Table S8. Summary of data for cotingid birds from Ohlson et al. (2007), including taxon sampling, length of gene, number of variable characters, number of parsimony informative characters, the best-fitting model of evolution, and the best-fitting partitions for each gene region.

Type of data	Locus	Length	Variable characters	Parsimony informative characters	Best-fitting model	Partitions	Number of taxa sampled
nucDNA	myo	746	289	141	GTR+Γ	gene	39
nucDNA	G3P	369	213	106	GTR+Γ	gene	39
	Combined nucDNA	1115	502	247			
mtDNA	cyt-b	999	496	435	GTR+I+Γ	gene	39

	Combined mtDNA	999	496	435

Table S9. Summary of data for dicaeid birds from Nyári et al. (2009), including taxon sampling, length of gene, number of variable characters, number of parsimony informative characters, the best-fitting model of evolution, and the best-fitting partitions for each gene region.

Type of data	Locus	Length	Variable characters	Parsimony informative characters	Best-fitting model	Partitions	Number of taxa sampled
nucDNA	TGFb2	542	162	58	HKY+Γ	none	42
	Combined nucDNA	542	162	58			
mtDNA	ND2	1032	581	495	GTR+I+Γ	codon	42
mtDNA	ND3	351	180	151	HKY+I+Γ	codon	42
	Combined mtDNA	1383	759	648			

Table S10. Summary of data for emydid turtles from Wiens et al. (2010a), including taxon sampling, length of gene, number of variable characters, number of parsimony informative characters, the best-fitting model of evolution, and the best-fitting partitions for each gene region.

Type of data	Locus	Length	Variable	Parsimony	Indel Charact	Best-fitting	Partitions	Number of taxa

			characters	informative characters	ers	model		sampled
nucDNA	ETS	758	129	47	10	GTR+Γ	none (intron)	30
nucDNA	GAPD	433	26	62	4	HKY+Γ	none (intron)	38
nucDNA	ODC	552	26	59	5	HKY+Γ	none (intron)	30
nucDNA	R35	946	63	28	0	HKY+Γ	none (intron)	26
nucDNA	VIM	741	202	76	15	GTR+Γ	none (intron)	40
nucDNA	NGFB	536	32	14	0	HKY+Γ	none	37
	Combined nucDNA	3966	659	225				
mtDNA	Cyt-b	648	236	164	0	HKY+I+Γ	codon	41
mtDNA	ND4	616	225	165	0	HKY+I	codon	39

					+Γ		
	Combined mtDNA	1264	531	353			

Table S11. Summary of data for cervid mammals from Gilbert et al. (2006), including taxon sampling, length of gene, number of variable characters, number of parsimony informative characters, the best-fitting model of evolution, and the best-fitting partitions for each gene region.

Type of data	Locus	Length	Variable characters	Parsimony informative characters	Best-fitting model	Partitions	Number of taxa sampled
nucDNA	αLAIb	477	112	35	HKY+Γ	gene	29
nucDNA	PRKCI	514	112	27	HKY+Γ	gene	29
	Combined nucDNA	991	224	62			
mtDNA	COII	1140	466	368	GTR+I+Γ	gene	29
	cyt-b	587	216	176	GTR+I+Γ	gene	29
	Combined mtDNA	1727	682	544			

Table S12. Summary of data for murid rodents (Philippines) from Jansa et al. (2005), including taxon sampling, length of gene, number of variable characters, number of parsimony informative

characters, the best-fitting model of evolution, and the best-fitting partitions for each gene region.

Type of data	Locus	Length	Variable characters	Parsimony informative characters	Best-fitting model	Partitions	Number of taxa sampled
nucDNA	IRBP	1233	670	568	GTR+I+Γ	codon	60
	Combined nucDNA	1233	670	568			
mtDNA	cyt-*b*	1137	640	499	GTR+I+Γ	codon	60
	Combined mtDNA	1137	640	499			

Table S13. Summary of data for murid rodents (Sahul) from Rowe et al. (2008), including taxon sampling, length of gene, number of variable characters, number of parsimony informative characters, the best-fitting model of evolution, and the best-fitting partitions for each gene region.

Type of data	Locus	Length	Variable characters	Parsimony informative characters	Best-fitting model	Partitions	Number of taxa sampled
nucDNA	GHR	945	419	243	GTR+I+Γ	codon	60
nucDNA	BRCA1	2710	1702	916	GTR+I+Γ	codon	54

nucDNA	Rag-1	3074	1069	624	GTR+I+Γ	codon	61
nucDNA	BDR	1122	631	328	GTR+I+Γ	codon	58
nucDNA	IRBP	1316	502	307	GTR+I+Γ	codon	57
nucDNA	AP5	435	213	158	GTR+I+Γ	codon	61
	Combined nucDNA	9602	4536	2576			
mtDNA	cyt-b	1140	572	503	GTR+I+Γ	codon	63
mtDNA	COI	474	198	158	GTR+I+Γ	codon, tRNA	52
mtDNA	COII	765	355	306	GTR+I+Γ	codon, tRNA	52
mtDNA	ATPase	120	79	60	GTR+I+Γ	codon	45
	Combined mtDNA	2499	1204	1027			
