# Supplementary material for: What are the consequences of combining nuclear and mitochondrial data for phylogenetic analysis? Lessons from Plethodon salamanders and 13 other vertebrate clades
Source: BMC Evol Biol. 2011 Oct 13;11:300. doi: 10.1186/1471-2148-11-300 (PMC3203092; doi:10.1186/1471-2148-11-300)
Supplement: Additional file 9 — MrBayes settings for additional data sets. All data followed the phylogenetic methods used for Plethodon except for total number of generations. The generations used for each data set that was reanalyzed for this study are listed below. Emydid turtles and phrynosomatid lizards were not reanalyzed because we had access to the MrBayes output files from the original studies. PDF file. [file 1471-2148-11-300-S9.PDF]

Additional file 9 - MrBayes settings for additional data sets.

All data followed the phylogenetic methods used for *Plethodon* except for total number of generations. The generations used for each dataset that was reanalyzed for this study are listed below. Emydid turtles and Phrynosomatid lizards were not reanalyzed because we had access to the MrBayes output files from their respective original study.

Clade	nucDNA generations	mtDNA generations	Combined-data generations
Balistid fish	10 million	10 million	20 million
Scarine fish	10 million	10 million	10 million
Hemiphractid frogs	3 million	3 million	3 million
Hylid frogs	6 million	6 million	6 million
Alcid birds	6 million	6 million	6 million
Caprimulgid birds	10 million	10 million	10 million
Cotingid birds	4 million	4 million	4 million
Dicaeid birds	10 million	10 million	10 million
Cervid mammals	4 million	6 million	6 million
Murid rodents (Philippines)	6 million	6 million	6 million
Murid rodents (Sahul)	10 million	6 million	15 million
